# Zwitterionic carbamate interfaces unlock efficient “liquid” CO_2_ upgrading

**DOI:** 10.1126/sciadv.aed8640

**Published:** 2026-05-13

**Authors:** Yitong Li, Peng Li, Yiwen Zhong, Xuan Cheng, Zhenfang Zhang, Yu Mao, Chongchong Wu, Dongyuan Liu, Lei Zhang, Diego Alejandro Patino Bedoya, Matthew David, Ziyun Wang, Tianyi Ma

**Affiliations:** ^1^ Centre for Atomaterials and Nanomanufacturing (CAN), School of Science, RMIT University, Melbourne, VIC 3000, Australia.; ^2^ ARC Industrial Transformation Research Hub for Intelligent Energy Efficiency in Future Protected Cropping (E2Crop), Melbourne, VIC, 3000, Australia.; ^3^ CSIRO Manufacturing, Clayton, VIC 3168, Australia.; ^4^ School of Chemical Sciences, University of Auckland, Auckland 1010, New Zealand.; ^5^ CNOOC Institute of Chemical and Advanced Materials (Beijing) Co. Ltd., Beijing 102209, P. R. China.; ^6^ Hexanium Pty Ltd., 39 Rankin Street, Indooroopilly, QLD 4068, Australia.; ^7^ Advanced Fuel Innovation Pty Ltd., Room 109/19 College Walk, Clayton, VIC 3800, Australia.; ^8^ GrapheneX Pty Ltd., Level 3A, Suite 2, 1 Bligh Street, Sydney, NSW 2000, Australia.

## Abstract

Integrating carbon dioxide (CO_2_) capture with its direct electrochemical conversion remains a major challenge for sustainable energy technologies. Although amine scrubbing is widely used for CO_2_ capture, its integration with electrochemical reduction is often inefficient due to poor mass transport and limited reactivity of captured CO_2_ species. Here, we report an integrated capture-conversion platform that enables direct electrolysis of amine-captured CO_2_ in liquid carbamate solutions. Screening structurally related amines identifies piperazine (PZ) as an effective capture agent that forms stable, highly concentrated carbamate species. When combined with a nickel-based catalyst and a designed gas-liquid-solid interface, the system enables efficient liquid-phase CO_2_ conversion, achieving up to 60% Faradaic efficiency for carbon monoxide (CO) and providing the first quantitative verification of carbamate participation (~40%). In a scaled electrolyzer (9 cm^2^), rapid CO_2_ capture and release kinetics enable CO Faradaic efficiencies of 30 to 45% and energy efficiencies of ~15 to 25% under ambient conditions, with stable operation over 150 hours.

## Teaser

Captured CO_2_ can be electrochemically converted into useful chemicals directly in liquid carbamate solutions.

## INTRODUCTION

**Fig. 1.**
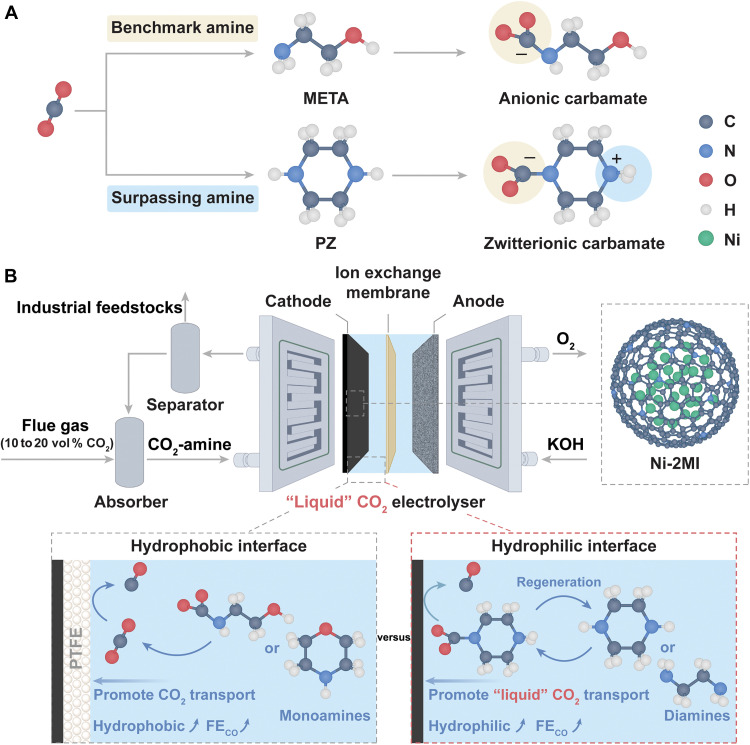
Capture chemistry and interfacial design for ICCE. (**A**) Comparison of carbamate formation pathways between benchmark (META) and surpassing (PZ) amines. (**B**) Schematic illustration of ICCE and electrode-electrolyte interfacial regulation of electroreduction.

The combustion of fossil fuels driven by anthropogenic activities has elevated atmospheric carbon dioxide (CO_2_) levels to ~420 parts per million (ppm) by 2025, contributing substantially to global climate change (*1*). Over 30% of these emissions originate from concentrated industrial processes, notably fossil fuel power generation, steel production, cement and fertilizer manufacturing, and petroleum refining, in which CO_2_ is typically present at concentrations of ~10 to 20 vol % in flue gas (*2*). Postcombustion CO_2_ capture from these high-emission sources represents a crucial mitigation strategy in the pursuit of global carbon neutrality by 2050 (*3*). As shown in Fig. 1, aqueous amine scrubbing—particularly with 20 to 30 wt % ethanolamine—remains the benchmark CO_2_ capture technology (Fig. 1A). However, this conventional method relies on energy-intensive regeneration (typically 120° to 150°C) to release captured CO_2_, followed by compression and purification steps, which collectively impose substantial energy penalties and induce amine degradation and secondary carbon emissions (*2*, *4*). To overcome these limitations, a tandem strategy has emerged wherein CO_2_ adducts formed during amine scrubbing are directly electrolyzed to value-added chemicals, eliminating the need for thermal regeneration, as illustrated fig. S1 (*5*, *6*). This integrated CO_2_ capture and electrolysis (ICCE) approach offers simplified process design, reduced energy input, and lower carbon footprints. However, most reported studies focus on ethanolamine-based systems, which display modest electrochemical activity with carbon monoxide Faradaic efficiencies (FE_CO_) typically below 30% (*6*). These findings raise an important question: Are benchmark amines, originally designed for thermal desorption, inherently suitable for direct electrochemical conversion?

The combustion of fossil fuels driven by anthropogenic activities has elevated atmospheric carbon dioxide (CO_2_) levels to ~420 parts per million (ppm) by 2025, contributing substantially to global climate change (*1*). Over 30% of these emissions originate from concentrated industrial processes, notably fossil fuel power generation, steel production, cement and fertilizer manufacturing, and petroleum refining, in which CO_2_ is typically present at concentrations of ~10 to 20 vol % in flue gas (*2*). Postcombustion CO_2_ capture from these high-emission sources represents a crucial mitigation strategy in the pursuit of global carbon neutrality by 2050 (*3*). As shown in Fig. 1, aqueous amine scrubbing—particularly with 20 to 30 wt % ethanolamine—remains the benchmark CO_2_ capture technology (Fig. 1A). However, this conventional method relies on energy-intensive regeneration (typically 120° to 150°C) to release captured CO_2_, followed by compression and purification steps, which collectively impose substantial energy penalties and induce amine degradation and secondary carbon emissions (*2*, *4*). To overcome these limitations, a tandem strategy has emerged wherein CO_2_ adducts formed during amine scrubbing are directly electrolyzed to value-added chemicals, eliminating the need for thermal regeneration, as illustrated fig. S1 (*5*, *6*). This integrated CO_2_ capture and electrolysis (ICCE) approach offers simplified process design, reduced energy input, and lower carbon footprints. However, most reported studies focus on ethanolamine-based systems, which display modest electrochemical activity with carbon monoxide Faradaic efficiencies (FE_CO_) typically below 30% (*6*). These findings raise an important question: Are benchmark amines, originally designed for thermal desorption, inherently suitable for direct electrochemical conversion?

Among emerging alternatives, the cyclic diamine piperazine (PZ) has demonstrated superior CO_2_ capture performance relative to ethanolamine. Literature evidence indicates that PZ could form zwitterionic carbamate species with reasonably high reaction rate, making it an excellent candidate in CO_2_ absorption (*7*). We therefore speculate whether this structure and the formed zwitterionic species could offer inherent electrolysis advantages over presumably used amines, such as ethanolamine. Notably, liquid-phase carbamate ions, such as those derived from PZ, can reach concentrations as high as 2 to 3 M—nearly two orders of magnitude higher than the dissolved CO_2_ concentration (~0.03 M) in typical gas-fed electrolysis systems like 0.5 M KHCO_3_. This exceptional ionic CO_2_ loading has the potential to overcome mass-transfer limitations at the gas-liquid-solid interface, ensure high local species availability (e.g., carbamate and/or CO_2_), and boost electrochemical reaction rates. Although ethanolamine is also known to form carbamate species, existing evidence suggests that these species do not actively participate in electroreduction, highlighting a gap in our understanding of the true electroactive species in such systems (*5*, *8*, *9*). This raises a key mechanistic question: Can carbamate ions directly serve as electron-accepting intermediates during electrolysis, and how does their reactivity correlate with amine molecular structure?

To address this knowledge gap, we systematically examine a set of six structurally distinct amines—monoethanolamine (META), propylamine (PLA), ethylenediamine (EDA), morpholine (MP), piperidine (PD), and PZ—comprising both linear and cyclic frameworks with varied functional symmetry. Using a carefully optimized Ni nanoparticle (NP) catalyst system, we demonstrate that PZ outperforms other amines in both CO_2_ capture and subsequent electrochemical conversion (Fig. 1B). Through a detailed understanding of the zwitterionic carbamate species formed in PZ/EDA systems, we construct a functional triple-phase boundary composed of Ni NPs and a hydrophilic gas diffusion layer via modulating polytetrafluoroethylene (PTFE) content, wherein modulation of the PTFE content makes possible the controlled conversion of either “liquid” CO_2_ (carbamates) or “gas” CO_2_ from captured solution into carbon monoxide. A comparative analysis reveals that selecting an appropriate CO_2_ carrier for interfacial delivery, rather than suppressing side reactants, is a more effective strategy, allowing suitable interfaces to reach an unprecedented FE_CO_ of 60% in PZ and EDA systems. Sustained electrolysis in a 9-cm^2^ zero-gap membrane electrode assembly (MEA) electrolyzer demonstrated the outstanding operational stability of the PZ system, maintaining performance under continuous feeding of a 20 vol % CO_2_ simulated flue gas stream for more than 150 hours, with a sustained average FE_CO_ of ~30 to 45%, single pass CO_2_-to-CO efficiency of ~20 to 42%, and energy efficiency (EE) of ~15 to 25%. Comprehensive energy analysis reveals a carbon monoxide (CO) production cost of 46.8 GJ per ton of CO, outperforming all the previously reported CO_2_ reduction technologies.

## RESULTS

### Comparative study of CO_2_ capture capacity and rate

**Fig. 2.**
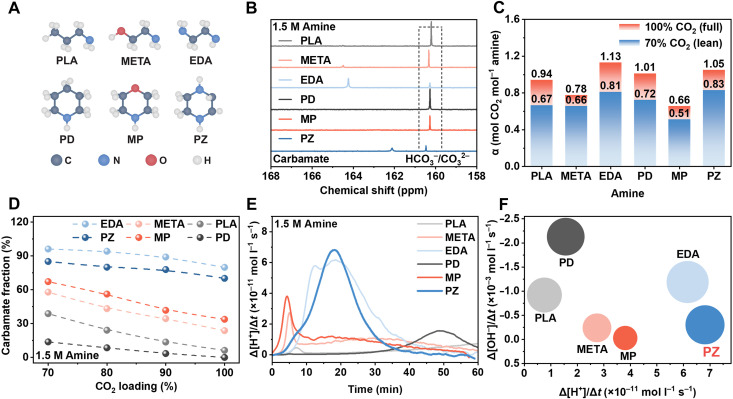
Comparative capture chemistry and kinetics of structurally distinct amines. (**A**) Structural formulas of the six amines: PLA, META, EDA, PD, MP, and PZ, where gray represents carbon atoms, red represents oxygen atoms, blue represents nitrogen atoms, and white represents hydrogen atoms. (**B**) ^13^C NMR spectra of the six amines in the high chemical shift range (158 to 168 ppm). The dashed box highlights a peak at ~160 ppm, attributed to bicarbonate/carbonate species (HCO_3_^−^/CO_3_^2−^). (**C**) α values (mol CO_2_/mol amine) of CO_2_ at 100 and 70% relative loading levels, with 100% defined as full loading and 70% as lean loading in this study. (**D**) Fraction of captured CO_2_ as carbamate. (**E**) Rate of proton change over time (Δ[H^+^]/Δ*t*) during the reaction between CO_2_ and amines in the bubble column. (**F**) Graph of capture rates corresponding to the capacity of the six amines, and the bubble size represents the capacity of the amine.

Ethanolamine (HO─CH_2_─CH_2_─NH_2_) aqueous solution (~20 to 30 wt %) is the first-generation CO_2_ absorbent that has been developed in the field of amine scrubbing for decades. Generally, alkylamines featuring amine functional groups (primary/secondary/tertiary) can capture CO_2_ gases, but with different reaction mechanisms and CO_2_ capture capacities, largely determined by their electronic structures and geometrical configurations (*4*). For instance, substituting the hydroxyl group (─OH) of ethanolamine (HO─CH_2_─CH_2_─NH_2_) with a methyl (─CH_3_) or a primary amine group (─NH_2_) can produce PLA (H_3_C ─CH_2_─CH_2_─NH_2_) or EDA (H_2_N─CH_2_─CH_2_─NH_2_). Similarly, MP [O(CH_2_CH_2_)_2_NH], a heterocyclic amine featuring an oxygen atom in its ring, can be structurally analogized by replacing the oxygen with a carbon or nitrogen atom to produce PD [(CH_2_)_5_─NH] and PZ [NH─(CH_2_)_4_─NH], respectively (*10*). Figure 2A compares the capture chemistry and kinetics of structurally distinct amines. As depicted in Fig. 2A, these six amines are classical representatives in the amine scrubbing field, and their nomenclature and abbreviations are provided (*4*). Their contrasting linear and cyclic frameworks, combined with distinct substitution patterns and symmetry, provide a valuable platform for investigating how molecular configuration influences both CO_2_ absorption capacity and downstream electrochemical conversion. Despite their longstanding relevance in capture technologies, a systematic evaluation of their performance across both capture and electrolysis processes has been lacking. Such an investigation is critical to advancing the field of ICCE. The reaction pathways of amine aqueous solutions and CO_2_ molecules are provided in Eqs. 1 to 3

$${\text{2R}}_{1}─R_{n}─{\text{NH}}_{2}+{\text{CO}}_{2}↔R_{1}─R_{n}─{{\text{NH}}_{3}}^{+}+R_{1}─R_{n}─\text{NH}─{\text{COO}}^{–}$$ (1)

$$R_{1}─R_{n}─{\text{NH}}_{2}+{\text{CO}}_{2}+H_{2}O↔R_{1}─R_{n}─{{\text{NH}}_{3}}^{+}+{{\text{HCO}}_{3}}^{-}$$ (2)

$$H_{2}N─R_{1}─R_{n}─{\text{NH}}_{2}+{\text{CO}}_{2}↔H_{3}N^{+}─R_{1}─R_{n}─\text{NH}─{\text{COO}}^{-}$$ (3)

Here, the four monoamines (PLA, META, MP, and PD) have similar CO_2_ capture pathways as described in Eqs. 1 and 2. Diamines (EDA and PZ) feature similar CO_2_ reaction mechanism with the formation of zwitterion amine carbamate ions, as evidenced in Eq. 3. To quantify CO_2_ capture capacity and speciation, we used ^13^C nuclear magnetic resonance (NMR) spectroscopy under both rich (~100%) and lean (~70%) CO_2_ loading conditions. The high-frequency chemical shifts (160 to 180 ppm) in Fig. 2B indicate the presence of carbamate and bicarbonate/carbonate species. Detailed analysis in Fig. 2C, note S1, figs. S2 to S8, and tables S1 to S6 reveal that the normalized CO_2_ capture capacities (rich/lean) for PLA, META, EDA, PD, MP, and PZ are 0.94/0.67, 0.78/0.66, 1.13/0.81, 1.01/0.72, 0.66/0.51, and 1.05/0.83 mol CO_2_ per mol amine, respectively. Notably, Fig. 2D shows that under 70 to 100% loading, carbamate formation dominates in PZ and EDA, while in monoamines, carbonate species are more prevalent, suggesting distinct mechanistic pathways depending on the amine structure. To evaluate the dynamic performance of capture agents relevant to ICCE, we measured the CO_2_ absorption kinetics using pH-time profiles in a bubble column setup (note S1 and figs. S9 and S10) (*11*). Linear and cyclic amines with comparable functional groups exhibit similar trends in [H^+^] evolution over time (Fig. 2E). Benchmark META and MP exhibit moderately fast reaction kinetics during the initial carbamate formation stage, driven by nucleophilic addition. However, their capture rates diminish markedly in the later stages, primarily due to the sluggish kinetics of carbamate hydrolysis (*12*). In contrast, PLA and PD present the slowest overall CO_2_ capture rates, primarily due to their poor ability to form stable carbamates, which readily hydrolyze into carbonate during the capture process (*13*). In particular, diamines such as EDA and PZ, each having two nucleophilic nitrogen sites, maintain consistently high CO_2_ absorption rates throughout the entire capture process. This performance is corroborated by ^13^C NMR data, which confirm that 70 to 80% of the captured CO_2_ exists in the form of ionized amine-CO_2_ species, indicating a robust and sustained carbamate-based capture mechanism. Among all tested amines, PZ exhibits the highest Δ[H^+^]/Δ*t*, establishing the kinetic performance ranking as PZ > EDA > MP > META > PD > PLA. This order is in agreement with previous qualitative evaluations, reinforcing that diamine delivers the most efficient and rapid CO_2_ capture under equivalent operating conditions (*14*–*17*). Plotting Δ[H^+^]/Δ*t*, Δ[OH^−^]/Δ*t*, and CO_2_ capacity of the six amines results in the relationship as shown in Fig. 2F, where we can find that PZ and EDA have a fast CO_2_ capture rate along with a high CO_2_ loading capacity. Therefore, PZ and EDA exhibit considerably better capture performance compared with META and all other tested monoamines (*5*, *8*), thereby establishing them as particularly promising candidates for ICCE. These results strongly motivated us to further explore how amine structural properties influence the subsequent electrolysis performances of amine-captured CO_2_ solutions.

### Tandem reduction assessment

**Fig. 3.**
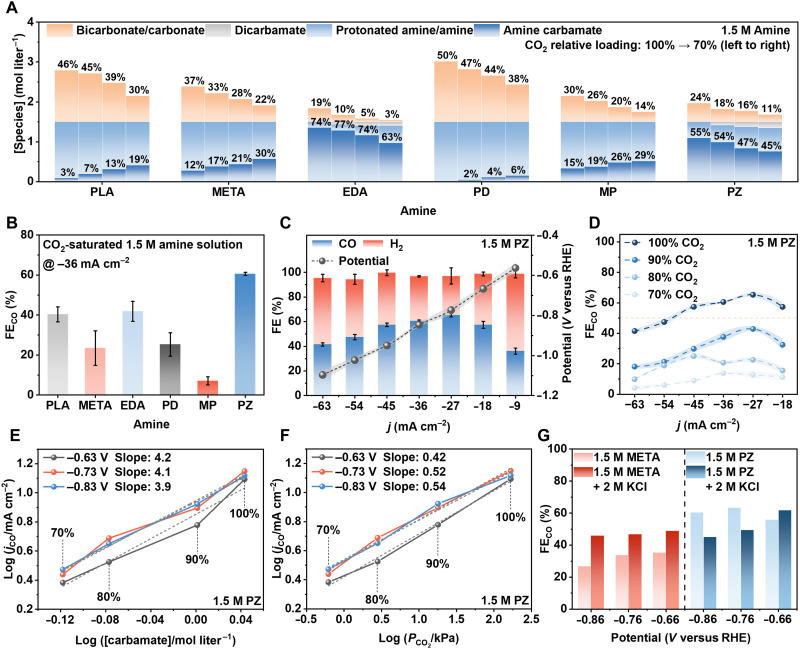
Tandem electrolysis of amine-captured CO_2_. (**A**) Ion species concentrations for PLA, META, EDA, PD, MP, and PZ. The labeled values denote the fractions of carbamate and bicarbonate/carbonate at CO_2_ relative loading levels of 100, 90, 80, and 70%, arranged from left to right, respectively. (**B**) FE_CO_ during chronopotentiometry at −36 mA cm^−2^ across six amines. (**C**) FE for CO and H_2_ measured in 1.5 M PZ under full CO_2_ loading at different current densities, together with the corresponding potentials (versus RHE). (**D**) FE_CO_ at four different CO_2_ loadings in 1.5 M PZ, measured at current densities ranging from −18 to −63 mA cm^−2^. (**E** and **F**) Logarithmic correlations between CO current density and (E) carbamate concentration and (F) CO_2_ partial pressure at different CO_2_ loadings. (**G**) Comparison of FE_CO_ over the potential range of −0.66 to −0.86 V for 1.5 M PZ and 1.5 M META solutions, before and after the addition of 2 M K^+^.

The tandem electrolysis of amine-captured CO_2_ is summarized in Fig. 3. Before the electrolysis studies, the structural properties and speciation conditions of the six amine solutions along CO_2_ loading are shown in Fig. 3A, fig. S8, tables S3 to S7, and note S1. At full CO_2_ loading (~100% loading), the dominant CO_2_-containing species include amine carbamate ions, amine ammonium ions, bicarbonate/carbonate ions, dissolved CO_2_, and water molecules. Accordingly, CO_2_ is stored in solution predominantly in chemically bound forms, mainly as carbamate and bicarbonate/carbonate species. Diamines such as PZ and EDA exhibit a pronounced preference for carbamate formation, with carbamate species accounting for 70.1 and 79.9% of the chemisorbed CO_2_, respectively. By contrast, monoamines including PLA, META, PD, and MP predominantly form bicarbonate/carbonate species, which constitute 93.7, 76.2, 100, and 66.3% of the chemisorbed CO_2_, respectively. For PZ and EDA, the presence of two nucleophilic nitrogen atoms facilitates the formation of zwitterionic carbamate structures, including protonated amine-carbamate species, as confirmed by both ^13^C NMR and in situ attenuated total reflectance–Fourier transform infrared (ATR-FTIR) spectroscopy (fig. S11). These distinct speciation patterns are hypothesized to have notable implications for subsequent CO_2_ electrolysis performance. Following a systematic catalyst screening and optimization process (figs. S12 to S21, table S8, and notes S2 and S3), Ni NPs supported on nitrogen-doped carbon derived from 2-methylimidazole (Ni-2MI) were identified as the optimal electrocatalyst (*18*). Using Ni-2MI in the electrolysis of the amine-captured CO_2_ solutions yields polarization curves and FE_CO_, as shown in Fig. 3 (B and C), fig. S22, and notes S3 and S4. PZ-captured CO_2_ solutions exhibit superior electrochemical performance, with FE_CO_ values ranging from ~30 to 60% over a wide potential window [−0.56 to −1.10 V versus reversible hydrogen electrode (RHE)], substantially outperforming other amine systems. At a moderate current density of −36 mA cm^−2^, the descending order of FE_CO_ is PZ > EDA > PLA > META ≈ PD > MP. To further investigate this performance, we prepared a series of PZ-captured CO_2_ solutions under varied loading conditions (PZ-90%, PZ-80%, and PZ-70%). As shown in Fig. 3D and fig. S22, the FE_CO_ increases with CO_2_ loading, reaching a peak at full loading conditions (PZ-100%, ~1.05 mol CO_2_ mol_PZ_^−1^). To clarify the mechanistic origins, correlations between key species—carbamate concentration and CO_2_ partial pressure (*P*_CO2_)—and CO partial current density (*j*_CO_) are provided (Fig. 3, E and F, fig. S23, and tables S9 and S10). Specifically, *j*_CO_ presents first-order relationship with carbamate concentration, and the slopes are determined to be ~3.9 to 4.2 at a wider potential range (approximately −0.6 to −0.9 V versus RHE). In sharp contrast, the slopes between *j*_CO_ and *P*_CO2_ range from 0.42 to 0.54, showing a remarkably weaker dependence on *P*_CO2_. These results suggest that the *j*_CO_ is highly correlated with carbamate concentration rather than *P*_CO2_, indicating the unique carbamate participation mechanism for electrolyzing PZ-captured CO_2_ system. This mechanistic insight is further validated through the addition of 2 M KCl to both PZ and benchmark META systems. In the META system, KCl addition reduces solution and charge transfer resistances and enhances FE_CO_ by ~20%, consistent with earlier findings on alkali-assisted CO_2_ reduction (*5*). However, for the PZ system, KCl addition primarily reduces resistances without considerably altering FE_CO_ (Fig. 3G, fig. S24, and table S4). This contrast highlights the fundamentally different electrochemical behavior of the PZ-captured CO_2_ system, which operates under a distinct carbamate-participation mechanism, unlike the bicarbonate-dominated META system.

**Fig. 4.**
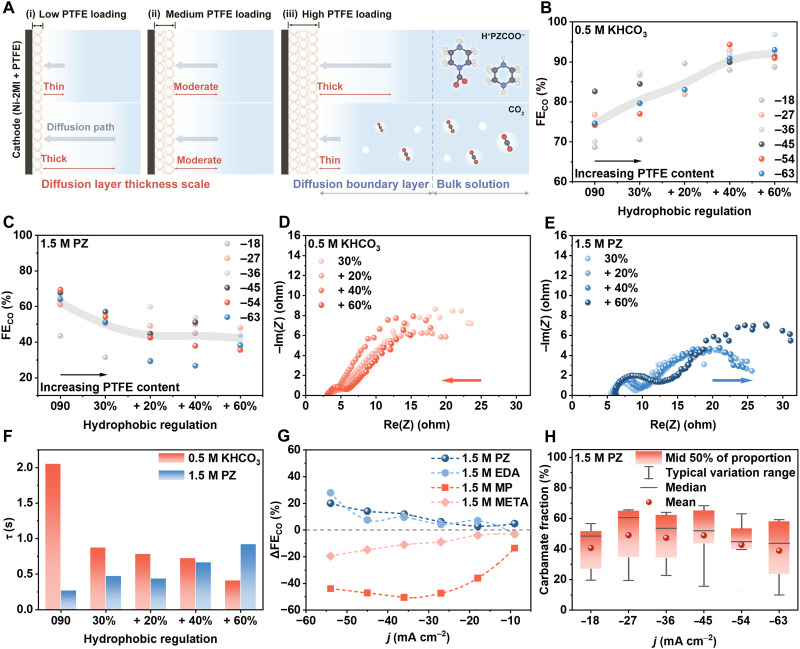
Interfacial regulation of mass transport and CO selectivity by PTFE incorporation. (**A**) Schematic illustration of PTFE-induced modulation of δ at the electrode surface. (**B** and **C**) FE_CO_ under different PTFE contents in 0.5 M KHCO_3_ (B) and 1.5 M PZ solution (C) at constant current densities of −18, −27, …, and −63 mA cm^−2^ (as labeled). “090” refers to TGP-H-090 with 5% PTFE carbon paper, and “30%” indicates TGP-H-090 with 30% PTFE carbon paper. “+20%,” “+40%,” and “+60%” represent the additional PTFE content introduced in the catalyst layer on TGP-H-090 30% PTFE carbon paper. (**D** and **E**) EIS plots and their equivalent circuits in 0.5 M KHCO_3_ (D) and 1.5 M PZ (E) solutions with varying PTFE contents. (**F**) τ corresponds to different PTFE contents in 0.5 M KHCO_3_ and 1.5 M PZ-CO_2_ solutions. (**G**) Comparison of FE_CO_ between hydrophilic and hydrophobic electrodes across four amines (ΔFE = FE_CO_ on hydrophilic electrode – FE_CO_ on hydrophobic electrode). (**H**) Fraction of PZ-CO_2_ involved in the reduction reaction under different applied currents in 1.5 M PZ CO_2_ solution.

We next further elucidated the nature of the reactive species involved in the electrolysis of amine-CO_2_ systems, carbamate ions versus gaseous CO_2_ molecules, according to their difference in mass transfer kinetics and examined whether interfacial functional tuning can selectively bias the reaction pathway (Fig. 4). Specifically, amine carbamate ions are liquid species that are distinct from gaseous CO_2_ molecules. To probe the difference, we engineered working electrodes by incorporating varying amounts of hydrophobic PTFE into Ni-2MI catalyst layers (Fig. 4A). PTFE addition is well known to strengthen local gas-phase CO_2_ concentration, enhance gas diffusion, and improve gas adsorption, leading to superior current density and selectivity in conventional gas-phase CO_2_ electrolysis systems (*19*, *20*). In contrast, increased PTFE content simultaneously impedes the transport of liquid-phase reactants, thereby reducing the local concentration of dissolved species such as carbamates (*21*). As a benchmark, we conducted a proof-of-concept experiment in gas CO_2_ electrolysis system [0.5 M KHCO_3_ under a CO_2_ flow rate of 20 standard cubic centimeter per minute (SCCM)] using Ni-2MI as the catalyst. As shown in Fig. 4B, the inclusion of PTFE (Toray paper TGP-H-090 with 30% PTFE versus TGP-H-090 with 30% PTFE coated with a catalyst layer containing an additional *x*% PTFE) contributes to a steady increase of FE_CO_ across a wider potential range from −0.5 to −1.0 V versus RHE, consistent with established results in gas-fed CO_2_ reduction systems (*19*, *20*). We then conducted identical tests in the electrolysis of 1.5 M PZ-captured CO_2_ solutions. In Fig. 4C, the FE_CO_ is found to decrease from ~65% with unmodified TGP-H-090 carbon paper to ~50% with 30% PTFE and further down to ~40% with 60% PTFE. This trend contradicts the behavior observed in gas-fed systems and suggests that the dominant reactive species in the PZ system are liquid-phase carbamate ions rather than CO_2_ gas molecules. The reduced performance upon increasing PTFE content strongly supports the hypothesis that mass transport of liquid carbamate species is hindered by the hydrophobic barrier introduced by PTFE, thus limiting their electrochemical accessibility. To quantify this observation, electrochemical impedance spectroscopy (EIS) combined with equivalent-circuit analysis was used to probe diffusion-related transport at the catalyst interface following PTFE addition. We extract the characteristic time constant (τ), which reflects the timescale of interfacial mass transport (Eq. 4). As τ scales with the diffusion boundary layer thickness (δ), a smaller τ corresponds to a thinner diffusion layer and, according to the limiting-current relationship, more favorable electrochemical performance (*20*, *22*)

$$\tau =R_{d}C_{d}$$ (4)

where *R*_d_ and *C*_d_ represent the resistance and capacitance of the double-layer, respectively, extracted from fitting the EIS equivalent circuit (*22*). Detailed calculation processes can be found in Fig. 4 (D and E), notes S4 and S5, figs. S25 and 26, and tables S10 to S12. Figure 4 (D and E) shows EIS spectra and corresponding equivalent circuits for Ni-2MI, illustrating the effect of PTFE incorporation in the catalyst layer across two reduction systems. Within the framework of a Randles-type equivalent-circuit model, the semicircle observed in the low- to mid-frequency region is associated with the charge-transfer resistance at the electrode/electrolyte interface. An apparent increase in the extracted *R*_ct_ is observed in PZ-CO_2_ solutions as the PTFE content in the electrode is gradually increased. This phenomenon is totally opposite to the results observed in the KHCO_3_ system (*19*, *20*). In addition, as shown in Fig. 4F, τ increases from 0.27 to 0.93 s with increasing PTFE content in the 1.5 M PZ-CO_2_ solution, whereas it decreases from 2.05 to 0.42 s in the 0.5 M KHCO_3_.

The decreasing trend in δ in the KHCO_3_ with increasing PTFE content, as reflected by τ, is also consistent with previous studies (*19*, *20*). This further demonstrates that the reactant species in the electrolysis of the PZ-captured CO_2_ system follow liquid mass-transfer dynamic behavior, in contrast to gas-phase CO_2_ reduction. It is reasonable to conclude that the liquid PZ carbamate species (PZCOO^−^/H^+^PZCOO^−^) are dominant reactant species that contribute to CO_2_-to-CO reaction in the PZ-captured CO_2_ system. To further generalize these observations, we extended the study to other amine captured CO_2_ systems including META, EDA, and MP. As shown in Fig. 4G, fig. S27, tables S13 and S14, and note S6, the effect of PTFE incorporation varied by capture agent. For META- and MP-captured CO_2_ solutions, FE_CO_ increases with PTFE content, mirroring the trend in the KHCO_3_ system and suggesting that molecular CO_2_ gas remains as the dominant reactive species (*5*, *9*). In the case of MP, this effect is especially pronounced under high overpotential conditions (−0.8 V versus RHE), where FE_CO_ increases from ~0 to ~45%, reinforcing the hypothesis that gas-phase CO_2_ is actively reduced in this system. Meanwhile, the decrease in FE_CO_ in EDA and PZ systems confirms that the amine carbamate species (zwitterion) participate in the electrolysis of PZ and EDA captured CO_2_ systems. Notably, in Fig. 4H, the fraction of reactive zwitterionic carbamate species in the PZ system is estimated to range from 20 to 60%, which is also dependent on the potential range. Detailed reactant species and pathways in the electrolysis of these amine captured CO_2_ systems are compared in Table 1. Hydrophobic interfacial functionalization, exemplified in monoamine-CO_2_ systems, was further used to repel competing Hydrogen Evolution Reaction (HER)-active species from the electrode surface, thereby promoting preferential CO_2_ occupancy and enabling tandem reduction pathways. A comparative analysis of hydrophobic versus hydrophilic interfaces, and monoamine versus diamine systems, revealed that protonated PZ carbamate, serving as a “liquid” CO_2_ carrier, is the critical enabler of direct captured CO_2_ reduction, rather than interfacial exclusion of competing species.

**Table 1.** Comparison of electrolysis performance across different amine-captured CO_2_ systems with increasing PTFE content.

Amine	FE_CO_ trend	Dominant reactant species	Mass transfer behavior	Key observation
META	Increased from ~40 to ~60%	Dissolved/gaseous CO_2_	Gas-phase enhanced	Charge transfer resistance is decreased.
EDA	Decreased from ~60 to 40%	Liquid-phase carbamate species (EDACOO^−^/H^+^EDACOO^−^)	Liquid-phase limited	Charge transfer resistance is increased.
MP	Increased from ~0 to ~40%	Dissolved/gaseous CO_2_	Gas-phase enhanced	The most pronounced decrease in charge transfer resistance and increase in FE_CO_ is observed.
PZ	Decreased from ~65 to ~40%	Liquid-phase carbamate species (PZCOO^−^/H^+^PZCOO^−^)	Liquid-phase limited	τ increases from 0.27 to 0.93 s.

### Mechanistic and computational study

**Fig. 5.**
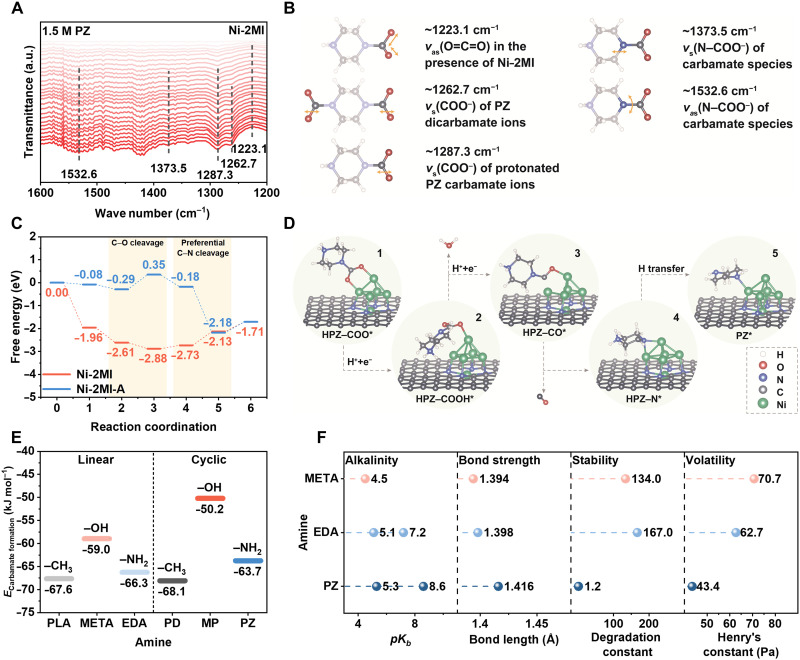
Mechanistic insights into carbamate electroreduction in amine–CO_2_ systems. (**A**) In situ ATR-FTIR spectra in 1.5 M PZ solution during electrolysis of PZ-captured CO_2_ solution, focusing on the 1200 to 1600 cm^−1^ range. The color gradient from light red to red corresponds to the applied potential change from 0 to −2.5 V versus RHE. a.u., arbitrary unit. (**B**) The peak positions and corresponding vibrational assignments of the in situ ATR-FTIR spectra were determined for the PZ solution. (**C**) Free energy profiles along different reaction coordinates for reaction pathway. (**D**) Adsorption configurations at five coordinations (1 to 5) of the reaction pathway at the interface. Ball-and-stick model with color code: white, H; red, O; blue, N; gray, C; green, Ni. (**E**) *E*_carbamate formation_ comparison for the six amines during the carbamate formation process. (**F**) Comparative analysis of PZ and EDA in terms of alkalinity, bond strength, stability, and volatility, illustrating key differences in their physical and chemical behaviors.

We made a further step to reveal how PZ carbamate species participates in the reduction reaction using in situ spectra technique and computational calculations (Fig. 5). As shown in note S7 and fig. S11, the evolved in situ ATR-FTIR spectra during CO_2_ bubbling into an aqueous PZ solution clearly capture the sequential formation of key reactive species. A pronounced band at ~1273.2 cm^−1^ emerges at early stages of CO_2_ introduction, corresponding to the formation of PZ carbamate ion (PZCOO^−^). With continued CO_2_ loading, two additional bands at ~1261.7 and ~1288.6 cm^−1^ appear, which can be attributed to carbamate (^−^OOCPZCOO^−^) and protonated carbamate species (H^+^PZCOO^−^), respectively, as supported by simulated results and reaction pathway (Eq. 3, note S7, and table S15). Peak variations for our PZ aqueous solution during CO_2_ bubbling can be also confirmed from reported experimental studies (*23*, *24*), demonstrating the formed PZ carbamate species and their evolutions with changed CO_2_ loadings. The presence of the ~2342 cm^−1^ band throughout the bubbling process is characteristic of the dissolved CO_2_ molecules, again confirming the CO_2_ saturation in the system. On this basis, we further tested the in situ ATR-FTIR spectra in the electrolysis of PZ-captured CO_2_ solution (PZ-100%, ~1.05 mol CO_2_ mol_PZ_^−1^) with Ni-2MI versus N-doped carbon (metal free) as the catalyst (Fig. 5, A and B, figs. S28 to S30, and note S7). When using Ni-2MI as the catalyst, distinct peaks at ~1287.3 and ~1532.6 cm^−1^ are observed at applied potentials up to −2.5 V, attributed to asymmetric carboxylate (ν_as_ COO^−^) and C─N (ν_as_ N─COO^−^) stretching modes from adsorbed H^+^PZCOO^−^ species. Additional stretching peaks at ~1489.2 and ~2342.3 cm^−1^ can be ascribed to vibrations of protonated amine ions and dissolved CO_2_ molecules. This again evidence that the H^+^PZCOO^−^ species and CO_2_ molecules are adsorbed at the catalyst’s surface and participate in the reduction reaction, which also aligns well with our mass transfer studies in Fig. 4.

ATR-FTIR evidence of ion species adsorption on Ni-2MI in PZ solution motivated further mechanistic exploration of its catalytic performance, and we performed density functional theory (DFT) calculations to reveal the underlying reaction pathways (note S8). To mechanistically support the observed reactivity of the PZ carbamate species, we constructed and analyzed a multilayered catalyst model. The optimized catalyst architecture, constructed on the basis of transmission electron microscopy (TEM) imaging and electronic-structure analysis of Ni-2MI, features an outer shell of N_4_-doped graphene substrate decorated with a Ni_6_ atomic cluster, which was identified by screening Ni*_n_* clusters (*n* = 3 to 20) supported on an inner graphene substrate interfaced with trilayer Ni NPs in a face-centered cubic structure (note S8 and figs. S31 to 33). After validating the structural property of this model, we systematically evaluated the thermodynamic profiles and reaction coordinates of key intermediates. Taking Ni-2MI as a representative model, the protonated PZ carbamate ions are first adsorbed on the Ni cluster surface with the probable formation of two Ni─O bonds. Subsequently, a proton-coupled electron transfer step occurs, in which a hydrogen atom combines with HPZ─COO* to form the HPZ─COOH* intermediate. This is followed by a multielectron transfer process that cleaves the C─O bond and releases OH^−^, forming HPZ─CO* as a stabilized intermediate on the catalyst surface. Subsequently, HPZ─CO* undergoes two competing pathways: C─N bond cleavage or intramolecular proton transfer to the *para*-nitrogen. Free energy calculations revealed that the cleavage of C─N bond is an exothermic process with a relatively low free energy change (0.60 eV). In contrast, intramolecular proton transfer to the *para*-nitrogen exhibits a considerably higher free energy change of 1.50 eV, suggesting that the C─N bond cleavage is energetically more favorable than restoring the original PZ structure. Detailed free energies and the reaction coordinates are presented in Fig. 5 (C and D) (reduction pathway on Ni-2MI shown in figs. S34 to S36 and tables S16 and S17). From the experimental results, Ni-2MI exhibited better experimental performance than Ni-2MI-A in PZ-CO_2_ reduction. To clarify this difference, a modified catalyst configuration was constructed by removing surface clusters while retaining the Ni─N_4_ site (Ni-2MI-A, corresponding to single-atom Ni species, obtained via acid washing of Ni-2MI). For the Ni-2MI model, the potential determining step occurs during the coordination between the fourth and fifth intermediate (C─N bond cleavage), with a relatively small free energy difference (approximately −0.6 eV). In contrast, the C─O cleavage is the rate determining step for Ni-2MI-A. Unlike the single-atom configuration of Ni-2MI-A, Ni-2MI features surface Ni clusters that offer dual coordination sites, enabling strong bidentate binding with protonated PZ carbamate species, as exemplified by the HPZ─COO* intermediate. These cluster-absorbate interactions markedly stabilize reaction intermediates and reduce the overall free energy, highlighting the critical role of cluster in steering the reaction pathway. Notably, DFT simulations reveal a distinct reaction pathway on Ni-2MI in the PZ-CO_2_ system, where the reduction is initiated by electron transfer to form a HPZ─COOH* adduct prior to C─N bond cleavage, in contrast to conventional integrated amine-CO_2_ reduction pathways in which CO_2_ is first released via C─N bond dissociation before undergoing electrochemical reduction (*9*).

We next studied why PZ exhibits better electrolysis performance than EDA given that their similar diamine structures and zwitterion species conditions. We first used Gaussian calculation and obtained the carbamate formation energy (*E*_formation_) values for these amines. Carbamate formation is essentially a reverse process of a C─N bond cleavage, which can be seen as an indicator to evaluate the difficulty in the carbamate electrolysis process. As shown in Fig. 5 (E and F), note S9, fig. S37, and table S18, PZ exhibits a moderate carbamate *E*_formation_ among the six candidates. Formation energies that are comparatively lower favor carbamate hydrolysis to bicarbonate/carbonate ions (e.g., PLA and PD), while comparatively higher values correspond to overly stable C─N bonds (e.g., META and MP), in good agreement with the experimentally observed CO_2_ capture behavior (note S1). Apart from carbamate *E*_formation_, additional factors also play important roles in ICCE. First, the base dissociation constant (p*K*_b_) of EDA is lower than that of PZ, indicating the stronger basic property of EDA and its higher tendency to form a rather stable EDA-CO_2_ adduct. This is supported by Gaussian calculations showing a shorter N─C bond length in the EDA-CO_2_ adducts, indicating a higher energy requirement for bond dissociation (breakage). Second, EDA molecules suffer from serious stability issues due to its strong degradation tendency via either an intermolecular cyclization pathway or a nucleophilic reaction pathway, which can be evidenced by its noticeably higher degradation constant (167.0; Fig. 5F) (*16*, *25*). The serious degradation would badly affect the electrolysis of EDA carbamate species and also largely restrict its continuous use under the harsh postcombustion capture conditions (*25*). Third, EDA molecules have a relatively high Henry’s constant and higher volatility than the PZ counterpart. During the industry-demanded applications, amine loss will be a serious issue to affect the system reliability and overall production cost (*26*). The aforementioned factors validate the excellence of PZ as a capture media in ICCE field compared to other presumable amines, again demonstrating the rationality of our amine screening concept and the joint catalyst design strategy.

### Evaluation in zero-gap electrolyzer

**Fig. 6.**
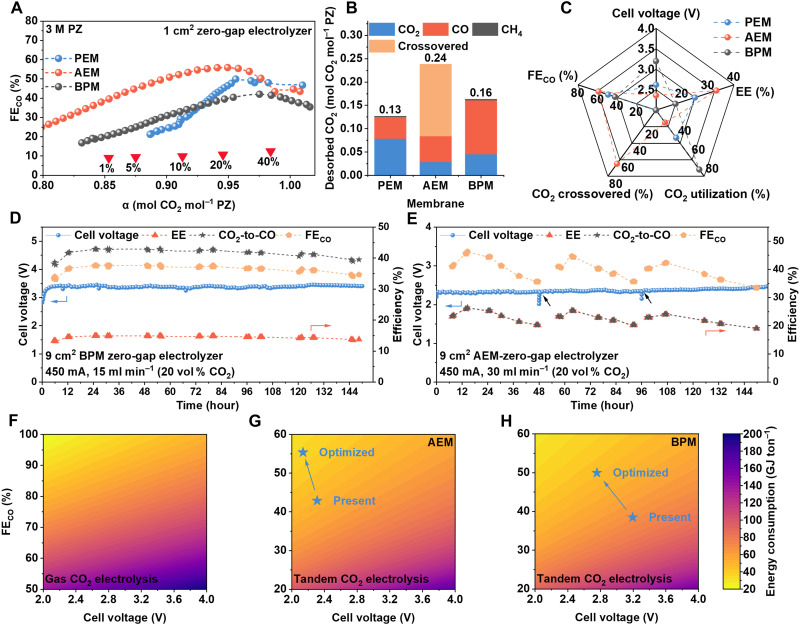
System-level performance and energy analysis of ICCE. (**A**) FE_CO_ as a function of CO_2_ loading under three membrane types. The values 1, 5, 10, 20, and 40% indicate the CO_2_ volume fractions (balanced with N_2_) in the feed gas stream, under which the corresponding CO_2_ loading values in 3 M PZ at saturation were determined. (**B**) Carbon distribution pathways during electrolysis, evaluated from full loading to the point where FE_CO_ dropped to ~20%. The values above each bar represent the total desorbed CO_2_ amount from the saturated 3 M PZ solution. (**C**) Radar charts for the multidimensional evaluation of integrated CO_2_ reduction performance using three different ion-transport membranes. (**D** and **E**) Long-term electrolysis performance of PZ-CO_2_ system over 120 hours under simulated flue gas using BPM and AEM, demonstrating system durability and consistent CO output. The arrow indicates the point at which the catholyte was resaturated with 20 vol % CO_2_, while the anolyte was replaced with fresh electrolyte. (**F** to **H**) Energy analysis of (F) conventional gas-phase CO_2_ electrolysis, and ICCE via (G) AEM-based and (H) BPM-based systems.

We next used zero-gap MEA electrolyzer cells (1 and 9 cm^2^; Fig. 6, note S10, figs. S38 to S47, and table S19) to verify the scalability of the integrated CO_2_ capture and electrolysis process (*27*). Electrolysis was performed using a 3 M PZ-captured CO_2_ solution as catholyte, and either 2 M KOH or 0.05 M H_2_SO_4_ as the anolyte, depending on the type of ion-exchange membrane used. Proton exchange membrane (PEM), anion exchange membrane (AEM), and bipolar membrane (BPM) were systematically evaluated to understand the influence of ion transport pathways on integrated CO_2_ utilization. To further assess the CO_2_ capture capability of PZ under dilute conditions, we measured the CO_2_ loading in 3 M PZ under saturation using CO_2_/N_2_ gas (simulated flue gas) mixtures containing 100, 40, 20, 10, 5, and 1 vol % CO_2_ (balanced with N_2_). The corresponding CO_2_ loadings were determined to be 1.02, 0.98, 0.95, 0.91, 0.87, and 0.85 mol CO_2_ mol_PZ_^−1^, respectively (Fig. 6A and fig. S38). The durability and reliability of both the catalysts and the overall system were further demonstrated through two distinct operational modes: electrolysis under continuous CO_2_ bubbling, and electrolysis transitioning from a CO_2_-saturated to a CO_2_-lean state. Long-term electrolysis tests for up to 200 hours show no appreciable current decay, affirming excellent catalyst and system stability (figs. S40 to S44). The choice of ion-transport membrane strongly affects the overall electrochemical performance during integrated CO_2_ capture and conversion. As shown in Fig. 6A, we evaluated the evolution of FE_CO_ from full to lean CO_2_ loading. Among the three membranes tested (Fig. 6B), the AEM exhibited superior electrochemical performance, delivering an FE_CO_ of ~60% at full loading and maintaining ~50% within the simulated flue gas range (10 to 20 vol % CO_2_), while sustaining a stable cell voltage around 2.36 V. In contrast, while BPM resulted in slightly lower FE_CO_ values, it enabled a notably higher CO_2_-to-CO conversion efficiency, reaching up to 72.2%. A comparative analysis of the three membrane types and representative absorbents (including amino acid salts and carbonates) is presented in Fig. 6C and Table 2. Even in comparison with other state-of-the-art reactive capture systems using alternative absorbents, PZ not only demonstrates a high CO_2_ gas uptake rate and large capture capacity but also outperforms in its electrochemical conversion efficiency. We further scaled our 1-cm^2^ cell to a 9-cm^2^ electrolyzer to validate the reliability and scalability of the carbon efficiency and EE (figs. S45 to S47). Notably, the FE_CO_ is still maintained at ~60% in a 9-cm^2^ cell electrolyzer without any electrolyte additives, which is considerably better than all the previous reports regardless of the separators, electrolytes, and cell testing conditions (*28*, *29*). To mimic industrial conditions, we implemented continuous electrochemical conversion of dilute CO_2_ under 20 vol % simulated flue gas conditions using both BPM and AEM configurations, as shown in Fig. 6 (D and E). When operated with a BPM, the system achieved a high single-pass CO_2_-to-CO conversion efficiency of ~42% from a dilute CO_2_ source, while maintaining an FE_CO_ of ~37% and an EE of ~15%. In comparison, the AEM configuration exhibited superior electrochemical performance, enabling CO production at ~42% FE_CO_ under a relatively low whole-cell voltage of ~2.3 V. Both configurations demonstrated stable performance over a 150-hour continuous electrolysis period, with no noticeable fluctuations in the evaluated parameters. As shown in Fig. 6 (F to H) and note S11, our energy analysis reveals that, compared to the conventional gas-phase CO_2_ reduction configuration (66.86 GJ per ton), our integrated approach substantially lowers the energy requirement to 46.79 GJ per ton of CO for the AEM-based electrolyzer and 64.32 GJ per ton for the BPM-based configuration (*30*). With projected system optimizations, these values could be further decreased to 36.17 and 45.79 GJ per ton of CO, respectively, marking a substantial advancement in energy-efficient CO_2_ utilization. The efficiency gains of ICCE are rooted in the excellent carbamate electrolysis pathways enabled by our screened PZ system and optimized Ni-based catalyst. This superior performance highlights the PZ-based system as a robust, reliable, and operationally simplified strategy for direct CO_2_ utilization (table S20), paving the way for scalable and energy-efficient carbon capture and conversion technologies.

**Table 2.** Comparative summary of ICCE.

Absorbent (Abs)	PZ (this work)			K-GLY (*28*)	K_2_CO_3_ + GLY (*29*)
	Evaluation of CO_2_ capture properties				
Category	Amine			Amino acid salt	Carbonate salt
Capture capacity (mol CO_2_ mol^−1^ absorbent)	1.02			0.7	~0.5
Initial capture rate (mol CO_2_ mol^−1^ absorbent min^−1^)	0.0458 (*59*)			0.0393 (*59*)	~0.0015 (*29*)
	Evaluation of ICEE				
Catalysts	Ni nanoparticles			Ni single atom	Ag nanoparticles
Membrane	PEM	AEM	BPM	PEM	BPM
Operating current density (mA cm^−2^)	50	150	150	50	100
Cell voltage (V)	2.62 ± 0.03	2.36 ± 0.04	3.21 ± 0.03	2.74	3.5
FE_CO_, CO_2_ sat. (%)	49.7	60.1	42.1	64	56
EE, CO_2_ sat. (%)	25.1	35.1	17.5	31	21.3
FE_CO_, 10–20 vol % CO_2_ (%) *	29.3–44.9	52.2–53.9	33.4–39.6	51†	33‡
EE, 10–20 vol % CO_2_ (%)	14.8–22.7	29.4–30.4	13.9–16.4	19†	12.5‡
Carbon crossover rate (mmol A^−1^ s^−1^)	0	0.0051–0.007	0	0	0
CO_2_-to-CO conversion (%)	38.1	23.2	72.2	9	50
Operation time (hour)	48	210	48	35	40

* Ten to 20 vol % CO_2_ refers to the CO_2_ volume fraction in the N_2_-balanced simulated flue gas used to saturate the absorbent solution.

† This value was obtained under 15 vol % CO_2_.

‡ This value was obtained under 20 vol % CO_2_.

## DISCUSSION

In this work, we systematically investigated six amine candidates with tailored structural features for their suitability in an integrated CO_2_ capture and electrochemical conversion scheme. Through this tandem evaluation framework, PZ emerged as the optimal absorbent. Its distinctive cyclic diamine structure and moderate carbamate formation energy collectively contribute to its outstanding performance not only in CO_2_ capture step but also in electrochemical conversion process. Both experimental data and theoretical calculations confirmed the active participation of PZ-carbamate species (accounting for ~20 to 60%) in the electrochemical reduction pathway, resulting in high Faradaic and carbon efficiencies for CO production. Using an AEM-based zero-gap MEA electrolyzer (9 cm^2^), the integrated system achieved robust operational stability over 150 hours with FE_CO_ ranging from 30 to 40% and an overall EE of ~20%. These metrics compare favorably with, and in many cases exceed, previously reported systems. Our findings highlight the importance of co-optimizing both absorbents and catalytic materials in the emerging field of ICCE. The tandem approach adopted here not only enhances system-level performance but also offers valuable insights for designing future sustainable carbon capture and utilization technologies.

## MATERIALS AND METHODS

### Materials

PZ, META, MP, EDA, PLA, PD, nickel(II) nitrate [Ni(NO_3_)_2_·6H_2_O], nickel(II) phthalocyanine (NiPc), nickel(II) oxide (NiO), ferric nitrate nonahydrate [Fe(NO_3_)_3_·9H_2_O], hydrochloric acid (HCl), nitric acid (HNO_3_), potassium chloride (KCl), potassium bicarbonate (KHCO_3_), 2MI, 2-aminoterephthalic acid (BDC), trimesic acid (BTC), *N*,*N*-dimethylformamide (DMF), ethanol, propan-2-ol (iPA), acetone, iridium(IV) oxide (IrO_2_), and iridium(III) chloride hydrate (IrCl_3_·3H_2_O) were brought from Sigma-Aldrich. Toray carbon paper TGP-H-060, TGP-H-090 with 5% PTFE, TGP-H-090 with 30% PTFE, Teflon DISP 30, titanium fiber felt (0.2- to 0.3-mm thickness), Ni foam (80 to 120 PPI, 1-mm thickness), cation exchange membrane (Nafion 117), AEM (Fumasep FAA-3-50), and BPM (Fumasep FBM-PK) were brought from the fuel store. High-purity argon (Ar), nitrogen (N_2_, 99.9%), and carbon dioxide (CO_2_, 99.9%) gases were purchased from BOC Gas. All chemicals were used directly without further purification. Furthermore, all water used for synthesis and analysis was purified by the Millipore purification system to a high purity of >18.2 megohm·cm.

### Quantitative evaluation of amine CO_2_ capture behavior

The viscosity (η) of the amine solutions was measured using the SV-1A vibrational viscometer (A&D Company Limited), which operates based on the tuning-fork vibration method with a natural frequency of 30 Hz. The measurable viscosity range of the instrument is 0.3 to 1000 mPa·s. All measurements were conducted at 25°C after thermal equilibration of the samples. The pH values were measured using an Aprea LabSen 211 pH electrode (3 M KCl), and the conductivity (σ) was determined using an Aprea DJS-0.1-S low-range conductivity electrode.

The CO_2_ loading capacity and associated ion species were determined by ^1^H and ^13^C NMR spectroscopy using Bruker 300 MHz NMR spectrometer, operating at resonance frequencies of 300.1 MHz for ^1^H and 100.6 MHz for ^13^C. In experiments, we acquired all ^13^C NMR spectra using a relaxation delay of 70 s and used 128 scans to ensure quantitative reliability for carbon-based species (carbamate and HCO_3_^−^/CO_3_^2−^ ions) (*31*). Meanwhile, to have a better quantity result for CO_2_ loadings, an external reference capillary containing 13.0% trioxane dissolved in D_2_O was put into the NMR tubes to improve the signal lock during acquisition of the spectra and for chemical shift calibration (*31*–*33*). All the NMR tests were carried out at 25°C. To prepare amine-captured CO_2_ solutions under gradient loading conditions, two types of fresh amine solutions (90 vol % H_2_O and 10 vol % D_2_O) with identical mole concentration were prepared in reagent bottles. For the first solution A, CO_2_ was bubbled for a sufficient length of time to ensure saturation of CO_2_ loading. The second sample B should not be exposed to any gas. Solutions A and B were mixed in varying volumes (1000, 900, and 800 μl, etc. of solution A with 0, 100, and 200 μl of solution B, etc., respectively) in 2-ml centrifuge tubes to achieve a uniform mixture. Then, 600 μl of the mixture was transferred into an NMR tube prefilled with a capillary containing ~13 wt % trioxane solution sealed inside ^13^C NMR analysis. The CO_2_ loading capacity (α) can be calculated via Eq. 5

$$\alpha =\frac{A_{\text{carbamate}}+A_{\text{bicarbonate}/\text{carbonate}}}{\frac{1}{n_{C}}\sum_{j=1}^{n_{C}}A_{C,j}}$$ (5)

where *A*_carbamate_ and *A*_bicarbonate/carbonate_ represent the integrated ^13^C NMR peak areas corresponding to CO_2_ captured as carbamate and bicarbonate/carbonate species, respectively, in the high chemical shift region. A_C,*j*_ denotes the integrated area of the ^13^C NMR signal for the *j*-th carbon in the amine molecule (amine backbone carbons only; solvent and standards excluded), and *n*_C_ is the total number of carbon atoms in the amine.

We integrated a bubble column reactor into the setup shown in fig. S9 to evaluate the kinetic behavior of CO_2_ uptake. In each experiment, 50 ml of freshly prepared amine solution (without preloaded CO_2_) was introduced into a 100-ml three-neck flask, which was immersed in a water bath to minimize temperature fluctuations during absorption. CO_2_ was continuously supplied at a flow rate of 100 ml min^−1^ using a Digital Mass Flow Controller (DMFC). Throughout the experiment, pH and temperature were recorded in real time at 1-s intervals using a LabSen 211 pH electrode (Aprea) filled with 3 M KCl and a calibrated thermometer immersed in the solution. This setup enabled continuous monitoring of acid-base evolution during CO_2_ absorption and provided quantitative insight into the uptake kinetics of each amine under well-controlled conditions. Accordingly, at discrete reaction times *t_n_* (time step n, in s) during the capture process, the hydrogen-ion concentration [H^+^]_n_ is given by Eq. 6. To quantify the capture rate, the rate of change of hydrogen-ion concentration (Eq. 7) is evaluated by the discrete-time approximation, where *t_n_* and *t*_*n* + 1_ are consecutive sampling time in nanoseconds and [H^+^]_n_ and [H^+^]_n + 1_ are concentrations. The quantification of hydroxide follows the same principle, using Eq. 8 (at 25°C, *Kw* = 10^−14^)

$${\left[H^{+}\right]}_{n}=10^{-pH_{n}}$$ (6)

$${\left.\frac{d\left[H^{+}\right]}{\mathrm{dt}}\right|}_{n}\approx \frac{{\left[H^{+}\right]}_{n+1}-{\left[H^{+}\right]}_{n}}{t_{n+1}-t_{n}}$$ (7)

$$\left[OH^{-}\right]=\frac{K_{w}}{\left[H^{+}\right]}$$ (8)

Vapor-liquid equilibria (VLE) and liquid-phase ionic speciation of the META/PZ-CO_2_ system were simulated using Aspen Plus (v14.5). Nonideal phase behavior was described using the nonrandom two-liquid (NRTL) activity-coefficient model, while electrolyte effects and chemical speciation were accounted for using the electrolyte NRTL (eNRTL, ELECNRTL in Aspen Plus) framework (*34*–*36*). Simulations were conducted at 20°C and 1 atm with an amine concentration of 1.5 M.

For the in situ ATR-FTIR spectroscopy of a 1.5 M PZ solution during CO_2_ bubbling, in situ ATR-FTIR measurements were performed using a Thermo Fisher Scientific Nicolet iS50 spectrometer equipped with a liquid nitrogen–cooled HgCdTe detector and a VeeMAX III ATR accessory (Pike Technologies). In situ ATR-FTIR spectroscopy was performed to investigate a 1.5 M PZ aqueous solution during CO_2_ bubbling. A freshly prepared 1.5 M PZ solution was first transferred into the ATR-FTIR reactor, and CO_2_ was continuously introduced at a flow rate of 30 SCCM. Infrared spectra were collected in the range of 800 to 3000 cm^−1^ under in situ conditions.

### Catalyst synthesis

For the synthesis of Ni-2MI precursor (Ni-2MI-Pre) (*18*), the synthesis of Ni-2MI is by calcination of the Ni-2MI-Pre in the tube furnace. For the precursor Ni-2MI-Pre synthesis, Ni(NO_3_)_2_·6H_2_O (2 mmol) was dissolved in 40 ml of methanol to form solution A. 2-MI (40 mmol) was dissolved in 40 ml of methanol and ultrasonicated for 1 hour to achieve the clear solution to form solution B. Solution B was added dropwise into solution A under vigorous stirring for an hour. The mixture solution was then transferred into a 100-ml Teflon autoclave and heated to 140°C for 12 hours. The orange precipitation after the hydrothermal was centrifuged, washed with methanol for three times, and fried under vacuum overnight.

For the synthesis of Ni-BDC-Pre (*37*), the synthesis of Ni-BDC is by calcination of the Ni-BDC-Pre precursor in the tube furnace. For the precursor Ni-BDC synthesis, Ni(NO_3_)_2_·6H_2_O (6.6 mmol) was dissolved in a mixed solvent containing ethanol (12.5 ml), DMF (12.5 ml), and H_2_O (12.5 ml) to form solution A. BDC-NH_2_ (7.5 mmol) was also dissolved in the mixed solvent mentioned above separately to form solution B. Then, solution A was added dropwise to solution B under vigorous stirring for 30 min. The mixture solution was then transferred into a 100-ml Teflon autoclave and heated to 100°C for 24 hours. The light green precipitation after the hydrothermal was centrifuged, washed with ethanol for three times, and fried under vacuum overnight.

For the synthesis of Ni-BTC-Pre (*18*), the synthesis of Ni-BTC is by calcination of the Ni-BTC-Pre precursor in the tube furnace. For the precursor Ni-BDC synthesis, Ni(NO_3_)_2_·6H_2_O (4 mmol) and BTC (4 mmol) were dissolved in a mixed solvent containing ethanol (25 ml), DMF (25 ml), and H_2_O (25 ml). Then, the mixture solution was vigorously stirred for 1 hour. The mixture solution was then transferred into a 100-ml Teflon autoclave and heated to 150°C for 10 hours. The light green precipitation after the hydrothermal was centrifuged, washed with ethanol for three times, and fried under vacuum overnight.

For the synthesis of ZIF-8 (*18*), the synthesis of nitrogen-doped carbon (NC) is by calcination of the ZIF-8 precursor in the tube furnace. For the precursor ZIF synthesis, Zn(NO_3_)_2_·6H_2_O (4 mmol) was dissolved in 100 ml of methanol to form solution A. 2-MI (16 mmol) was dissolved in 100 ml of methanol and ultrasonicated for 1 hour to achieve the clear solution to form solution B. Solution B was added dropwise into the solution A without stirring. After the addition, the mixture was sealed and transferred to a 60°C oven for 24 hours to complete the self-assembly process. The white precipitation after the hydrothermal was centrifuged, washed with methanol for three times, and fried under vacuum overnight.

For the synthesis of Ni-2MI, Ni-BDC, Ni-BTC, and NC, the Ni-2MI, Ni-BDC, and Ni-BTC were obtained by calcination of their corresponding precursors Ni-2MI-Pre, Ni-BDC-Pre, and Ni-BTC-Pre in a tube furnace in an Ar atmosphere at 800°C for 3 hours at a heating rate of 2°C min^−1^. The NC was obtained by calcination of ZIF-8 in a tube furnace in an Ar atmosphere at 900°C for an hour at a heating rate of 5°C min^−1^. Each corresponding catalyst was obtained after cooling down to room temperature.

For the acid wash of Ni-2MI, 100 mg of Ni-2MI obtained from previous steps was leached at in 50 ml of 1 M HCl for 6 hours and 50 ml of 1 M HNO_3_ for another 6 hours. Afterward, the sample was rinsed three times with Milli-Q water and subsequently reheated at 800°C in an Ar atmosphere for 1 hour to restore carbon crystallinity. This sample is referred to as Ni-2MI-A.

The IrO_2_/Ti felt (*38*) was prepared using a slightly modified thermal decomposition method. A 5 cm–by–5 cm Ti fiber felt was first ultrasonically cleaned in water and iPA for 30 min each, followed by drying. The dried felt was then immersed in 6 M HCl at 80°C for 45 min and thoroughly rinsed with deionized water. Afterward, the Ti felt was soaked in 20 ml of an iPA solution containing 15 mmol liter^−1^ IrCl_3_·*x*H_2_O and 2 M HCl. The felt was removed, dried at 60°C, and subsequently heated to 350°C at a rate of 10°C min^−1^, and then calcined for 10 min. This coating-calcination cycle was repeated approximately eight times until the IrO_2_ loading reached ~2 mg cm^−2^. In the final step, the sample was heated at a slower rate of 5°C min^−1^ to 400°C and calcined for 2 hours.

For the NiFe Layered Double Hydroxides (LDH) (*39*), Ni foam was ultrasonically cleaned in 2 M HCl for 20 min to remove surface oxides, followed by rinsing with acetone and deionized water, and then dried in air. Electrodeposition was performed in a three-electrode electrochemical cell containing an aqueous solution of 3 mM Ni(NO_3_)_2_·6H_2_O and 3 mM Fe(NO_3_)_3_·9H_2_O. A platinum wire and an Ag/AgCl electrode (3 M KCl) were used as the counter and reference electrodes, respectively. The deposition was carried out at −1.0 V (versus Ag/AgCl) for 300 s. After electrodeposition, the sample was carefully removed, rinsed with deionized water, and dried in air before further use.

### Catalyst characterization

A powder x-ray diffraction (XRD) pattern was examined on a Bruker Axs D4 Endeavor Wide-Angle XRD instrument. The sample morphologies were examined by TEM using a JEOL JEM-2100F operated at an acceleration voltage of 200 kV and further analyzed using a double-corrected Titan3 80–300 field-emission gun TEM. The analysis of x-ray photoelectron spectroscopy (XPS) was performed using the Thermo K-Alpha XPS system, which used Al Kα radiation within a chamber maintained at a pressure of 3 × 10^−10^ mbar. All XPS data were correlated with C 1s peak at 284.8 eV as a standard reference. The quantitative analysis of the metal composition in catalysts was performed using inductively coupled plasma optical emission spectrometry. The samples were initially digested with 5 ml of concentrated nitric acid (HNO_3_) and then subjected to microwave-assisted digestion using the Multiwave 7000 system. Following digestion, the solutions were transferred to 100-ml volumetric flasks and diluted to volume with Milli-Q water.

All XAFS data at Ni *K*-edge were collected at the MEX1 beamline in Australian Synchrotron (ANSTO), operated at 200 mA and 3 GeV. The scan range was kept in the energy range of 8315 and 8400 eV for Ni-edge. The Ni *K*-edge data were analyzed using the ATHENA module within the IFEFFIT software suite (*40*). The post-edge background was subtracted from the total absorption, followed by normalization against the edge jump step to obtain the EXAFS spectra. The χ(*k*) data in *k*-space were transformed into real (R) space through Fourier transformation using Hanning windows.

### Electrochemical measurements

In H-cell setup, 1 mg of catalyst was mixed with 330 μl of Milli-Q water, 620 μl of iPA, and 50 μl of 5 wt % Nafion solution. The mixture was then sonicated for at least 3 hours within an ice-water mixture. Subsequently, the homogeneous catalyst slurry was spray-coated onto either TGP-H-060, TGP-H-090 with 5% PTFE, or TGP-H-090 with 30% PTFE carbon paper, ensuring precise coverage of the designated area, as outlined in notes S3 and S4. Electrochemical measurements were performed in an H-cell using a three-electrode configuration, consisting of a working electrode, an Ag/AgCl (3 M KCl) reference electrode, and a platinum wire counter electrode. All potentials measured versus the reference electrode were converted to the RHE scale using Eq. 9

E(RHE)=E(versus Ag/AgCl)+0.205V+0.0592×pH (9)

Each compartment of the H-type cell is filled with 50 ml of solution—the anolyte consists of 1 M KOH, while the catholyte contains 50 ml of the specified amine solution. The anolyte and catholyte are separated by Nafion 117 membrane. Electrolysis in the H-cell was conducted using Biologic VSP-300. In the electrocatalytic reduction of gaseous CO_2_, both the cathode and anode electrolytes are 0.5 M KHCO_3_. In the electrocatalytic reduction of amine-CO_2_ under 1 atm CO_2_, the anolyte consists of 1 M KOH, while the catholyte contains the 1.5 M required amine solution. The flow rate of CO_2_ gas in letting the H-cell gas chamber was precisely controlled between 10 and 20 SCCM using a Horiba STEC s48-32 digital gas flow controller (DGFC). Before the reaction starts, a continuous flow of CO_2_ at 20 SCCM is introduced into the reactor for at least 30 min to ensure that the electrolyte is fully saturated with CO_2_. Linear sweep voltammetry was then performed from 0 to −1.3 V versus RHE at 5 mV s^−1^, and EIS was carried out using a 5-mV ac amplitude across 100 Hz to 100 kHz, either at open-circuit or onset potentials. Electrolysis was conducted under sealed conditions using chronoamperometry or chronopotentiometry within −0.5 to −1.2 V versus Ag/AgCl (or −9 to −81 mA). Each run lasted 10 min, after which 1 ml of gas from the reactor headspace was collected using a 2-ml Hamilton GASTIGHT syringe (PTFE luer-lock) for gas chromatography (GC; PerkinElmer Clarus 690) analysis. Between tests, the solution was resaturated with CO_2_ (10 SCCM) to recover 100% loading.

The FE_CO_ and H_2_ (FE_H2_) were calculated using Eq. 10

$$\mathrm{FE}=\frac{\mathrm{zF}}{Q}\cdot \frac{yV_{t}p}{R\times T}$$ (10)

where *z* is the number of electrons required to produce one molecule of gas (two for CO and H_2_ and eight for CH_4_); *F* (96485 C mol^−1^) is the Faraday constant; *Q* (coulomb) is the total charge passed, obtained from the electrochemical workstation; *y* (%) is the volume fraction of the target product quantified by GC; *V*_t_ (8.0 × 10^−5^ m^3^) is the total gas volume inside the reactor chamber; *p* (1.013 × 10^5^ Pa) is the atmospheric pressure; *R* (8.314 J mol^−1^ K^−1^) is the gas constant; and *T* (298 K) is the temperature.

In situ ATR-FTIR of electrochemical reductions in PZ-CO_2_ solution were performed using the same equipment as that used for CO_2_ capture experiments. Catalyst inks were prepared and spray-coated onto a Ge prism (incident angle of 60°), which was positioned at the bottom of the electrochemical cell and served as the working electrode. Electrochemical reduction tests of Ni-2MI and the metal-free catalyst NC were carried out in a 1.5 M PZ aqueous solution under in situ ATR-FTIR monitoring. The electrolyte was prepared by first saturating the PZ solution with CO_2_, followed by bubbling N_2_ for 1 hour to remove physically dissolved CO_2_, resulting in a final solution pH of 7.9. The 1.5 M PZ-captured CO_2_ solution was then subjected to electrochemical reduction under applied potentials ranging from 0 to −2.5 V versus RHE, during which time-resolved ATR-FTIR spectra were simultaneously collected to monitor interfacial species evolution.

Scale-up experiments were conducted using zero-gap electrolyzer setups with active areas of 1 and 9 cm^2^. During operation, the catholyte and anolyte were stored in separate reagent bottles and circulated continuously using two peristaltic pumps (Masterflex L/S, model no. 07528-10, equipped with pump head model 77200-60). Platinum-cured silicone tubing was used for fluid circulation. For the catholyte loop, L/S 25 tubing (inner diameter = 6.4 mm) was used, while the anolyte was circulated using L/S 13 tubing (inner diameter = 0.8 mm). The cathode consisted of carbon paper (TGP-H-090 with 5% PTFE) loaded with 4 mg cm^−2^ of Ni-2MI catalyst. The anode was either IrO_2_/Ti felt or NiFe LDH. The cathode and anode compartments were separated using either a PEM, an AEM, or a BPM, depending on the experimental condition. The catholyte comprised a fixed volume of 3 M PZ solution saturated with CO_2_, while the anolyte was either 2 M KOH or 0.05 M H_2_SO_4_, selected to match the ionic environment introduced by the membrane. The detailed experimental configuration, including component arrangements and flow paths, is provided in note S10 and table S19. Simulated flue gas was prepared by mixing defined flow rates of high-purity CO_2_ and N_2_ using a DGFC. The desired composition was achieved by adjusting the individual flow rates to mimic typical flue gas CO_2_ concentrations. The volume of gas generated during electrolysis was measured using the DGFC with ADA15 digital-to-analog converter. All measured gas volumes were corrected to standard conditions using experimentally determined volume correction factors: N_2_ (1.00), CO_2_ (0.74), CH_4_ (0.785), H_2_ (1.01), and CO (1.00). Potentiostatic electrolysis was performed using a CHI760e electrochemical workstation (CH Instruments). When the current exceeded 250 mA, a CHI680 AMP booster was connected in series to maintain stable operation.

### Computational methods and theoretical calculations

The geometries of the PZ-CO_2_ ionic species were optimized, followed by vibrational frequency calculations using Gaussian 16 (*41*), to generate the corresponding simulated infrared spectra. All calculations were carried out at the M06-2X/6-311++G(d,p) level of theory. Solvent effects were included using the solvation model density (SMD) implicit solvent model (*42*).

DFT calculations were performed using the MedeA VASP software with the projected augmented wave method (*43*, *44*). The exchange and correlation effects were treated using the generalized gradient approximation with the Perdew-Burke-Ernzerhof functional (*45*). The plane-wave cutoff was set to be 500 eV, and the force criterion for the convergence was 0.02 eV. A Γ-centered 3 by 3 by 1 *k*-point mesh was used for sampling of the Brillouin zone. The exploration of the potential energy surface for the Ni*_n_* clusters (*n* = 3, 4, …, 20) was performed using the stochastic surface walking method in conjunction with a neural network (NN) potential (*46*, *47*), as implemented in the large-scale atomic simulation with NN potential (LASP) code (*48*).

The second-order difference energy (Δ_2_*E*), a stability descriptor reflecting the relative stability of a cluster compared to its neighboring sizes (*49*–*51*), can be expressed as Eq. 11

$$\Delta_{2}E=E_{Ni_{n+1}}-E_{Ni_{n-1}}-2\cdot E_{Ni_{n}}$$ (11)

where *n* is the number of Ni atoms in the cluster. $E_{Ni_{n}}$, $E_{Ni_{n+1}}$, and $E_{Ni_{n-1}}$ are the total energies of gas-phase Ni*_n_*, Ni_*n* + 1_, and Ni_*n*-1_ clusters, respectively. A positive Δ_2_*E* indicates that the Ni*_n_* cluster is energetically more favorable than its immediate larger (Ni_*n* + 1_) and smaller (Ni_*n*−1_) neighbors. The adsorption energies (*E*_ads_) of various Ni*_n_* clusters on graphene substrate were calculated according to Eq. 12

$$E_{\text{ads}}=E_{Ni_{n}+\text{sub}}-E_{\text{sub}}-E_{Ni_{n}}$$ (12)

where the $E_{Ni_{n}/\text{sub}}$, and $E_{\text{sub}}$ are the total energies of graphene substrate decorated with Ni*_n_* cluster and graphene substrate, respectively. To gain deeper insight into the adsorption process, *E*_ads_ was further decomposed into the electronic interaction energy (*E*_int_) and deformation energy (*E*_def_), as shown in Eq. 13

$$E_{\text{ads}}=E_{\text{int}}+E_{\text{def}}$$ (13)

*E*_def_ accounts for the energy penalty associated with the geometric distortion of the adsorbate and substrate upon interaction. It can be defined as Eq. 14

$$E_{\text{def}}=E_{{\text{Ni}}_{n}}^{\text{sp}}-E_{{\text{Ni}}_{n}}+E_{\text{sub}}^{\text{sp}}-E_{\text{sub}}$$ (14)

where $E_{{\text{Ni}}_{n}}^{\text{sp}}$ and $E_{\text{sub}}^{\text{sp}}$ are the single-point energies of the Ni*_n_* clusters and graphene substrate, respectively, calculated at the deformed geometries extracted from the optimized Ni*_n_*-2MI catalysts.

The binding energy of Ni_6_ cluster was calculated according to Eq. 15

$$E_{b}=\left(E_{Ni_{n}/\text{sub}}-E_{\text{sub}}-n\cdot E_{Ni_{n}}\right)/n$$ (15)

Structural models presented in the main text were visualized using VESTA (*52*). The Gibbs free energy accounts for zero-point energy (ZPE), thermal contributions, and entropy, all of which were derived from partition functions (*53*). For adsorbed surface species, the ZPE correction was applied as follows (Eq. 16)

$$E_{\text{ZPE}}=\sum_{i}\frac{h\nu_{i}}{2}$$ (16)

where *h* is Plank’s constant and *v_i_* is the vibrational frequency *i*, which is calculated based on the harmonic oscillator approximation.

The standard molar vibrational thermal energy was determined using the following equation (Eq. 17)

$$U_{\text{vib}}=\mathrm{RT}\sum_{i}\frac{h\nu_{i}/k_{B}T}{e^{h\nu_{i}/k_{B}T}-1}$$ (17)

where *R* is the gas constant and *k*_B_ is Boltzmann’s constant.

The standard molar vibrational entropy was calculated using the following expression (Eq. 18)

$$S_{\text{vib}}=R\sum_{i}\left[\frac{h\nu_{i}/k_{B}T}{e^{h\nu_{i}/k_{B}T}-1}-\text{ln}\left(1-e^{h\nu_{i}/k_{B}T}\right)\right]$$ (18)

Therefore, the standard molar Gibbs free energy was obtained by Eq. 19

$$G=E_{\text{total}}+E_{\text{ZPE}}+U–\mathrm{TS}$$ (19)

where *E*_total_ refers to the total energy obtained from the DFT calculations.

The formation energies of amine carbamates and bond length were calculated using Gaussian 16 (*41*) with the B3LYP functional (*54*), using the def2-TZVP basis set (*55*, *56*). Grimme’s D3 dispersion correction with Becke-Johnson damping was applied to account for van der Waals interactions (*57*, *58*). Solvent effects were modeled using the SMD variant of the IEFPCM approach (*42*).

## Supplementary Materials

### The PDF file includes:

Notes S1 to S11 Figs. S1 to S47 Tables S1 to S20 Legend for data S1 References

## References

[1] X. Lan, P. Tans, K. W. Thoning, Trends in globally-averaged CO_2_ determined from NOAA Global Monitoring Laboratory measurements (2025); 10.15138/9N0H-ZH07.

[2] Y. Zhong, Z. Zheng, D. Hao, H. Jin, X. Zheng, Y. Li, H. Yu, B. Jia, T. Ma, P. Li, Electrochemically integrated carbon capture and utilization. ACS Mater. Lett. 7, 3952–3973 (2025).

[3] L. Chen, G. Msigwa, M. Yang, A. I. Osman, S. Fawzy, D. W. Rooney, P.-S. Yap, Strategies to achieve a carbon neutral society: A review. Environ. Chem. Lett. 20, 2277–2310 (2022). 35431715 10.1007/s10311-022-01435-8 PMC8992416

[4] F. Meng, Y. Meng, T. Ju, S. Han, L. Lin, J. Jiang, Research progress of aqueous amine solution for CO_2_ capture: A review. Renew. Sustain. Energy Rev. 168, 112902 (2022).

[5] G. Lee, Y. C. Li, J.-Y. Kim, T. Peng, D.-H. Nam, A. Sedighian Rasouli, F. Li, M. Luo, A. H. Ip, Y.-C. Joo, E. H. Sargent, Electrochemical upgrade of CO_2_ from amine capture solution. Nat. Energy 6, 46–53 (2021).

[6] L. Chen, F. Li, Y. Zhang, C. L. Bentley, M. Horne, A. M. Bond, J. Zhang, Electrochemical reduction of carbon dioxide in a monoethanolamine capture medium. ChemSusChem 10, 4109–4118 (2017). 28799204 10.1002/cssc.201701075

[7] X. Y. D. Soo, J. J. C. Lee, W.-Y. Wu, L. Tao, C. Wang, Q. Zhu, J. Bu, Advancements in CO_2_ capture by absorption and adsorption: A comprehensive review. J. CO_2_ Util. 81, 102727 (2024).

[8] K. Shen, D. Cheng, E. Reyes-Lopez, J. Jang, P. Sautet, C. G. Morales-Guio, On the origin of carbon sources in the electrochemical upgrade of CO_2_ from carbon capture solutions. Joule 7, 1260–1276 (2023).

[9] G. Leverick, E. M. Bernhardt, A. I. Ismail, J. H. Law, A. Arifutzzaman, M. K. Aroua, B. M. Gallant, Uncovering the active species in amine-mediated CO_2_ reduction to CO on Ag. ACS Catal. 13, 12322–12337 (2023).

[10] A. Parkin, I. D. H. Oswald, S. Parsons, Structures of piperazine, piperidine and morpholine. Acta Crystallogr. Sect. B Struct. Sci. 60, 219–227 (2004). 10.1107/S0108768104003672 15017096

[11] B. Yu, H. Yu, K. Li, Q. Yang, R. Zhang, L. Li, Z. Chen, Characterisation and kinetic study of carbon dioxide absorption by an aqueous diamine solution. Appl. Energy 208, 1308–1317 (2017).

[12] S. Wada, T. Kushida, H. Itagaki, T. Shibue, H. Kadowaki, J. Arakawa, Y. Furukawa, ^13^C NMR study on carbamate hydrolysis reactions in aqueous amine/CO_2_ solutions. Int. J. Greenh. Gas Con. 104, 103175 (2021).

[13] H. Jiang, I. Novak, Piperidine–CO_2_–H_2_O molecular complex. J. Mol. Struct. 645, 177–183 (2003).

[14] G. Rochelle, E. Chen, S. Freeman, D. Van Wagener, Q. Xu, A. Voice, Aqueous piperazine as the new standard for CO_2_ capture technology. Chem. Eng. J. 171, 725–733 (2011).

[15] Y. E. Kim, J. H. Choi, S. C. Nam, Y. I. Yoon, CO_2_ absorption characteristics in aqueous K_2_CO_3_/piperazine solution by NMR spectroscopy. Ind. Eng. Chem. Res. 50, 9306–9313 (2011).

[16] S. A. Freeman, J. Davis, G. T. Rochelle, Degradation of aqueous piperazine in carbon dioxide capture. Int. J. Greenh. Gas Con. 4, 756–761 (2010).

[17] G. Puxty, R. Rowland, A. Allport, Q. Yang, M. Bown, R. Burns, M. Maeder, M. Attalla, Carbon dioxide postcombustion capture: A novel screening study of the carbon dioxide absorption performance of 76 amines. Environ. Sci. Technol. 43, 6427–6433 (2009). 19746747 10.1021/es901376a

[18] M. Liang, Y. Liu, J. Zhang, F. Wang, Z. Miao, L. Diao, J. Mu, J. Zhou, S. Zhuo, Understanding the role of metal and N species in M@NC catalysts for electrochemical CO_2_ reduction reaction. Appl. Catal. Environ. 306, 121115 (2022).

[19] Z. Xing, L. Hu, D. S. Ripatti, X. Hu, X. Feng, Enhancing carbon dioxide gas-diffusion electrolysis by creating a hydrophobic catalyst microenvironment. Nat. Commun. 12, 136 (2021). 33420043 10.1038/s41467-020-20397-5 PMC7794506

[20] X. Sheng, W. Ge, H. Jiang, C. Li, Engineering Ni─N─C catalyst microenvironment enabling CO_2_ electroreduction with nearly 100% CO selectivity in acid. Adv. Mater. 34, e2201295 (2022). 35901104 10.1002/adma.202201295

[21] M. Mortazavi, K. Tajiri, Effect of the PTFE content in the gas diffusion layer on water transport in polymer electrolyte fuel cells (PEFCs). J. Power Sources 245, 236–244 (2014).

[22] W. Zhang, J. Ma, P. Wang, Z. Wang, F. Shi, H. Liu, Investigations on the interfacial capacitance and the diffusion boundary layer thickness of ion exchange membrane using electrochemical impedance spectroscopy. J. Membr. Sci. 502, 37–47 (2016).

[23] A. Zanone, D. T. Tavares, J. L. d. Paiva, An FTIR spectroscopic study and quantification of 2-amino-2-methyl-1-propanol, piperazine and absorbed carbon dioxide in concentrated aqueous solutions. Vib. Spectrosc. 99, 156–161 (2018).

[24] K. Robinson, A. McCluskey, M. I. Attalla, An FTIR spectroscopic study on the effect of molecular structural variations on the CO_2_ absorption characteristics of heterocyclic amines. ChemPhysChem 12, 1088–1099 (2011). 21472963 10.1002/cphc.201001056

[25] J. Thompson, H. Richburg, K. Liu, Thermal degradation pathways of aqueous diamine CO_2_ capture solvents. Energy Procedia 114, 2030–2038 (2017).

[26] T. Nguyen, M. Hilliard, G. Rochelle, Volatility of aqueous amines in CO_2_ capture. Energy Procedia 4, 1624–1630 (2011).

[27] L. Ge, H. Rabiee, M. Li, S. Subramanian, Y. Zheng, J. H. Lee, T. Burdyny, H. Wang, Electrochemical CO_2_ reduction in membrane-electrode assemblies. Chem 8, 663–692 (2022).

[28] Y. C. Xiao, S. S. Sun, Y. Zhao, R. K. Miao, M. Fan, G. Lee, Y. Chen, C. M. Gabardo, Y. Yu, C. Qiu, Z. Guo, X. Wang, P. Papangelakis, J. E. Huang, F. Li, C. P. O’Brien, J. Kim, K. Han, P. J. Corbett, J. Y. Howe, E. H. Sargent, D. Sinton, Reactive capture of CO_2_ via amino acid. Nat. Commun. 15, 7849 (2024). 39245666 10.1038/s41467-024-51908-3 PMC11381538

[29] Y. Kim, E. W. Lees, C. Donde, A. M. L. Jewlal, C. E. B. Waizenegger, B. M. W. de Hepcée, G. L. Simpson, A. Valji, C. P. Berlinguette, Integrated CO_2_ capture and conversion to form syngas. Joule 8, 3106–3125 (2024).

[30] H. Shin, K. U. Hansen, F. Jiao, Techno-economic assessment of low-temperature carbon dioxide electrolysis. Nat. Sustainability 4, 911–919 (2021).

[31] A. F. Ciftja, A. Hartono, H. F. Svendsen, ^13^C NMR as a method species determination in CO_2_ absorbent systems. Int. J. Greenh. Gas Con. 16, 224–232 (2013).

[32] M. Nitta, M. Hirose, T. Abe, Y. Furukawa, H. Sato, Y. Yamanaka, ^13^C-NMR spectroscopic study on chemical species in piperazine−amine−CO_2_−H_2_O system before and after heating. Energy Procedia 37, 869–876 (2013).

[33] M. Xiao, D. Cui, Q. Yang, Z. Liang, G. Puxty, H. Yu, L. Li, W. Conway, P. Feron, Role of mono- and diamines as kinetic promoters in mixed aqueous amine solution for CO_2_ capture. Chem. Eng. Sci. 229, 116009 (2021).

[34] H. Renon, J. M. Prausnitz, Local compositions in thermodynamic excess functions for liquid mixtures. AIChE J. 14, 135–144 (1968).

[35] P. Frailie, J. Plaza, D. Van Wagener, G. T. Rochelle, Modeling piperazine thermodynamics. Energy Procedia 4, 35–42 (2011).

[36] Y. Du, G. T. Rochelle, Thermodynamic modeling of aqueous piperazine/*N*-(2-aminoethyl) piperazine for CO_2_ capture. Energy Procedia 63, 997–1017 (2014).

[37] Q. Lu, C. Chen, Q. Di, W. Liu, X. Sun, Y. Tuo, Y. Zhou, Y. Pan, X. Feng, L. Li, D. Chen, J. Zhang, Dual role of pyridinic-N doping in carbon-coated Ni nanoparticles for highly efficient electrochemical CO_2_ reduction to CO over a wide potential range. ACS Catalysis 12, 1364–1374 (2022).

[38] W. Luc, J. Rosen, F. Jiao, An Ir-based anode for a practical CO_2_ electrolyzer. Catal. Today 288, 79–84 (2017).

[39] X. Lu, C. Zhao, Electrodeposition of hierarchically structured three-dimensional nickel–iron electrodes for efficient oxygen evolution at high current densities. Nat. Commun. 6, 6616 (2015). 25776015 10.1038/ncomms7616 PMC4382694

[40] B. Ravel, M. Newville, ATHENA and ARTEMIS: Interactive graphical data analysis using IFEFFIT. Phys. Scr. 2005, 1007 (2005). 10.1107/S0909049505012719 15968136

[41] M. J. Frisch, G. W. Trucks, H. B. Schlegel, G. E. Scuseria, M. A. Robb, J. R. Cheeseman, G. Scalmani, V. Barone, G. A. Petersson, H. Nakatsuji, X. Li, M. Caricato, A. V. Marenich, J. Bloino, B. G. Janesko, R. Gomperts, B. Mennucci, H. P. Hratchian, J. V. Ortiz, A. F. Izmaylov, J. L. Sonnenberg, Williams, F. Ding, F. Lipparini, F. Egidi, J. Goings, B. Peng, A. Petrone, T. Henderson, D. Ranasinghe, V. G. Zakrzewski, J. Gao, N. Rega, G. Zheng, W. Liang, M. Hada, M. Ehara, K. Toyota, R. Fukuda, J. Hasegawa, M. Ishida, T. Nakajima, Y. Honda, O. Kitao, H. Nakai, T. Vreven, K. Throssell, J. A. Montgomery Jr., J. E. Peralta, F. Ogliaro, M. J. Bearpark, J. J. Heyd, E. N. Brothers, K. N. Kudin, V. N. Staroverov, T. A. Keith, R. Kobayashi, J. Normand, K. Raghavachari, A. P. Rendell, J. C. Burant, S. S. Iyengar, J. Tomasi, M. Cossi, J. M. Millam, M. Klene, C. Adamo, R. Cammi, J. W. Ochterski, R. L. Martin, K. Morokuma, O. Farkas, J. B. Foresman, D. J. Fox, GaussView 5.0 (Gaussian, Inc., 2016).

[42] A. V. Marenich, C. J. Cramer, D. G. Truhlar, Universal solvation model based on solute electron density and on a continuum model of the solvent defined by the bulk dielectric constant and atomic surface tensions. J. Phys. Chem. B. 113, 6378–6396 (2009). 19366259 10.1021/jp810292n

[43] G. Kresse, J. Furthmüller, Efficient iterative schemes for ab initio total-energy calculations using a plane-wave basis set. Phys. Rev. E 54, 11169–11186 (1996). 10.1103/physrevb.54.11169 9984901

[44] P. E. Blöchl, Projector augmented-wave method. Phys. Rev. E 50, 17953–17979 (1994). 10.1103/physrevb.50.17953 9976227

[45] J. P. Perdew, K. Burke, M. Ernzerhof, Generalized gradient approximation made simple. Phys. Rev. Lett. 77, 3865–3868 (1996). 10062328 10.1103/PhysRevLett.77.3865

[46] C. Shang, Z.-P. Liu, Stochastic surface walking method for structure prediction and pathway searching. J. Chem. Theor. Comput. 9, 1838–1845 (2013). 10.1021/ct301010b 26587640

[47] X.-T. Xie, Z.-X. Yang, D. Chen, Y.-F. Shi, P.-L. Kang, S. Ma, Y.-F. Li, C. Shang, Z.-P. Liu, LASP to the future of atomic simulation: Intelligence and automation. Precis. Chem. 2, 612–627 (2024). 39734761 10.1021/prechem.4c00060 PMC11672538

[48] S.-D. Huang, C. Shang, P.-L. Kang, X.-J. Zhang, Z.-P. Liu, LASP: Fast global potential energy surface exploration. WIREs Comput. Mol. Sci. 9, e1415 (2019).

[49] V. G. Grigoryan, M. Springborg, A theoretical study of the structure of Ni clusters (NiN). Phys. Chem. Chem. Phys. 3, 5135–5139 (2001).

[50] A. Granja-DelRío, H. A. Abdulhussein, R. L. Johnston, DFT-based global optimization of sub-nanometer Ni–Pd clusters. J. Phys. Chem. C 123, 26583–26596 (2019).

[51] H. Zhu, X. Li, N. Shi, X. Ding, Z. Yu, W. Zhao, H. Ren, Y. Pan, Y. Liu, W. Guo, Density functional theory study of thiophene desulfurization and conversion of desulfurization products on the Ni(111) surface and Ni55 cluster: Implication for the mechanism of reactive adsorption desulfurization over Ni/ZnO catalysts. Cat. Sci. Technol. 11, 1615–1625 (2021).

[52] K. Momma, F. Izumi, VESTA: A three-dimensional visualization system for electronic and structural analysis. J. Appl. Cryst. 41, 653–658 (2008).

[53] Y. Mao, Z. Wang, H.-F. Wang, P. Hu, Understanding catalytic reactions over zeolites: A density functional theory study of selective catalytic reduction of NO*_x_* by NH_3_ over Cu-SAPO-34. ACS Catal. 6, 7882–7891 (2016).

[54] C. Lee, W. Yang, R. G. Parr, Development of the Colle-Salvetti correlation-energy formula into a functional of the electron density. Phys. Rev. E 37, 785–789 (1988). 10.1103/physrevb.37.785 9944570

[55] F. Weigend, R. Ahlrichs, Balanced basis sets of split valence, triple zeta valence and quadruple zeta valence quality for H to Rn: Design and assessment of accuracy. Phys. Chem. Chem. Phys. 7, 3297–3305 (2005). 16240044 10.1039/b508541a

[56] F. Weigend, Accurate Coulomb-fitting basis sets for H to Rn. Phys. Chem. Chem. Phys. 8, 1057–1065 (2006). 16633586 10.1039/b515623h

[57] S. Grimme, J. Antony, S. Ehrlich, H. Krieg, A consistent and accurate ab initio parametrization of density functional dispersion correction (DFT-D) for the 94 elements H-Pu. J. Chem. Phys. 132, 154104 (2010). 20423165 10.1063/1.3382344

[58] J. Klimeš, D. R. Bowler, A. Michaelides, Van der Waals density functionals applied to solids. Phys. Rev. E 83, 195131 (2011).

[59] H.-J. Song, S. Park, H. Kim, A. Gaur, J.-W. Park, S.-J. Lee, Carbon dioxide absorption characteristics of aqueous amino acid salt solutions. Int. J. Greenh. Gas Con. 11, 64–72 (2012).

[60] H. Karlsson, H. Svensson, Rate of absorption for CO_2_ absorption systems using a wetted wall column. Energy Procedia 114, 2009–2023 (2017).

[61] J. H. Kim, H. Jang, G. Bak, W. Choi, H. Yun, E. Lee, D. Kim, J. Kim, S. Y. Lee, Y. J. Hwang, The insensitive cation effect on a single atom Ni catalyst allows selective electrochemical conversion of captured CO_2_ in universal media. Energ. Environ. Sci. 15, 4301–4312 (2022).

[62] H. B. Yang, S.-F. Hung, S. Liu, K. Yuan, S. Miao, L. Zhang, X. Huang, H.-Y. Wang, W. Cai, R. Chen, J. Gao, X. Yang, W. Chen, Y. Huang, H. M. Chen, C. M. Li, T. Zhang, B. Liu, Atomically dispersed Ni(i) as the active site for electrochemical CO_2_ reduction. Nat. Energy 3, 140–147 (2018).

[63] E. N. Fuller, J. C. Giddings, A comparison of methods for predicting gaseous diffusion coefficients. J. Chromatogr. Sci. 3, 222–227 (1965).

[64] E. D. Snijder, M. J. M. Te Riele, G. F. Versteeg, W. P. M. Van Swaaij, Diffusion coefficients of several aqueous alkanolamine solutions. J. Chem. Eng. Data 38, 475–480 (1993).

[65] G. F. Versteeg, W. P. M. Van Swaaij, Solubility and diffusivity of acid gases (carbon dioxide, nitrous oxide) in aqueous alkanolamine solutions. J. Chem. Eng. Data 33, 29–34 (1988).

[66] L.-C. Chang, T.-I. Lin, M.-H. Li, Mutual diffusion coefficients of some aqueous alkanolamines solutions. J. Chem. Eng. Data 50, 77–84 (2005).

[67] C. G. Zoski, J. Leddy, A. J. Bard, L. R. Faulkner, H. S. White, *Electrochemical Methods: Fundamentals and Applications, 3e Student Solutions Manual* (Wiley, 2024).

[68] F. Chaves, Newman and balsara on electrochemical systems fourth edition. Electrochem. Soc. Interface 30, 11–12 (2021).

[69] G. Richner, G. Puxty, Assessing the chemical speciation during CO_2_ absorption by aqueous amines using in situ FTIR. Ind. Eng. Chem. Res. 51, 14317–14324 (2012).

[70] G. T. Rochelle, Amine scrubbing for CO_2_ capture. Science 325, 1652–1654 (2009). 19779188 10.1126/science.1176731

[71] A. Cousins, S. Huang, A. Cottrell, P. H. M. Feron, E. Chen, G. T. Rochelle, Pilot-scale parametric evaluation of concentrated piperazine for CO_2_ capture at an Australian coal-fired power station. Greenh. Gaes. Sci. Technol. 5, 7–16 (2015).

[72] M. Bui, C. S. Adjiman, A. Bardow, E. J. Anthony, A. Boston, S. Brown, P. S. Fennell, S. Fuss, A. Galindo, L. A. Hackett, J. P. Hallett, H. J. Herzog, G. Jackson, J. Kemper, S. Krevor, G. C. Maitland, M. Matuszewski, I. S. Metcalfe, C. Petit, G. Puxty, J. Reimer, D. M. Reiner, E. S. Rubin, S. A. Scott, N. Shah, B. Smit, J. P. M. Trusler, P. Webley, J. Wilcox, N. M. Dowell, Carbon capture and storage (CCS): The way forward. Energ. Environ. Sci. 11, 1062–1176 (2018).

[73] J. C. Bui, E. W. Lees, D. H. Marin, T. N. Stovall, L. Chen, A. Kusoglu, A. C. Nielander, T. F. Jaramillo, S. W. Boettcher, A. T. Bell, A. Z. Weber, Multi-scale physics of bipolar membranes in electrochemical processes. Nat. Chem. Eng. 1, 45–60 (2024).

[74] A. V. Rayer, K. Z. Sumon, L. Jaffari, A. Henni, Dissociation constants (pKa) of tertiary and cyclic amines: structural and temperature dependences. J. Chem. Eng. Data 59, 3805–3813 (2014).

[75] N. Bonanos, B. C. H. Steele, E. P. Butler, J. R. Macdonald, W. B. Johnson, W. L. Worrell, G. A. Niklasson, S. Malmgren, M. Strømme, S. K. Sundaram, M. C. H. McKubre, D. D. Macdonald, G. R. Engelhardt, E. Barsoukov, B. E. Conway, W. G. Pell, N. Wagner, C. M. Roland, R. S. Eisenberg, “Applications of Impedance Spectroscopy,” in *Impedance Spectroscopy* (John Wiley & Sons, Ltd, 2018), pp. 175–478.

[76] Y. Kim, M. Namdari, A. M. L. Jewlal, Y. Chen, D. J. D. Pimlott, M. Stolar, C. P. Berlinguette, Economic viability of integrated CO_2_ capture and conversion. ACS Energy Lett. 10, 403–409 (2025).

[77] H. M. Almajed, R. Kas, P. Brimley, A. M. Crow, A. Somoza-Tornos, B.-M. Hodge, T. E. Burdyny, W. A. Smith, Closing the loop: Unexamined performance trade-offs of integrating direct air capture with (Bi)carbonate electrolysis. ACS Energy Lett. 9, 2472–2483 (2024). 38751972 10.1021/acsenergylett.4c00807 PMC11091874

[78] B. Belsa, L. Xia, V. Golovanova, B. Polesso, A. Pinilla-Sánchez, L. San Martín, J. Ye, C.-T. Dinh, F. P. García de Arquer, Materials challenges on the path to gigatonne CO_2_ electrolysis. Nat. Rev. Mater. 9, 535–549 (2024).

[79] S. Ren, D. Joulié, D. Salvatore, K. Torbensen, M. Wang, M. Robert, C. P. Berlinguette, Molecular electrocatalysts can mediate fast, selective CO_2_ reduction in a flow cell. Science 365, 367–369 (2019). 31346062 10.1126/science.aax4608

[80] M. D. Porosoff, B. Yan, J. G. Chen, Catalytic reduction of CO_2_ by H_2_ for synthesis of CO, methanol and hydrocarbons: Challenges and opportunities. Energ. Environ. Sci. 9, 62–73 (2016).

