# Supplementary material for: Zwitterionic carbamate interfaces unlock efficient “liquid” CO2 upgrading
Source: Sci Adv. 2026 May 13;12(20):eaed8640. doi: 10.1126/sciadv.aed8640 (PMC13170645; doi:10.1126/sciadv.aed8640)
Supplement: Supplementary file 1 — Notes S1 to S11 Figs. S1 to S47 Tables S1 to S20 Legend for data S1 References [file sciadv.aed8640_sm.pdf]

Science Advances
AAAS

Supplementary Materials for

Zwitterionic carbamate interfaces unlock efficient “liquid” CO_2 upgrading

Yitong Li *et al.*

Corresponding author: Peng Li, peng.li2@rmit.edu.au; Chongchong Wu, chongchong.wu@ucalgary.ca; Tianyi Ma, tianyi.ma@rmit.edu.au

Sci. Adv. **12**, eaed8640 (2026)
DOI: 10.1126/sciadv.aed8640

The PDF file includes:

Notes S1 to S11
Figs. S1 to S47
Tables S1 to S20
Legend for data S1
References

Other Supplementary Material for this manuscript includes the following:

Data S1

Note S1: Amine-CO_2 reaction and composition determination.

Capture Mechanism: The reaction of **monoamines (MTEA, MP, PLA, and PD)**, with CO_2 proceeds through a stepwise zwitterionic mechanism (*33*). Initially, nucleophilic attack of the amine on CO_2 leads to the formation of an N,N-zwitterionic intermediate ($R{-}NH_2^+{-}CO_2^-$) (Eq. S1). This intermediate subsequently undergoes proton transfer with a second amine molecule to yield an amine carbamate ($R{-}NH{-}CO_2^-$) together with a protonated amine ($R{-}NH_3^+$) (Eq. S2). Upon prolonged CO_2 exposure, $R{-}NH{-}CO_2^-$ species undergo hydrolysis, during which protonation of the amine occurs, while the concomitantly generated hydroxide (OH^-) reacts with CO_2 to form bicarbonate (HCO_3^-) (Eq. S3).

$$R{-}NH_2 + CO_2 \leftrightarrow R{-}NH_2^+{-}CO_2^- \quad (S1)$$

$$R{-}NH_2^+{-}CO_2^- + R{-}NH_2 \leftrightarrow R{-}NH_3^+ + R{-}NH{-}CO_2^- \quad (S2)$$

$$R{-}NH{-}CO_2^- + 2H_2O + CO_2 \leftrightarrow R{-}NH_3^+ + 2HCO_3^- \quad (S3)$$

For **diamines (PZ and EDA)**, the presence of two amino groups enables additional reaction pathways beyond those observed for monoamines (*32*). At the initial stage of CO_2 capture, diamines follow a mechanism analogous to that of monoamines, involving proton transfer with a second amine molecule to yield a diamine carbamate ($H_2N{-}R_1{-}R_n{-}NH{-}COO^-$) (Eq. S4). In pathway 1, the presence of the unreacted amino group ($-NH_2$) enables proton acceptance during hydrolysis, such that $H_2N{-}R_1{-}R_n{-}NH{-}COO^-$ directly evolves into a stabilized protonated diamine carbamate ($H_3N^+{-}R_1{-}R_n{-}NH{-}COO^-$) (Eq. S5). In another dicarbamate pathway, $H_2N{-}R_1{-}R_n{-}NH{-}COO^-$ reacts with CO_2 and undergoes intermolecular proton transfer, resulting in the formation of an amine dicarbamate ($^-OOC{-}HN{-}R_1{-}R_n{-}NH{-}COO^-$) and a $H_3N^+{-}R_1{-}R_n{-}NH{-}COO^-$ (Eq. S6). $^-OOC{-}NH{-}R_1{-}R_n{-}NH{-}COO^-$ can undergo hydrolysis, during which protonation occurs, while the concomitantly generated hydroxide reacts with CO_2 to form HCO_3^-, yielding $H_3N^+{-}R_1{-}R_n{-}NH{-}COO^-$ (Eq. S7).

$$2H_2N{-}R_1{-}R_n{-}NH_2 + CO_2 \leftrightarrow H_2N{-}R_1{-}R_n{-}NH_3^+ + H_2N{-}R_1{-}R_n{-}NH{-}COO^- \quad (S4)$$

$$H_2N{-}R_1{-}R_n{-}NH{-}COO^- + CO_2 + H_2O \leftrightarrow H_3N^+{-}R_1{-}R_n{-}NH{-}COO^- + HCO_3^- \quad (S5)$$

$$2H_2N{-}R_1{-}R_n{-}NH{-}COO^- + CO_2 \leftrightarrow H_3N^+{-}R_1{-}R_n{-}NH{-}COO^- + {^-OOC}{-}HN{-}R_1{-}R_n{-}NH{-}COO^- \quad (S6)$$

$$^-OOC{-}HN{-}R_1{-}R_n{-}NH{-}COO^- + 2H_2O + CO_2 \leftrightarrow H_3N^+{-}R_1{-}R_n{-}NH{-}COO^- + 2HCO_3^- \quad (S7)$$

Compared to PD and PLA, META and MP exhibit relatively higher pK_b values and thus weaker basicity, which favors the formation of more stable amine carbamate species and suppresses their conversion to bicarbonate (Eq. S8−9). In contrast, PD and PLA possess stronger basicity, as reflected by their lower pK_b values and higher pK_a of the corresponding conjugate acids. Under CO_2-rich conditions, the protonated forms of PD and PLA are weaker acids than bicarbonate (p$K_a \approx 10.3$), allowing them to accept protons and thereby promote carbamate hydrolysis toward bicarbonate formation (Eq. S10−11). These trends are consistent with the NMR results.

$$3META + 2CO_2 + 2H_2O \leftrightarrow 2METAH^+ + META{-}COO^- + HCO_3^- \quad (S8)$$

$$3MP + 2CO_2 + 2H_2O \leftrightarrow 2MPH^+ + MP{-}COO^- + HCO_3^- \quad (S9)$$

$$PD + CO_2 + H_2O \leftrightarrow PDH^+ + HCO_3^- \quad (S10)$$

$$PLA + CO_2 + H_2O \leftrightarrow PLAH^+ + HCO_3^- \quad (S11)$$

The concentrations of ion species derived from ^{13}C NMR spectra at different CO_2 loadings for a 1.5 mol L^{-1} amine-captured CO_2 solutions are listed in the Table S3−6.

Distribution of CO_2 species in amine capture solutions: In amine–CO_2 capture solutions, the distribution of CO_2-derived species was quantified in Fig. S8. At full loading, only 29.8% and 20.1% of CO_2 existed as carbonate for META and MP, respectively, whereas substantially higher carbonate fractions were observed for the more basic amines PLA and PD (93.7% and 100%). This trend indicates that stronger basicity promotes pH reduction during CO_2 absorption, facilitating amine protonation and subsequent carbonate formation. In contrast, for diamines (EDA and PZ), CO_2 predominantly exists as protonated amine carbamate at high loadings. The presence of two amino groups enables enhanced proton stabilization, suppressing proton-induced displacement of CO_2 and thereby inhibiting conversion to carbonate. Consequently, unlike monoamine systems where carbonate becomes dominant at high loading, diamine systems favour carbamate-based CO_2 retention.

Kinetic evaluation of CO_2 absorption: To evaluate the dynamic performance of capture agents relevant to tandem electrochemical conversion, the CO_2 absorption kinetics were measured using pH–time profiles in a bubble column setup (Fig. S9) (*11, 60*). While the equilibrium CO_2 capacities of the six amines were discussed previously, the reaction rate is equally crucial, as it determines whether an amine can rapidly adsorb CO_2 before and after the electrochemical reduction step, thereby sustaining multiple conversion cycles. CO_2 capture by primary and secondary amines generally proceeds in two consecutive stages. In the initial stage, CO_2 reacts directly with the amine via nucleophilic attack on the carbon atom, forming a stable C–N bond and producing a carbamate intermediate. Subsequently, the carbamate species undergoes hydrolysis, during which hydroxide ions (OH^-) further convert the absorbed CO_2 into carbonate species (*12*). The pH of each solution was recorded at one-second intervals to monitor the CO_2 absorption process. Fig. S10 shows the pH–time profiles of six 1.5 M amines and KOH (reference), revealing distinct kinetic behaviours. Based on the time required for the pH to stabilize, the order of capture rate from fastest to slowest is PZ (37.07 min) < KOH (47.17 min) < EDA (47.78 min) < MP (54.85 min) < META (57.08 min) < PD (69.88 min) < PLA (89.32 min). The overall pH shift (ΔpH) from the start to full CO_2 saturation follows the order MP (4.27) < META (4.79) < PLA (5.20) < PZ (5.48) < EDA (5.78) < PD (5.82) < KOH (6.78), consistent with ^{13}C NMR results for CO_2 loading (Fig. S10a–b). Analysis of time-dependent OH^- and H^+ concentrations (Fig. S10c–d) indicates that in the early stage, OH^- concentration drops sharply for KOH due to its full dissociation, whereas the change is smaller for amines.

The variation in H^+ concentration becomes more prominent as the reaction proceeds, defining the second stage of absorption. For quantitative comparison (Fig. S10e–f), the time derivatives $\Delta[H^+]/\Delta t$ and $\Delta[OH^-]/\Delta t$ were calculated. The initial $\Delta[OH^-]/\Delta t$ follows the order KOH (-1.1×10^{-2} mol L^{-1} s^{-1}) ≫ PD (-2.1×10^{-3}) > EDA (-1.2×10^{-3}) > PLA (-9.21×10^{-4}) > PZ (-3.05×10^{-4}) > META (-2.4×10^{-4}) > MP (-3.1×10^{-5}), consistent with their pK_b values. This rate mainly reflects the initial CO_2 absorption stage and becomes negligible after about 10 minutes. Given that the amine–CO_2 reactions occur predominantly between pH 11 and 7, $\Delta[H^+]/\Delta t$ serves as a more representative metric for the overall capture rate (*14-17*). The trend in $\Delta[H^+]/\Delta t$ shows that META and MP exhibit a rapid initial increase due to fast carbamate formation, followed by a marked decline corresponding to the slow hydrolysis of carbamate to carbonate. This explains why both solutions require nearly one hour to reach saturation. In contrast, PD and PLA exhibit much smaller initial peaks in $\Delta[H^+]/\Delta t$ and substantially longer saturation times, indicating weak carbamate stability and preferential hydrolysis into bicarbonate. The absence of a secondary peak in $\Delta[H^+]/\Delta t$, unlike KOH where CO_3^{2-} reacts further with CO_2 to form HCO_3^-, further supports this conclusion. Diamines such as EDA and PZ, each possessing two nucleophilic nitrogen sites, maintain higher $\Delta[H^+]/\Delta t$ throughout the reaction and reach saturation rapidly even after the rate begins to decline. This behavior, corroborated by ^{13}C NMR (which shows 70–100 % of CO_2 as ionized amine–CO_2 species), confirms a robust carbamate-based mechanism. The peak $\Delta[H^+]/\Delta t$ values rank as follows: PZ (6.8×10^{-11}) > EDA (6.2×10^{-11}) ≫ MP (3.8×10^{-11}) > META (2.7×10^{-11}) ≫ PD (1.6×10^{-11}) > PLA (7.6×10^{-12}).

Note S2: Catalyst design and optimization for electrolysis of amine-captured CO_2.

Our concepts for catalyst design and modulation are delineated below. Differs from traditional gaseous CO_2 electrolysis, the potential reactant species for this process may include, but are not limited to, gaseous CO_2 molecules, amine carbamate ions, bicarbonate/carbonate ions, and amine ammonium ions (*5, 9, 61*). We therefore anticipated that classical Ni-based catalysts, which have been extensively proven to be effective for gas-phase CO_2 electrolysis, might also be applicable in the electrolysis of amine-captured CO_2 systems. This could additionally provide a foundation for the further refinement and optimization of these catalysts (*62*). Consequently, we employed Ni as the active centre and developed a series of Ni nanoparticle catalysts, each with varying N content, by incorporating different ligands. Specifically, we chose 2–MI, BDC, and BTC as ligand precursors, with nitrogen atom percentages decreasing from 34.13% to 7.73% and 0.00%, respectively. We then synthesized the corresponding catalysts, Ni–2MI, Ni-BDC, and Ni-BTC, using a consistent protocol that involved metal impregnation followed by thermal annealing. Additionally, we utilized acid-washing to eliminate any surface Ni nanoparticles, ensuring the efficacy of this modulation in the electrolysis system involving amine-captured CO_2.

Structure characterization of Ni NPs: As shown in Fig. S12a, the XRD patterns of the as-prepared catalysts are depicted, where three apparent diffraction peaks at 44.1, 51.4 and 75.9° can be ascribed to (111), (200), and (220) planes of metallic Ni (PDF #04-0850). The broad peak at 25.9° can be well indexed to (002) facet of graphitic carbon (PDF #75-1621). This confirms the successful incorporation of metal nanoparticles into the carbon-based support after the annealing process. Notably, after acid treatment, the diffraction peak intensities of Ni–2MI–A are significantly reduced compared to Ni–2MI, due to the removal of surface-exposed, unencapsulated nickel clusters on the carbon shell.

The surface oxidation state and chemical position of Ni NPs were investigated by XPS (Fig. S12b−h). The Ni 2*p* XPS spectra (Fig. S12c−e) can be deconvoluted into Ni^{2+} $2p_{1/2}$ (~872.5eV), Ni^{2+} $2p_{3/2}$ (~854.6eV), Ni^0 $2p_{1/2}$ (~870.3eV), Ni^0 $2p_{3/2}$ (~853.1eV), and satellite (~859.2eV and~877.8eV) Ni species, respectively. The two peaks at higher binding energies (BEs) of 872.5 and 854.6 eV can be attributed to possible Ni–X (X = C/N/O) coordination, indicating the presence of Ni in a higher oxidation state (likely Ni^{2+}). In constant, the two peaks at the BEs of 870.3 and 853.1eV correspond to metallic Ni nanoparticles, representing the Ni^0 oxidation state. It is important to note that the Ni^0 peak in the Ni 2p spectra for Ni–2MI–A shows a significant reduction compared to Ni–2MI, indicating that a large proportion of surface Ni particles were removed during the acid wash process. This observation aligns with the TEM and XRD results, which show a clear removal of surface Ni particles in Ni–2MI–A, accompanied by a significant reduction in the intensity of the Ni diffraction peaks. The BEs of Ni $2p_{3/2}$ in Ni–2MI and Ni–2MI–A were recorded at 854.6 eV and 855.0 eV, respectively, approximately 1.0 eV lower than the binding energy of Ni^{2+} in nickel phthalocyanine (NiPc,

856.0 eV). This discrepancy suggests that the Ni species are likely to remain in a low oxidation state ($Ni^{\delta+}$) (*62*). Deconvolution of N 1s XPS spectra results in the five configurations, oxidized N (~403.2eV), quaternary N (~401.4eV), pyrrolic N (~400.2eV), Ni−N (399.5eV), and pyridinic (398.4eV) N species. After acid washing, Ni–2MI–A shows a notable decrease in Ni-N bonds compared to Ni–2MI (Fig. S12f−h). In contrast to Ni–BDC, where nitrogen is present as pyrrolic-N, nitrogen in both Ni–2MI and Ni–2MI–A exists as pyridinic-N. No significant N1s peak was observed in Ni–BTC, as it lacks nitrogen content. The morphology of the Ni–2MI and Ni–2Mi–A were characterized by TEM in (Fig. S13−15). In general, it's clear to observe the metallic Ni spherical particles (dark dots) are covered uniformly inside the carbon skeleton (lattice fringes) which have and average diameter of about 13.2 nm for Ni–2MI and 11.5 nm for Ni–2MI . In the core region, the crystal plane of Ni (111) has a typical lattice spacing of 0.21nm, and the shell region of the graphitic carbon (002) has a spacing of 0.35nm for both Ni–2MI and Ni–2MI–A . Compared with Ni–2MI, Ni–2MI–A only lost the exposed Ni (111) cluster structure on the carbon shell, which confirmed that only the unwrapped Ni cluster was removed during the acid etching, but the main structure is not vandalized. Elemental-mapping images of the Ni–2MI (Fig. S13) catalysts indicate that all four elements of C, N, O and Ni are homogeneous distributed.

The oxidation states of Ni content of the catalysts are elaborated by the Ni K-edge X-ray absorption near edge structure (XANES) (Fig. S16). Compared with bulk Ni and NiO, Ni absorption edge position of both Ni–2MI and Ni–2MI–A are nearly identical to the Ni foil, which indicates that the major Ni contents in the nanoparticles are reduced to zero valency state. The result is conforming to the XPS, where most of Ni^{2+} in the precursor was reduced to metallic state. Fig. S16c is the Fourier transformed Ni K-edge EXAFS for Ni NP catalysts, in which characteristics peaks of both catalysts are matching to the Ni foil. The prominent peak at 2.1 Å reflects the inter-atomic distance between two Ni atoms, which is consistent with the Ni lattice spacing in the Ni (111) cluster measured in TEM. The Wavelet Transform analysis of EXAFS (Fig. S16e−h) further corroborates the atomic-level structural information.

Catalyst evaluation for the electrochemical reduction of CO_2: Five of the synthesized catalysts are evaluated in both 0.5 M $KHCO_3$ for gaseous CO_2 reduction and 1.5 M PZ-CO_2 for amine-captured CO_2 reduction, with no additional CO_2 introduced during the reduction process (for amine solution). For all five obtained catalysts, the sole reduction products identified in both the $KHCO_3$, and PZ-CO_2 capture solutions are CO and H_2. Additionally, no liquid products are detected in the NMR spectra of electrolytes following electrolysis. To maintain consistent reaction conditions with the amine-CO_2 reduction, this part of the gaseous CO_2 reduction is conducted on TGP-H-060 carbon paper in 0.5 M $KHCO_3$. The inlet flow rate of CO_2 is 10 sccm. The FE_{CO} of Ni–2MI and Ni–BDC are found to reach ~60% to 70% at a wider potential range from –0.77 V to –1.07 V vs RHE, while the value for Ni-BTC only approach ~15% at an applied potential of −0.77 V vs. RHE (Fig. S18−19). Particularly, N-doped carbon (NC) is found to be

inert for CO_2 reduction as the FE_{H2} is almost 100% under every applied potential (Fig. S20). The catalyst Ni–2MI–A, after acid washing, exhibits a trend in FE_{CO} that is very similar to that of the catalyst before the acid treatment. To further conform the catalyst performance mentioned in the literature, the CO_2 reduction capabilities of the catalysts Ni–2MI and Ni–BDC were tested on TGP-H-090 30% PTFE carbon paper. It was found that both catalysts achieved a FE_{CO} over 85% at applied potentials ranging from –0.71 V to –1.01 V. These results align well with tremendous research results in conventional gas CO_2 electrolysis community (*18, 37, 62*).

In the electrolysis of 1.5 M PZ-captured CO_2 system, the FE_{CO} of Ni–2MI is superior to both Ni–2MI–A and Ni–BDC under a range of applied current density from –18 mA cm^{-2} to –54 mA cm^{-2}. The highest FE_{CO} of Ni–2MI in 1.5 M PZ-CO_2 can achieve to 65.3%. After acid wash the FE_{CO} shows an obvious decline. As depicted in Fig. S18c, the semicircle diameter in the EIS corresponds to Rct values of 19.5, 33.4 and 26.0 ohm under onset potential (E_{OP}) for Ni–2MI, Ni–2MI–A, and Ni–BDC catalysts, respectively. It indicates that the configuration of Ni–2MI facilitates the electron transfer to amine-based species among the three catalysts. The removal of surface Ni cluster from the carbon shell of the nanoparticles can be clearly confirmed by TEM images in Fig. S13–14. This observation suggests that in the amine-CO_2 reduction system, due to difference in the reactants, more active sites may be required to enhance the reactivity of this kinetically sluggish reaction. Additionally, the exposed Ni atoms might exhibit specific adsorption properties towards protonated PZ carbamates (H^+PZCOO^-). Interestingly, neither Ni–BTC nor N–C exhibits signal indicative of CO or other CO_2 reduction products, suggesting that without N species or metal nanoparticles, the catalyst primarily enhances HER activity in amine capture solution. Furthermore, decent FE_{CO} values of Ni–2MI in the electrolysis of other amine-captured CO_2 solutions (EDA, MEA, and MP) also manifest the purposely designed Ni-based catalyst is efficient in the electrolysis of amine-captured CO_2 system (Fig. S22). Based on these results, Ni nanoparticles immobilized on the carbon support and coordinated with surrounding C/N/O atoms are identified as the key active sites responsible for the enhanced catalytic performance, confirming the justification of our catalyst design and modulation concept. Second, be different from conventional gas CO_2 electrolysis, the surrounding unencapsulated Ni nanoparticles might provide additional catalytic active sites because the removal of the surface Ni clusters leads to an apparent drop of FE_{CO} under every applied potential, suggesting that the Ni clusters exhibit greater catalytic activity towards amine-based species (carbamate) compared to their presumed activity in the $KHCO_3$ system for the HER. Third, employing three ligand precursors results in Ni NPs/carbon heterostructures with or without varying N content, where the electrochemical results manifest that a certain amount of N content is necessary for the enhancing of the FE_{CO} in the electrolysis of amine-captured CO_2 system. Overall, our catalyst design and modulation processes demonstrate that Ni nanoparticles immobilized on the carbon support, featuring Ni–Ni metallic

bonding and Ni–X (X = C/N/O), particularly N, configuration as well as an optimal content are demonstrated to be very efficient for the electrolysis of amine-captured CO_2 system. Also, the geometrically exposed Ni clusters at the catalyst's surface are particularly active for reducing amine-based species and resulting in higher FE_{CO}, which is a distinct result that has rarely been reported in conventional gas CO_2 electrolysis. In the next, we employed Ni–2MI as the key catalyst and thoroughly studied the reaction mechanism in the electrolysis of PZ-captured CO_2 solution and other amine-captured CO_2 systems.

Note S3: Methodology for the electrochemical evaluation of amine-CO_2 reduction and FE determination in a H-Cell.

In contrast to conventional evaluation methods for gaseous CO_2 electrochemical reduction, the amine– CO_2 electrocatalytic reduction was performed in a closed system without external CO_2 feed during operation. All experiments were conducted in a 50 mL H-cell (Gaoss Union Pty Ltd., China). As illustrated in the accompanying schematic, CO_2 reduction occurred in the compartment equipped with valves to maintain airtight conditions throughout the reaction. All electrochemical measurements were carried out under ambient conditions (293.15 K, 101.3 kPa).

Amine-CO_2 solution preparation for electrochemical tests: The amine–CO_2 solutions were prepared using a standardized procedure to ensure consistent CO_2 loading and reproducible electrochemical performance. Taking 1 L of 1.5 M PZ–100% CO_2 solution as an example, 1.5 mol of PZ (129.2 g) was dissolved in 500 mL of Milli-Q water in a 1 L reagent bottle. CO_2 gas was then continuously bubbled into the mixture at a flow rate of 100 mL min^{-1}. During CO_2 absorption, the solid PZ gradually dissolved, and the solution temperature rose owing to the exothermic nature of the reaction. When the pH stabilized, indicating completion of CO_2 absorption, 500 mL of the resulting CO_2-rich solution (after cooling to room temperature) was transferred into a 1 L volumetric flask and diluted to the mark with Milli-Q water. The prepared solution was stored at 4°C to minimize degradation. Amine solutions containing supporting electrolytes such as 2 M KCl were prepared following the same procedure, with KCl added prior to the final dilution to ensure precise concentration control. This method was adopted to (i) dissipate the heat generated during CO_2 uptake and (ii) compensate for the observable increase in solution volume as CO_2 is absorbed. Partially loaded amine–x% CO_2 solutions were obtained by volumetric mixing of the fully loaded (100% CO_2) and unloaded amine solutions (*5*).

Electrochemical Testing in the H-cell: Electrochemical tests were conducted in a sealed 50 mL H-cell equipped with three electrodes, as schematically shown in Fig. S21. The anode and cathode chambers were separated by a Nafion 117 membrane. The headspace volume above the catholyte, determined by the water-displacement method, was 80 mL after accounting for the solution volume. TGP-H-060 carbon paper served as the substrate for catalyst loading. Prior to use, the carbon paper was ultrasonically cleaned in acetone three times for 1 h each and dried overnight at 60 °C. Before electrolysis, CO_2 was bubbled through the catholyte at 10 mL min^{-1} for 30 min to restore full loading. In the electrocatalytic reduction of amine-CO_2 under 1 a.t.m. CO_2, the anolyte consists of 50ml 1 M KOH, while the catholyte contains the 50ml 1.5 M required amine solution. The flow rate of CO_2 gas in letting the H-cell gas chamber was precisely controlled between 10 and 20 sccm using a DGFC. Before the reaction starts, a continuous flow of CO_2 at 20 sccm is introduced into the reactor for at least 30 minutes to ensure the electrolyte is fully saturated with CO_2.

Note S4: Methodology for the electrochemical evaluation of PTFE-modified electrodes.

In experiments investigating the effect of PTFE on electrochemical CO_2 reduction, CO_2 was continuously introduced into the reactor at a constant flow rate. TGP-H-090 with 5 wt% and 30 wt% PTFE were used without further treatment. The catalyst ink was prepared by dispersing 10 mg of catalyst in 165 μL Milli-Q water, 310 μL isopropanol, and 25 μL of 5 wt% Nafion®. PTFE nanoparticle loadings of 20%, 40%, and 60% were obtained by adding 4.17, 11.12, and 25 mg of Teflon DISP 30, respectively, followed by sonication for 1 h individually and 2 h after mixing. The homogeneous slurry was drop-cast onto 1 × 1 cm^{-2} carbon paper (effective area) and dried at room temperature, giving a catalyst loading of 1 mg cm^{-2}. Contact angles of 87.9°, 145.7°, and 167.1° were measured for TGP-H-090 5 wt% PTFE, 30 wt% PTFE, and 30 wt% PTFE with additional 60 wt% PTFE, respectively (Fig. S25). These correspond to hydrophilic, hydrophobic, and superhydrophobic surface. The outlet was connected to a gas bag for product collection. After the reaction, the total gas volume was obtained by summing the introduced CO_2 and the gas accumulated in the reactor headspace.

Note S5: Diffusion coefficient estimation and diffusion layer thickness calculation.

PTFE surface modification is widely used to enhance electrochemical CO_2 reduction, as numerous studies have shown that introducing PTFE can lower the overpotential and improve carbon-related FE. This effect is generally attributed to the hydrophobic microenvironment created by PTFE within the gas diffusion layer, which repels the liquid electrolyte and facilitates the retention and transport of gaseous CO_2 near catalyst particles, thereby increasing the local CO_2 concentration at the electrode surface (*19*). All PZ-CO_2 and gas-phase CO_2 reduction experiments in this section were conducted under an identical CO_2 flow rate of 10 sccm (Note S4). Under these conditions, the persistent decline in FE_{CO} with increasing PTFE coverage, despite continuous gas-phase CO_2 supply, indicates that gaseous CO_2 is not the dominant carbon source in the PZ-CO_2 reduction. Instead, these observations provide further evidence that a significant fraction of the reduced CO_2 originates from PZ-chemisorbed species in the liquid phase. In the following section, we describe how the EIS response can be used to interpret this phenomenon and how it relates to mass transport at the electrode interface.

The diffusion coefficient of CO_2 in 0.5 M $KHCO_3$ was estimated using Fuller's empirical formula (Eq. S12) (*63*).

$$D_0 = \frac{435.7\,T^{3/2}}{P(V_A^{1/3}+V_B^{1/3})^2}\sqrt{\frac{1}{\mu_A}+\frac{1}{\mu_B}} \quad \text{(S12)}$$

where T (K) is temperature, P (Pa) is pressure, V_A and V_B are molar volumes of CO_2 and H_2O (m^3 mol^{-1}), and μA and μB are viscosities (cP). The resulting diffusion coefficient of CO_2 in water is 1.6×10^{-9} m^2 s^{-1}.

The diffusion coefficient (Eq. S13) of protonated PZ–carbamate in a 1.5 M PZ–100% CO_2 solution was estimated using the empirical correlation by Versteeg and van Swaaij (*64, 65*):

$$\frac{D_0}{D} = \left(\frac{\mu_{amine\,solution}}{\mu_{water}}\right)^{0.6} \quad \text{(S13)}$$

where $\mu_{amine\,solution}$ and μ_{water} are the dynamic viscosities (Pa s). The diffusion coefficient D can also be correlated with temperature and concentration (Eq. S14):

$$\ln D = 13.672 - \frac{2160.9}{T} - 19.263 \cdot 10^{-5} C \quad \text{(S14)}$$

where C is the amine concentration (mol m^{-3}). The diffusion coefficient of PZ–carbamate in water is estimated to be 4.73×10^{-10} m^2 s^{-1}. Thus, the D_0 relevant to the CO_2 reduction reaction is assumed to be constant in both the PZ–CO_2 and $KHCO_3$ electrolytes.

An impedance-based approach was employed to characterize diffusion-related transport under electrocatalytic conditions, following established treatments in gaseous CO_2 electroreduction systems (*66*). The electrode–electrolyte interfaces can exhibit finite-diffusion characteristics in the low-frequency impedance response. When a Randles-type equivalent circuit is used, the charge-transfer resistance (R_{ct}) and the double-layer capacitance (C_{dl}) are connected in parallel

to describe the interfacial charge-transfer process. In CO_2 reduction, including the amine–CO_2 electroreduction system investigated here, R_{ct} reflects the kinetic barrier associated with interfacial electron-transfer steps involving CO_2-derived species (e.g., dissolved CO_2 or protonated PZ carbamate intermediates), while C_{dl} represents the interfacial charge-storage capability of the electrode. The characteristic time constant (τ) for the interfacial process is given by the parallel RC combination (Eq. S15):

$$\tau = R_d C_d \tag{S15}$$

At the electrode–electrolyte interface, τ represents a characteristic mass-transport timescale. Following a standard diffusion scaling argument derived from Fick's second law, τ can be related to the diffusion-layer thickness (δ) and the diffusion coefficient (D) of the reacting species via (Eq. S16) (*67*):

$$\tau \sim \frac{\delta^2}{D} \tag{S16}$$

Under steady-state, diffusion-limited conditions, the maximum achievable reaction flux is governed by this effective transport length scale, yielding the classical expression for the limiting current density (Eq. S17) (*68*):

$$j_{\mathrm{lim}} = nF \cdot \frac{D_0 C_0}{\delta} \tag{S17}$$

where j_{lim} is the limiting current density (A m^{-2}), n is the number of transferred electrons, and C_0 is the concentration of the electroactive species (mol m^{-3})

Taken together, Eq. 15–16 establish a quantitative link between the EIS response and δ. The τ is first extracted from the diffusion-related impedance features measured by EIS (Fig. S26). τ can be mapped onto δ through the diffusion scaling relation, thereby building a bridge between the experimentally accessible impedance response and interfacial mass transport. A shorter τ corresponds to a smaller effective transport thickness, indicating faster diffusive replenishment of reactants at the electrode surface. Under steady-state, diffusion-limited conditions, this reduced transport thickness leads to a higher j_{lim} according to Eq. 17. Consequently, improved mass transport, reflected by a smaller δ inferred from EIS, is directly associated with enhanced electrochemical performance.

Thus, the carbamate participation proportion was estimated using a comparative FE_{CO} approach under controlled CO_2 availability, by combining validation from PTFE microenvironment modulation experiments with results obtained at various CO_2 loadings (Fig. S26). The underlying assumption of this analysis is that, under full-loading conditions, both dissolved CO_2 and carbamate species are accessible for electrochemical reduction. Accordingly, the measured FE_{CO} under full CO_2 loading (FE_{CO}^{full}) reflects the combined contributions from gaseous CO_2 and carbamate-derived carbon sources ($FE_{CO}^{full} = FE_{CO}^{CO_2} + FE_{CO}^{carbamate}$). In contrast, N_2 purging and partial-loading preparation significantly reduce the equilibrium CO_2 partial

pressure, thereby suppressing the concentration of dissolved CO_2 in solution while largely retaining carbamate species, where the contribution of FE_{CO} under low-loading conditions (FE_{CO}^{low}) can be reasonably assumed to originate predominantly from carbamate species ($FE_{CO}^{low} \approx FE_{CO}^{carbamate}$). Under these conditions, the observed decrease in FE_{CO} can therefore be statistically attributed to the reduced contribution from molecular CO_2, allowing the relative participation of carbamate species to be inferred by comparison with the full-loading case. Importantly, this calculation methodology is applicable only to amine–CO_2 systems where carbamate species have been independently validated as electrochemically active carbon sources. The Eq. 18 shows the proportion of carbamate participation at each CO_2 loading under a specific applied current. In the present work, this prerequisite is supported by the PTFE microenvironment experiments, where enhanced interfacial hydrophobicity leads to suppressed CO selectivity in multi-/diamine systems such as PZ, consistent with carbamate-dominated reaction pathways. Accordingly, the methodology may be extended to other amine-captured CO_2 systems that exhibit similar carbamate reactivity, but it is not expected to be universally applicable to bicarbonate-dominated or monoamine systems without such validation.

$$P_{carbamate} = \frac{FE_{CO}^{low}}{FE_{CO}^{full}} \tag{S18}$$

Note S6: Hydrophobicity evaluation across a broad range of amine solutions.

Based on the findings related to the $KHCO_3$ and PZ-CO_2 systems, we aim to further validate this conclusion by extending the study to a broader range of amines. We conducted CO_2 electrochemical reduction using the same reaction conditions as in the last section, in 1.5 M solutions of META, MP, and EDA. The experiments were performed under two microenvironments: on TGP-H-090 with 5% PTFE and on TGP-H-090 with 30% PTFE, with an additional 60% PTFE incorporated into the catalyst. In Fig. S27a–d, the changes in impedance at the E_{OP} under two hydrophobic environments are presented. For the MP solution, the introduction of hydrophobic regulation significantly reduces the charge transfer resistance, while for EDA and PZ, it leads to an increase in charge transfer resistance. As shown in Fig. S27e–h**,** META, MP, EDA, and PZ exhibit different FE_{CO} results before and after electrode hydrophobic microenvironment modification. META and MP show a significant increase in FE_{CO}, with MP displaying the most notable improvement, transitioning from almost no carbon species involvement in CO_2 reduction to achieving 50% FE_{CO}. Among the diamines, EDA shows similar results to PZ, further indicating that amine carbamate species formed by diamines participate in CO_2 reduction.

Note S7: ATR–FTIR investigation of functional group adsorption on electrode surfaces during reduction reactions

Simulated IR spectra of PZ-CO_2 related species: Due to the complexity of species present in the PZ-CO_2 solution, we conducted structure optimization on the potential species in the solution, including PZ dicarbamate, PZ carbamate, protonated PZ carbamate, and protonated PZ (Fig. S11, Table S15). This approach provides a reference for interpreting the data obtained from ATR–FTIR measurements. Based on previous work and cross-referencing spectra, we have obtained a table of functional group appearance ranges (*23, 24*).

In situ ATR–FTIR investigation of PZ-CO_2 reduction intermediates: To further identify surface-adsorbed functional groups under applied bias, in situ ATR–FTIR measurements were conducted on Ni–2MI and the metal-free catalyst NC over a potential range from 0 to −2.5 V vs. RHE. The peak observed at 855.6 cm^{-1} (Fig. S29) is characteristic of the N−H twisting vibration, a common feature for both primary and secondary amines within the 600−900 cm^{-1} region (*69*). Previous studies have noted that the most significant change in the ATR–FTIR spectrum of PZ upon CO_2 absorption is the splitting of the peak at 1274.2 cm^{-1} into two distinct peaks at 1288.6 and 1261.7 cm^{-1}. This spectral change is indicative of the formation of PZ mono- and di-carbamates during the absorption process (*23*).

Note S8: DFT calculations to investigate the energetics and mechanism of the process.

The construction of the catalyst model: To construct a physically meaningful yet computationally tractable model for the graphene-encapsulated Ni catalyst (Ni–2MI, Fig. S12–16), a localized region of the curved nanoparticle surface was approximated as a planar interface, represented by an N-doped graphene slab supporting surface Ni_n clusters. Large-scale machine-learning-assisted atomic simulations were employed to screen Ni_n clusters with sizes ranging from n = 3 to 20, where hundreds to thousands of initial geometries were generated for each size and structurally relaxed using a neural network (NN) potential to identify global minimum (GM) isomers and systematically explore the potential energy surface. The stability of various gas-phase clusters was first assessed using the second-order difference energy ($\Delta^2 E$), which revealed pronounced positive values for Ni_6, Ni_7, Ni_{13}, Ni_{15}, Ni_{17}, and Ni_{19}, indicative of enhanced intrinsic stability (Fig. S31a). Guided by the experimentally observed size distribution from STEM (Fig. S13), Ni_6, Ni_7, Ni_{13}, and Ni_{15} were identified as physically relevant candidates (Fig. S32) rather than assuming a single cluster size. These clusters were subsequently evaluated on an N_4-doped graphene substrate to account for support effects. While larger clusters exhibit stronger electronic interaction energies (E_{int}) due to increased coordination with the substrate, they also undergo substantial structural deformation upon adsorption, resulting in significant deformation energy penalties ($E_{def} \geq 1.00$ eV). In contrast, the Ni_6 cluster achieves the most favourable overall adsorption energetics (Fig. S31b), corroborated by its high adsorption energy E_{ads} of −8.12 eV with minimal structural distortion (E_{def} = 0.30 eV). Consistent with STEM observations that resolve both Ni atoms embedded within the carbon shell and surface-attached Ni clusters, the optimised Ni_6 configuration places one Ni atom at the outermost surface of the N-doped graphene sheet while the remaining five Ni atoms reside above the graphene plane, maximising the exposure of accessible Ni sites and forming eight stabilising Ni–N bonds (Fig. S33a). This stable configuration is quantitatively reflected in a high binding energy of –3.37 eV, which confirms the strong anchoring of Ni_6 to the substrate.Collectively, the agreement between the STEM-constrained size distribution, gas-phase cluster stability, and support-inclusive energetic analysis supports Ni_6 as a stable and representative cluster motif for the Ni–2MI system, while acknowledging the coexistence of multiple cluster sizes.

PZ-CO_2 reduction reaction pathways and mechanism: The reaction pathways (Table S16−17, Fig. 4d, and Fig. S34) are predominantly dictated by the thermodynamic stability of key intermediates. To reveal the catalytic advantages of surface clusters in PZ-CO_2 reduction, we systematically compared the reaction energetics on Ni−2MI and Ni−2MI−A configurations (Fig. S35). In the electrochemical reduction of protonated PZ carbamate, the pathway is initiated by the thermodynamically favourable adsorption of protonated PZ carbamate on either the Ni_6 cluster or the isolated Ni–N_4 site.

As shown in Fig. S35c, upon the involvement of a proton, HPZ–COO* undergoes hydrogenation, forming HPZ–COOH*, with the free energy increasing to –2.61 eV. As the reaction progresses, this stabilized intermediate, HPZ–COOH*, facilitates further electron transfer, leading to the reduction of HPZ–COOH* to HPZ–CO*. This step is marked by an additional drop in the free energy (–2.88 eV), reflecting the enhanced stability of the intermediate on the catalyst surface. Subsequently, the C–O bond in HPZ–COOH* preferentially cleaves, leading to the formation of PZCO*. From this intermediate, two possible pathways are identified: (i) initial C–N bond cleavage followed by intramolecular hydrogen migration within the HPZ–N* fragment to regenerate the PZ ring; or (ii) proton transfer occurring first, followed by C–N bond cleavage. By comparing the free and reaction energies of the two routes, we find that the pathway involving initial C–N bond cleavage is energetically more favourable. Thus, the Gibbs free energy change for steps 1–6 can be expressed in Table S17.

Note S9: The determination of irmation energy and structure optimization.

The capture reactions are based on Eq. S1–11 from Note S1. Results are shown in Table S18. As shown in Fig. 4f, $E_{formation}$ are determined by subtracting the total energy of the reactants from that of the products. Compared across six structurally similar amines, META and MP, both containing −OH groups, exhibit significantly higher (lower absolute) carbamate formation energies. This indicates that the electron-withdrawing effect of the −OH group hinders carbamate formation, resulting in weaker formation capacities for MP and META. PLA and PD, containing weak electron-donating $-CH_3$ groups, exhibit lower (higher absolute) carbamate formation energies, consistent with their lower p*K*b values (Table S1). This suggests that the formed carbamates are unstable and undergo hydrolysis to produce bicarbonate/carbonate ions , aligning with their NMR results (Fig. S2−7). Thus, PZ and EDA, with moderate carbamate formation energies, are identified as suitable candidates for integrated amine-CO_2 reduction processes as capture agents.

Note S10: Zero-gap electrolyzer setup and efficiency calculation.

In our evaluation of the electrocatalytic reduction of PZ–CO_2 in a cyclic system using the zero-gap electrolyzer, we designed three experiments for different purposes. The first experiment focused on CO_2 reduction and amine regeneration, the second on testing the stability of the catalyst, and the third on energy efficiency and carbon efficiency during the reduction process on a larger scale.

Catalyst durability test: To ensure the stability of both the catalyst and the capture system, a 210-hour cycling test was performed to evaluate long-term performance and catalyst durability (Fig. S39). A 400 mL solution of 3 M PZ saturated with CO_2 was stored in a 500 mL reagent bottle. An AEM was used to separate the anode and cathode compartments. The cathode and anode were fabricated from TGP-H-060, coated with Ni−2MI and IrO_2 catalysts, respectively, each at a loading of 1 mg cm^{-2}. During electrolysis, a continuous make-up flow of CO_2 (5 sccm) was introduced into the PZ reservoir to maintain full CO_2 loading. Excess gas was vented to ensure that the internal pressure remained equal to atmospheric pressure. A freshly prepared 2 L solution of 1 M KOH was used as the anolyte at the start of the experiment and was replaced every 24 hours to ensure electrolyte consistency. Throughout the test, CO selectivity remained stable at ~50%, with a steady current density of 120 mA cm^{-2}.

Continuous PZ–CO_2 reduction from CO_2-saturated state using various ion exchange membranes: To identify the most suitable membrane configuration for achieving integrated CO_2 capture and conversion in a zero-gap electrolyzer, electrolysis experiments were conducted using 3 M PZ solutions saturated with CO_2 in conjunction with PEM, AEM, and BPM. To account for the distinct ionic environments introduced by each membrane type, the corresponding anolyte compositions were adjusted accordingly, as summarized in Table S19.

To investigate the CO_2 capture and conversion performance of 3 M PZ under low-concentration CO_2 conditions, a series of gas mixtures with varying CO_2 contents were introduced into the solution using the bubble column method described in Note S1. All experiments were conducted under a fixed total gas flow rate of 100 mL min^{-1}. The CO_2 volume fractions were adjusted by mixing CO_2 and N_2 at defined flow rates: 40 vol% CO_2 (40 mL min^{-1} CO_2, 60 mL min^{-1} N_2), 20 vol% CO_2 (20 mL min^{-1} CO_2, 80 mL min^{-1} N_2), 10 vol% CO_2 (10 mL min^{-1} CO_2, 90 mL min^{-1} N_2), 5 vol% CO_2 (5 mL min^{-1} CO_2, 95 mL min^{-1} N_2), and 1 vol% CO_2 (1 mL min^{-1} CO_2, 99 mL min^{-1} N_2). For each condition, CO_2 was bubbled into the PZ solution until saturation was reached. During CO_2 bubbling, the solution pH was continuously recorded to monitor the CO_2 absorption dynamics and evaluate PZ's capacity for capturing CO_2 from low-concentration CO_2 streams. After saturation was reached under each condition, the final CO_2 loading was quantified using ^{13}C NMR analysis, following the procedure described in Note S1.

Electrolysis of 3 M PZ solution saturated with CO_2 was performed under constant current conditions to evaluate membrane-dependent behaviour. Fig. S42–S44 illustrate the long-term electrolysis performance of PZ–CO_2 systems using three different ion exchange membranes: PEM, AEM, and BPM. During each test, 100 mL of 3 M PZ saturated with CO_2 was stored in a 100 mL reagent bottle (with an approximate 35 mL gas headspace). After saturation, the headspace and tubing were purged with N_2 at a flow rate of 10 mL min^{-1} for 10 mins to ensure that any evolved CO_2 originated exclusively from the solution, eliminating gas-phase CO_2 interference. Gas products were first passed through a mass flow meter and then analysed by the online GC, beginning at the 10-minute mark and subsequently measured every ~27.55 minutes. FE_{CO}, FE_{CH4}, and FE_{H2} were calculated according to Eq. S19. Simultaneously, cumulative gas products were collected in a 2 L gas bag and their total volume measured using the water displacement method. The composition of the accumulated gases was analysed via offline GC. For PEM and BPM configurations, where negligible carbon crossover was observed, the electrolyte was sampled after 24 and 48 hours, respectively, to quantify residual CO_2 loading. In contrast, for the AEM configuration, which exhibited evident carbon crossover, electrolyte samples were collected at 2, 4, 6, 8, and 24 hours and analysed via ^{13}C NMR.

$$\mathrm{FE} = \frac{zF}{I} \cdot \frac{yu}{V_{\mathrm{m}}} \quad \text{(S19)}$$

where I (A) is the applied current during electrolysis, u ($m^3\ s^{-1}$) is the flow rate of the generated gas, and Vm ($m^3\ mol^{-1}$) is the molar volume (0.0241 $m^3\ mol^{-1}$ at 25 °C and 1 atm).

The evaluation of full-cell energy efficiency (FCEE) of the zero gap electrolyser tests is conducted using the following Eq. S20:

$$\mathrm{FCEE}\ (\%) = \frac{\mathrm{FE_{CO}} \cdot E^0}{E_{\mathrm{cell}}} \quad \text{(S20)}$$

where E^0 is the equilibrium cell potential for CO production ($E^0 = E^0_{cathode} - E^0_{anode} = -0.1\mathrm{V} - 1.23\mathrm{V} = -1.33\mathrm{V}$). E_{cell} is the applied overall cell voltage.

The CO_2 loading during electrolysis was calculated by subtracting the total amount of CO_2 converted to CO and CH_4 (Eq. S21), as well as the amount of CO_2 desorbed, from the initial CO_2 loading.

$$\mathrm{CO_2\ loading\ (mol\ CO_2/mol\ PZ)} = \frac{n_{\mathrm{CO_2\ total}} - n_{\mathrm{CO}} - n_{\mathrm{CH_4}} - n_{\mathrm{CO_2}}}{n_{\mathrm{PZ}}} \quad \text{(S21)}$$

where n_{CO} and n_{CH4} were calculated from FE_{CO} and FE_{CH4} using Faraday's law, based on the applied current, reaction time, and the number of electrons involved in each product formation; n_{CO2} was was calculated based on the measured CO_2 volume fraction (vol%) and the total gas flow rate, assuming ideal gas behaviour at 25 °C and 1 atm.

Carbon utilization was calculated as the ratio of the total moles of electrochemically generated CO (n_{CO}) and CH_4 (n_{CH4}) to the amount of CO_2 desorbed from the solution during electrolysis (Eq. S22).

$$\text{Carbon utilization (\%)} = \frac{n_{\mathrm{CO}}+n_{\mathrm{CH_4}}}{(\alpha_{\mathrm{ini}}-\alpha_{\mathrm{post}})\cdot V_{\mathrm{pz}}\cdot M_{\mathrm{pz}}} \tag{S22}$$

Carbon crossover in the AEM system was quantified using Eq. S23. The carbon crossover coefficient a ($0 < a < 1$) represents the ratio between the experimentally observed carbon loss and the theoretical maximum carbon transfer derived from the applied current. Specifically, a was calculated as the ratio between the difference—the amount of desorbed CO_2 (measured by ^{13}C NMR at a given time point) minus the total amount of carbon released in the form of gaseous products (CO_2, CO, and CH_4)—and the theoretical carbon transfer expected based on Faraday's law. Under the assumption that all charge is transported by HCO_3^-, the theoretical maximum carbon crossover rate is 0.0104 mmol A^{-1} s^{-1}. The carbon crossover coefficient a was further evaluated across different stages of CO_2 loading depletion. Specifically, in the CO_2 loading intervals of 1.02–0.97, 0.97–0.92, 0.92–0.86, 0.86–0.78, and 0.78–0.685 mol CO_2 mol^{-1} PZ, the corresponding values of a were determined to be 0.67, 0.62, 0.59, 0.54, and 0.49, respectively.

$$n_{\mathrm{CO_2\ crossover}} = a \cdot \frac{I \cdot t}{96485} \tag{S23}$$

ICCE in a scaled-up electrolyser: Fig. S45–S47 illustrate the setup for reactive capture in a 9 cm^2 zero-gap electrolyser. A 2 L reagent bottle served dually as the catholyte reservoir and the CO_2 absorption bubble column, pre-filled with 1.8 L of 3 M PZ. Prior to electrolysis, the solution was saturated with simulated flue gas composed of 20 vol% CO_2 balanced with N_2. During electrolysis, the same simulated flue gas (20 vol% CO_2, N_2-balanced) was continuously introduced into the reservoir at 12- or 24-mL min^{-1}, corresponding to 3-or 6-mL min^{-1} of CO_2, respectively. Electrolysis was carried out under a constant current of 450 mA (50 mA cm^{-2}). The anolyte consisted of 1 L of circulating 2 M KOH. For the BPM configuration, electrolysis was conducted continuously without replacement of electrolyte solutions. In contrast, for the AEM configuration, the KOH anolyte was refreshed every 48 hours, and the PZ solution was re-saturated with the simulated flue gas prior to resuming electrolysis.

The single-pass CO_2-to-CO efficiency was evaluated using the Eq. S24:

$$\mathrm{CO_2\text{-to-CO\ efficiency}} = \frac{\mathrm{FE_{CO}} \cdot I \cdot V_m}{V_{\mathrm{CO_2,\ in}} \cdot z \cdot F} \tag{S24}$$

The carbon efficiency is determined by the Eq. S25,

$$\text{Carbon Efficiency} = \frac{\Delta\alpha \cdot n_{PZ}}{n_{\mathrm{crossover}}} \tag{S25}$$

where Δα (mol CO_2 mol^{-1} PZ) is mol amount of CO_2 before and after reaction.

Note S11: Energy efficiency analysis.

To compare the energy consumption between two pathways—using pure CO_2 as a feed gas and tandem amine-CO_2 reduction—we analyzed the energy required to produce an equivalent amount of CO. Currently, CO_2 electrolysis relies on a purified CO_2 feed, necessitating the use of flue gas (12% CO_2) as the raw feedstock and generating pure CO as the final product. In this scenario, the conventional CO_2 pathway involves absorption, stripping, reboiling, and compression processes to regenerate the amine sorbent and obtain high-purity CO_2. In contrast, the tandem CO_2 reduction pathway considers CO_2 capture via amine adsorption as an integrated feedstock, bypassing the need for extensive purification. This comparison provides critical insights into the relative energy efficiency of these two approaches and their implications for scalable CO_2 electrolysis. Accordingly, our energy analysis is based on a daily production of **100,000 kg of CO** to ensure a robust evaluation of industrial feasibility.

Energy estimation for CO_2 feedstock: Amine scrubbing for post-combustion CO_2 capture is a well-established technology. The first-generation solvent, META, has reduced its energy demand from 0.37–0.51 MWh/ton CO_2 to 0.19–0.28 MWh ton^{-1} CO_2 (*70*). Approximately half of this energy is consumed as low-temperature heat (steam) for the stripper reboiler, while the other half is used for compression to 150 bar for transport and sequestration (*70*). As the CO_2-amine reaction is spontaneous, energy consumption in the absorber column is minimal. Based on reported data, the reboiler duty for 30 wt% META ranges from 3.6–4.0 GJ ton^{-1} CO_2, while 40 wt% META reduces it to 3.1–3.3 GJ ton^{-1} CO_2. A 40 wt% (8 mol l^{-1}) PZ solution achieves 2.9 GJ ton^{-1} CO_2 (*71*), with the lowest reported value of 2.0 GJ ton^{-1} CO_2 using a META + MDEA (Methyldiethanolamine, variable mix ratio) system (*72*). Accordingly, we estimate the total process energy consumption to fall within the range of 4.0–8.0 GJ ton^{-1} CO_2. Therefore, we assume an energy consumption of **6.0 GJ ton^{-1} CO_2** for high concentration CO_2 feed in the conventional CO_2 reduction process. In contrast, for tandem CO_2 reduction, only the capture step is required, accounting for approximately 5% of the overall energy demand. As a result, the energy consumption for obtaining the captured CO_2 solution is estimated to be **0.3 GJ ton^{-1} CO_2**.

Pathway 1: conventional gas-phase CO_2 reduction: Current benchmark studies on CO_2 reduction in zero gap electrolyzers using pure CO_2 as the feed gas report FE_{CO} ranging from 90% to 99%, current densities between 100 and 300 mA cm^{-2}, and operating voltages in the range of 2.5 to 4.0 V. Additionally, the single-pass CO_2 utilization efficiency is typically around 5–25% for neutral CO_2 reduction (*28*). In this study, we use a FE_{CO} of 95%, a current density of 200 mA cm^{-2}, a CO_2 utilization efficiency of 25%, and an applied potential of 3.2 V as the representative example for our calculations.

To produce 100,000 kg of CO per day with 95% FE_{CO}, the required total current is defined as:

Total current = 100,000 kg day^{-1} × 1/86400 day s^{-1} × 1000/28.01 mol kg^{-1} × $2e^{-}$ × 96485 C mol^{-1} × 1/0.95 = 8,393,427 A

When the single-pass conversion of CO to CO_2 is 25%, the required CO_2 inlet flow rate is:

Inlet CO_2 flow rate = 100,000 kg day^{-1} × 1/0.95 × 1/0.25 × 44 g mol^{-1} × 1/28 mol g^{-1} = 661,654.1 kg day^{-1}.

Inlet CO_2 molar flow rate = 661,654.1 kg day^{-1} × 1/44 mol g^{-1} × 1000g kg^{-1} = 15,037,594 mol day^{-1}.

Thus, the energy required to produce this amount of high-purity CO_2 from flue gas is:

E (CO_2 acquisition) = 661,654.1 kg day^{-1} × 0.001 kg ton^{-1} × 6.0 GJ ton^{-1} CO_2 = **3969.9 GJ day^{-1}**.

Considering that electrolyte circulation accounts for 5% of the total electrolysis power consumption, based on estimations from water electrolysis studies, the energy consumption on the electrolyzer is (*73*):

E (electrolyser) = 8,393,427 A × 3.2 V × 86400 s day^{-1} × 1.05 = **2,436.6 GJ day^{-1}.**

To obtain high-purity CO, we employ Pressure Swing Adsorption (PSA) to separate CO from a CO-H_2 gas mixture. In previous studies, the energy consumption for gas separation has often been estimated by analogy with biogas upgrading, which involves the separation of methane and CO_2 in roughly equal proportions. Given that the gas stream exiting the electrolyzer typically exhibits a CO_2/CO ratio close to 1:1, several works have adopted an energy cost of 0.25 kWh m^3 as a reasonable approximation for the separation process.

E (separation) = 0.25 kWh m^{-3} × (34,176,349 mol day^{-1} + 100,000 kg day^{-1} × [(1/0.95)–1] × 1/28 mol g^{-1} × 1000 g kg^{-1}) × 8.314 J mol^{-1} K^{-1} × 298.15 K × 1/101325 Pa^{-1} = **279.9 GJ day^{-1}**.

The total energy consumption is:

E (total) = *E* (CO_2 acquisition) + *E* (electrolyser) + *E* (separation) = **6686.4 GJ day^{-1}**.

E (per ton) = **6686.4 GJ day^{-1}** × 1/100,000 day kg^{-1} × 1000 kg ton^{-1} = **66.86 GJ ton^{-1}**.

Pathway 2: our tandem amine capture and CO_2 reduction strategy: The calculation for the tandem CO_2 zero-gap electrolyser is based on the average values obtained from tested cycles using 9 cm^2 MEA under direct feeding of 20 vol% CO_2. Based on the BPM configuration, with an FE_{CO} of 37%, an overall current density of 50 mA cm^{-2}, and a full-cell voltage of 3.2 V, the CO_2 utilization per cycle can reach up to 100% due to the negligible CO_2 crossover. In our calculations, a conservative value of 90% was used. In the AEM configuration, with an FE_{CO} of approximately 42.1%, an overall current density of 50 mA cm^{-2}, and a full-cell voltage of

2.3 V, the CO_2 utilization is limited to around 36% due to carbon crossover. For consistency, the AEM configuration is used as the representative case in our calculations.

Total current = 100,000 kg day^{-1} × 1/86400 day s^{-1} × 1000/28.01 mol kg^{-1} × 2e^{-} × 96485 C mol^{-1} × 1/0.421 = 18,940,038.6 A.

The required active CO_2 in the PZ solution flow rate is:

Inlet CO_2 flow rate = 100,000 kg day^{-1} × 1/0.37 × 1/0.421 × 44 g mol^{-1} × 1/28 mol g^{-1} = 1,008,813.4 kg day^{-1}.

Inlet CO_2 molar flow rate = 1008813.4 kg day^{-1} × 1/44 mol g^{-1} × 1000g kg^{-1} = 22927576.4 mol day^{-1}

Thus, the energy required to produce this amount of PZ-CO_2 solution from flue gas is:

E (CO_2 acquisition) = 1,008,813.4 kg day^{-1} × 0.001 kg ton^{-1} × 0.3 GJ ton^{-1} CO_2 = **302.6 GJ day^{-1}**.

Considering that electrolyte circulation accounts for 5% of the total electrolysis power consumption, based on estimations from water electrolysis studies, the energy consumption on the electrolyzer is (*73*):

E (electrolyser) = 18,940,038.6 A × 2.3 V × 86400 s day^{-1} × 1.05 = **3763.8 GJ day^{-1}.**

E (separation) = 0.25 kWh m^{-3} × (22927576.4 mol day^{-1} + 100,000 kg day^{-1} × (1/0.421 – 1) × 1/28 mol g^{-1} × 1000 g kg^{-1}) × 8.314 J mol^{-1} K^{-1} × 298.15 K × 1/101325 Pa^{-1} = **612.96 GJ day^{-1}.**

The total energy consumption is:

E (total) = *E* (CO_2 acquisition) + *E* (electrolyser) + *E* (separation) = **4679.4 GJ day^{-1}.**

E (per ton) = **4679.4 GJ day^{-1}** × 1/100,000 day kg^{-1} × 1000 kg ton^{-1} = **46.79 GJ ton^{-1}.**

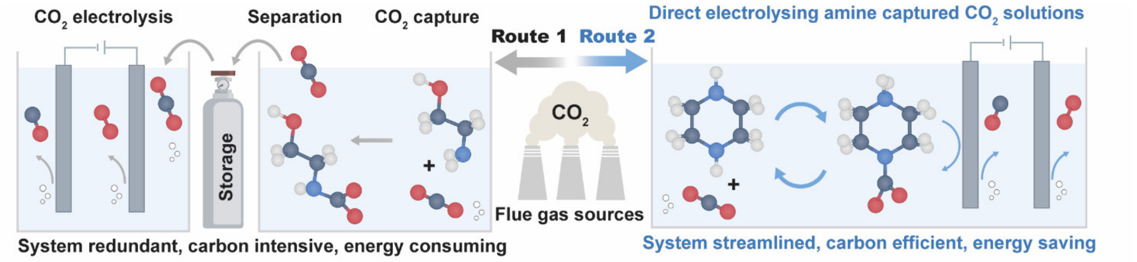

Fig. S1. Comparison of two CO_2 upgrading routes from flue gas.

Route 1 involves traditional CO_2 electrolysis, reliant on CO_2 feedstock from amine scrubbing, followed by separation and storage. Route 2 illustrates a streamlined, tandem approach where captured CO_2 adducts are directly converted into valuable feedstocks via electrolysis, offering enhanced carbon efficiency and energy savings.

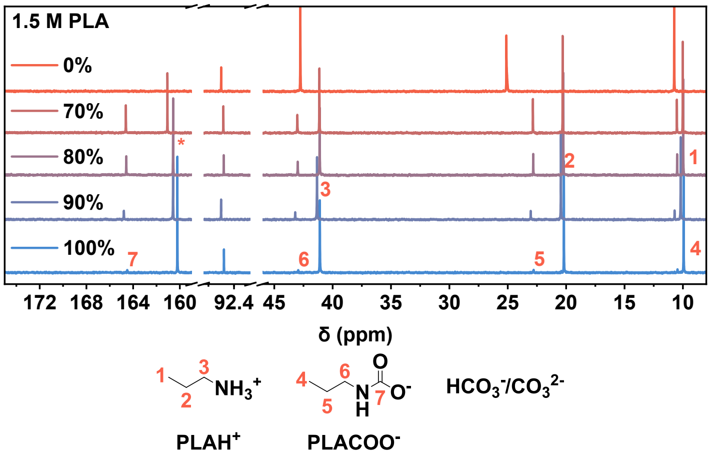

Fig. S2. ^{13}C NMR spectra of 1.5 M PLA aqueous solutions at varying CO_2 loadings (0%, 70%, 80%, 90%, and 100%).

The spectra correspond to different carbon environments in $PLAH^+$ and $PLACOO^-$ with chemical shifts labelled for each identified carbon (C_1-C_5). The peaks marked with * represent carbonate and bicarbonate, as consistently shown in Fig. S2−S7.

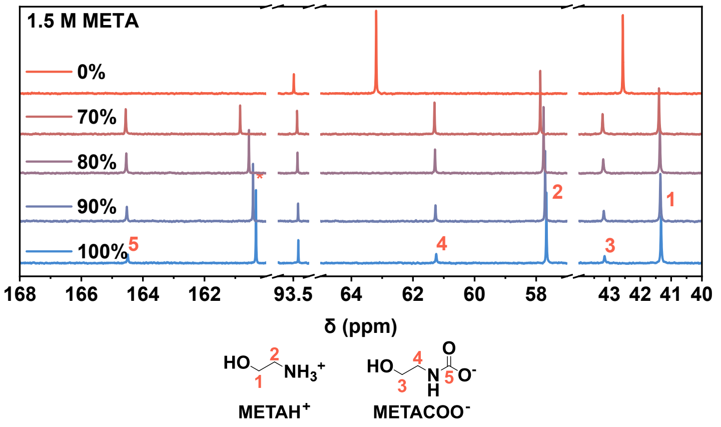

Fig. S3. ^{13}C NMR spectra of 1.5 M META aqueous solutions at varying CO_2 loadings (0%, 70%, 80%, 90%, and 100%).

The spectra correspond to different carbon environments in $METAH^+$ and $METACOO^-$ with chemical shifts labelled for each identified carbon (C_1–C_5).

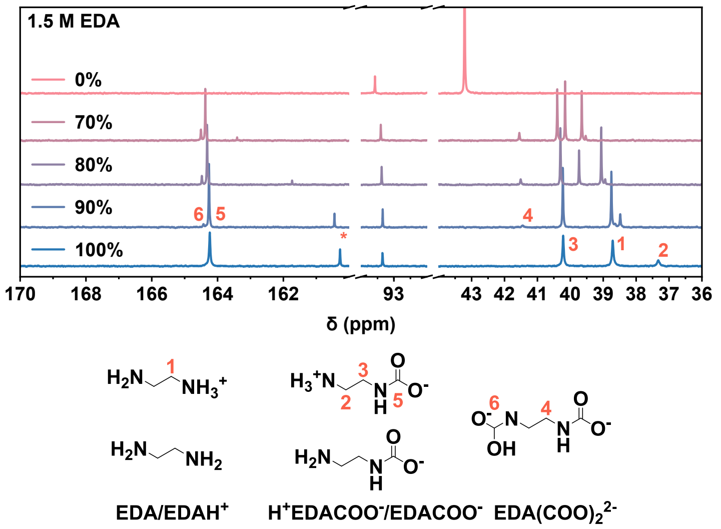

Fig. S4. ^{13}C NMR spectra of 1.5 M EDA aqueous solutions at varying CO_2 loadings (0%, 70%, 80%, 90%, and 100%).

The spectra correspond to different carbon environments in EDA/$EDAH^+$, $H^+EDACOO^-$/$EDACOO^-$, and $EDA(COO)_2^{2-}$ with chemical shifts labelled for each identified carbon (C_1-C_6).

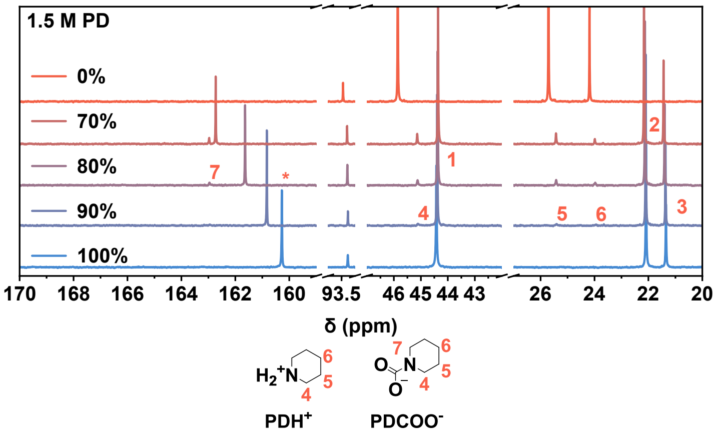

Fig. S5. ^{13}C NMR spectra of 1.5 M PD aqueous solutions at varying CO_2 loadings (0%, 70%, 80%, 90%, and 100%).

The spectra correspond to different carbon environments in PDH^+ and $PDCOO^-$ with chemical shifts labelled for each identified carbon (C_1-C_7).

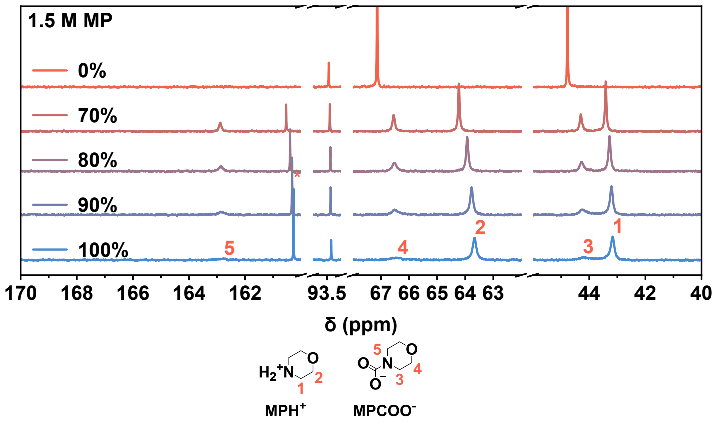

Fig. S6. ^{13}C NMR spectra of 1.5 M MP aqueous solutions at varying CO_2 loadings (0%, 70%, 80%, 90%, and 100%).

The spectra correspond to different carbon environments in MPH^+ and $MPCOO^-$ with chemical shifts labelled for each identified carbon (C_1-C_5).

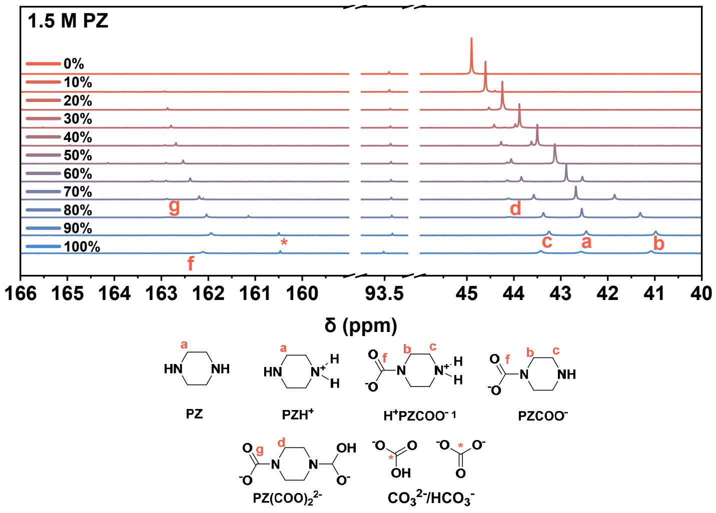

Fig. S7. ^{13}C NMR spectra of 1.5 M PZ aqueous solutions at varying CO_2 loadings (0%, 10%, 20%, 30%, 40%, 50%, 60%, 70%, 80%, 90%, and 100%).

The spectra correspond to different carbon environments in PZ/PZH^+, H^+PZCOO^-/$PZCOO^-$, and $PZ(COO)_2^{2-}$with chemical shifts labelled for each identified carbon (C_a-C_g).

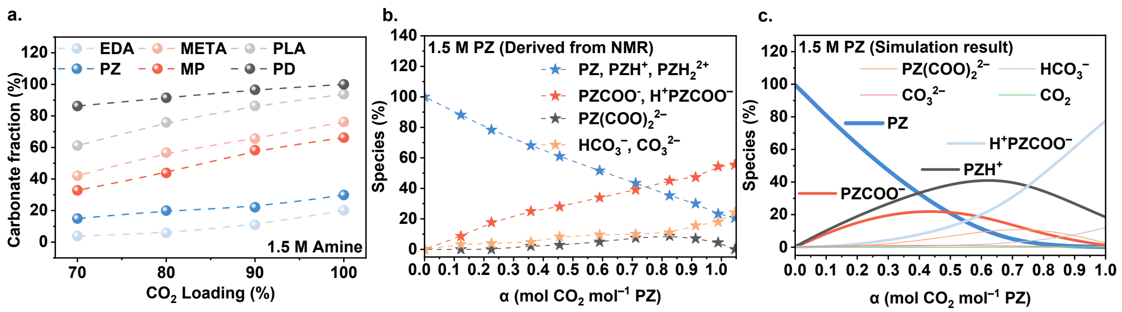

Fig. S8. Distribution of ionic species in amine solutions.

(a) Percentage of CO_2 present as carbonate in six 1.5 M amine solutions. (b) Species distribution derived from ^{13}C NMR analysis. (c) Simulated species distribution (*36*). Both panels illustrate the evolution of chemical species with increasing CO_2 loading. The corresponding numerical data are provided in Table S7.

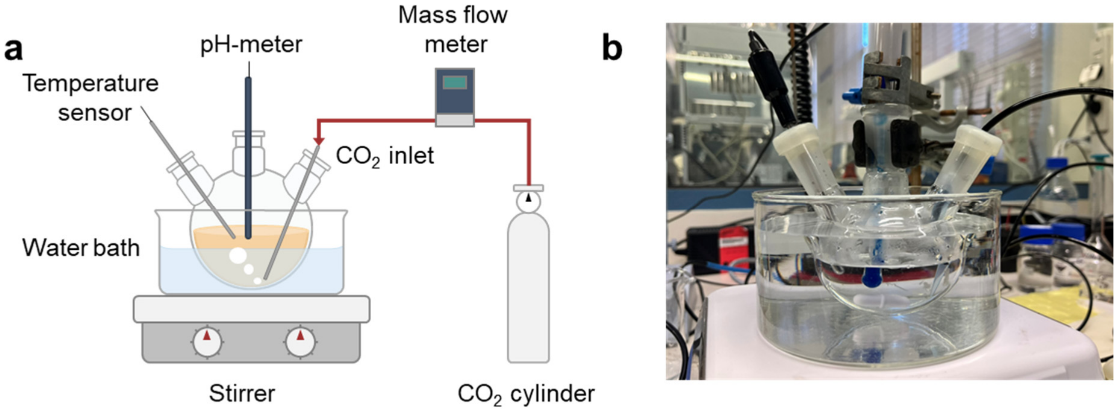

Fig. S9. Bubble column setup.

(a) Schematic of the experimental apparatus for monitoring pH while CO_2 is introduced into amine and KOH solutions. (b) Photograph of the assembled setup.

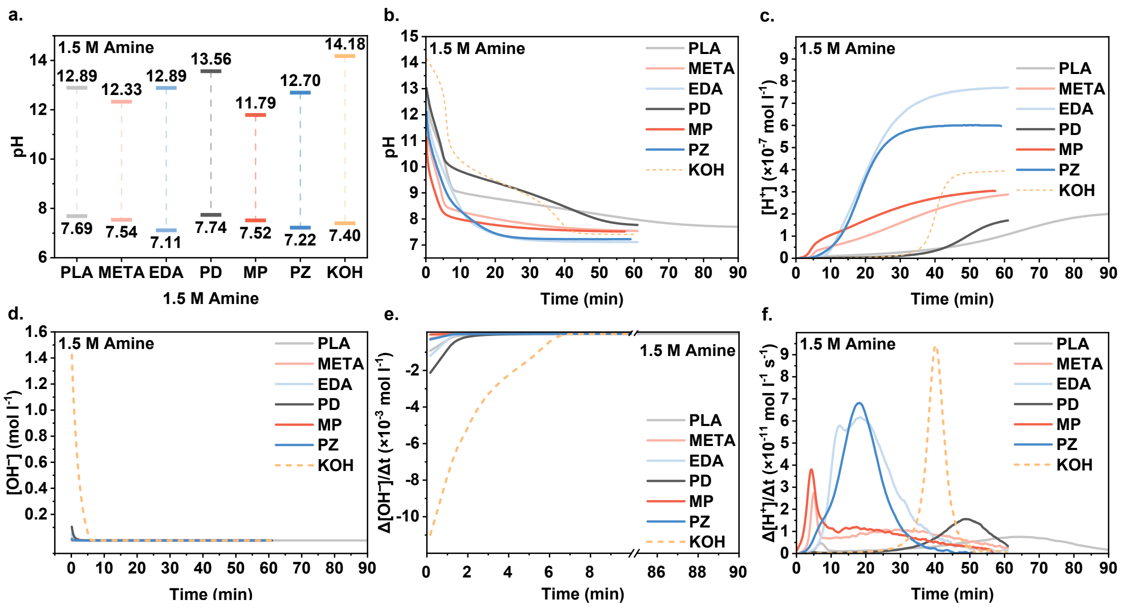

Fig. S10. CO_2 absorption kinetics of amines in a bubble column.

(a) Overall pH change (ΔpH) before and after CO_2 saturation for six 1.5 M amine solutions and KOH (reference). (b) Time-dependent pH profiles recorded during CO_2 absorption, reflecting the distinct stabilization rates of each solution. (c) Variations in $[H^+]$ with time, derived from the pH–loading calibration curve. (d) Variations in $[OH^-]$ with time, showing a sharp initial decrease in KOH followed by slower consumption in amine solutions. (e) $[OH^-]$ variation rate ($[OH^-]/\Delta t$) over time plot. (f) $[H^+]$ variation rate ($[H^+]/\Delta t$) over time plot.

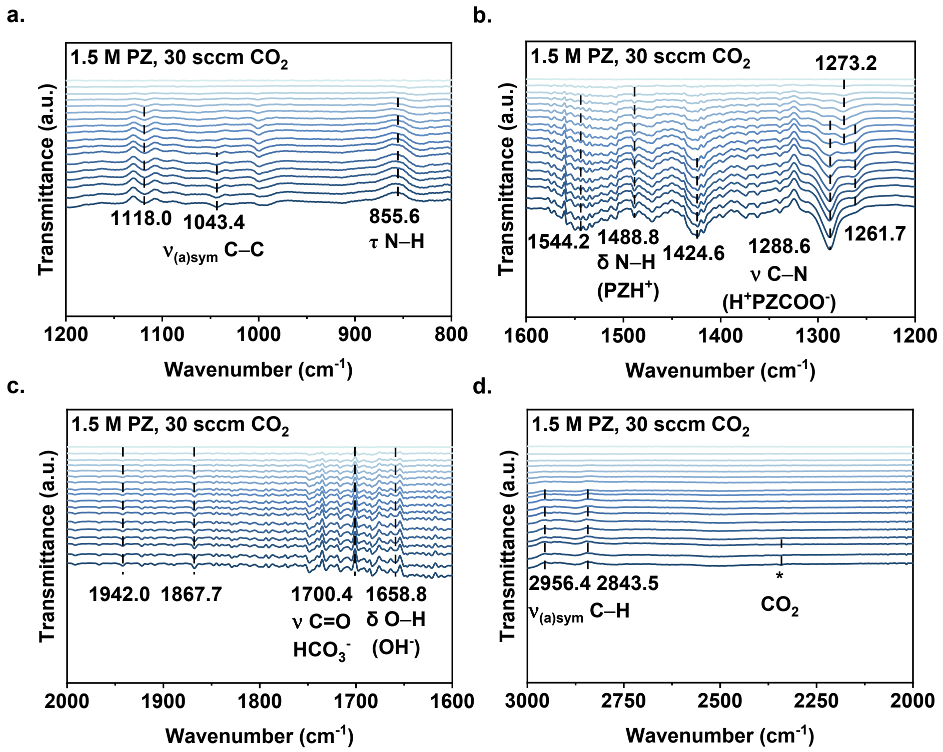

Fig. S11. In situ ATR–FTIR spectra of 1.5 M PZ solution during CO_2 bubbling. (a) 800-1200 cm^{-1}. (b) 1200-1600 cm^{-1}. (c) 1600-2000 cm^{-1}. (d) 2000-3000 cm^{-1}.

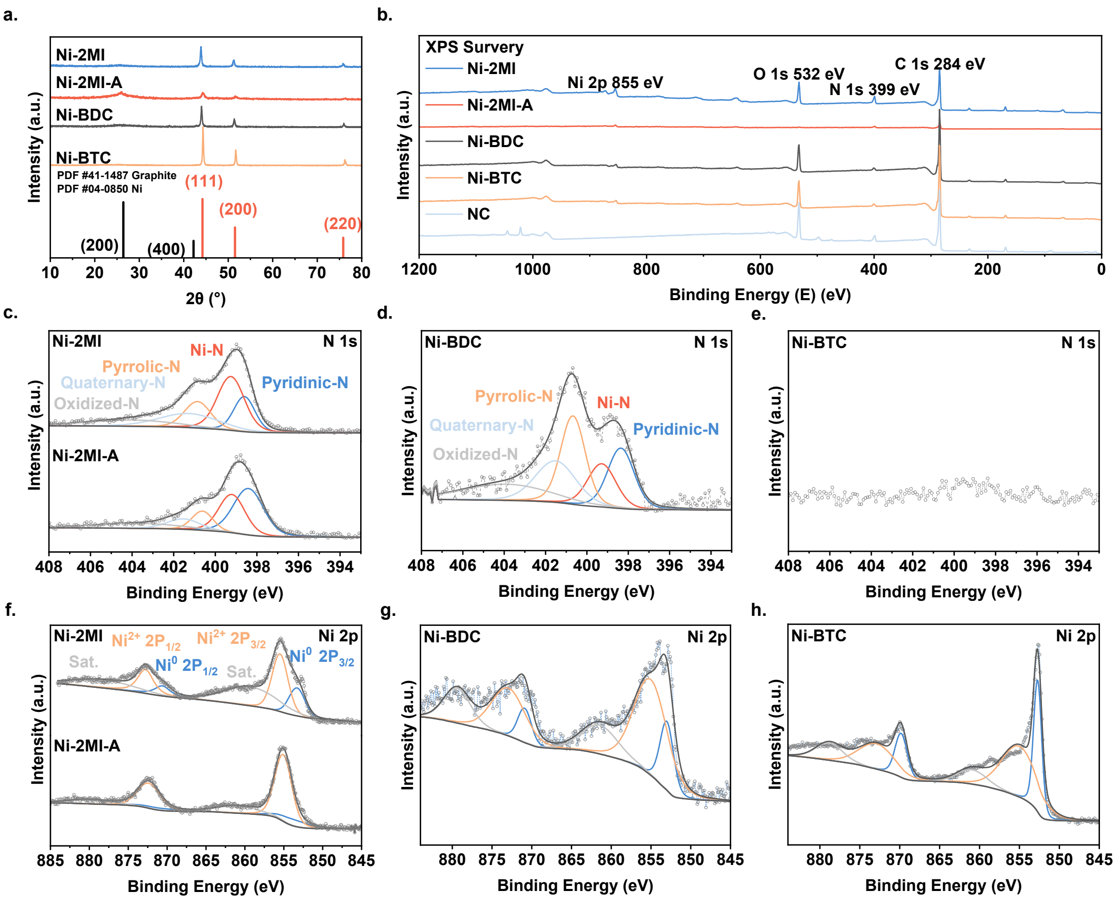

Fig. S12. Structural and surface chemical characterization of catalysts.

(a) XRD patterns of Ni–2MI, Ni–2MI–A, Ni–BDC, and Ni–BTC, showing diffraction peaks corresponding to the (111), (200), and (220) planes of the face-centered-cubic (fcc) Ni phase. (b) XPS survey spectra confirming the presence of Ni, N, C, and O elements in all catalysts. (c–e) High-resolution N 1s spectra, deconvoluted into pyridinic N, Ni–N, pyrrolic N, quaternary N, and oxidized N species. (f–h) Ni 2p spectra showing the coexistence of metallic Ni^0 ($2p_{3/2}$, $2p_{1/2}$) and Ni^{2+} species along with characteristic satellite peaks.

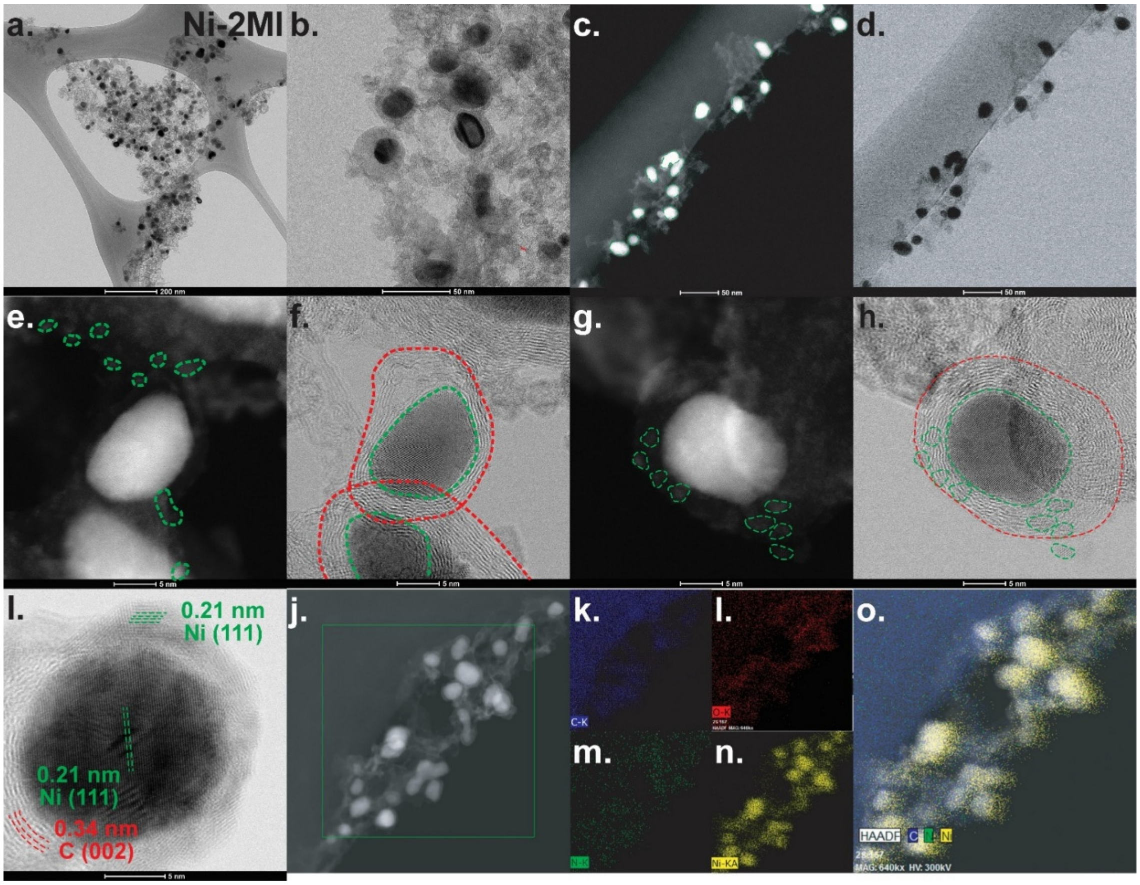

Fig. S13. TEM and High-angle annular dark-field scanning transmission electron microscopy (STEM-HAADF) images of the Ni–2MI catalyst.

(a–d) Low-magnification TEM images showing uniform Ni nanoparticles distributed within the carbon matrix. (e–h) HRTEM and HAADF micrographs reveal a core–shell architecture comprising a Ni nanoparticle (green dashed line) encapsulated by a carbon shell (red dashed line), with Ni clusters (green dash line) decorating the shell surface. (I) High-resolution TEM images reveal lattice spacings of 0.21 nm for Ni (111) and 0.34 nm for C (002), confirming graphitic carbon encapsulation of crystalline Ni. (j–o) DF image and corresponding elemental mappings of the selected region. The overlapped EDS maps (o) confirm the uniform distribution of C (blue), O (red), N (green), and Ni (yellow), indicating the formation of Ni nanoparticles embedded in a nitrogen-doped carbon matrix.

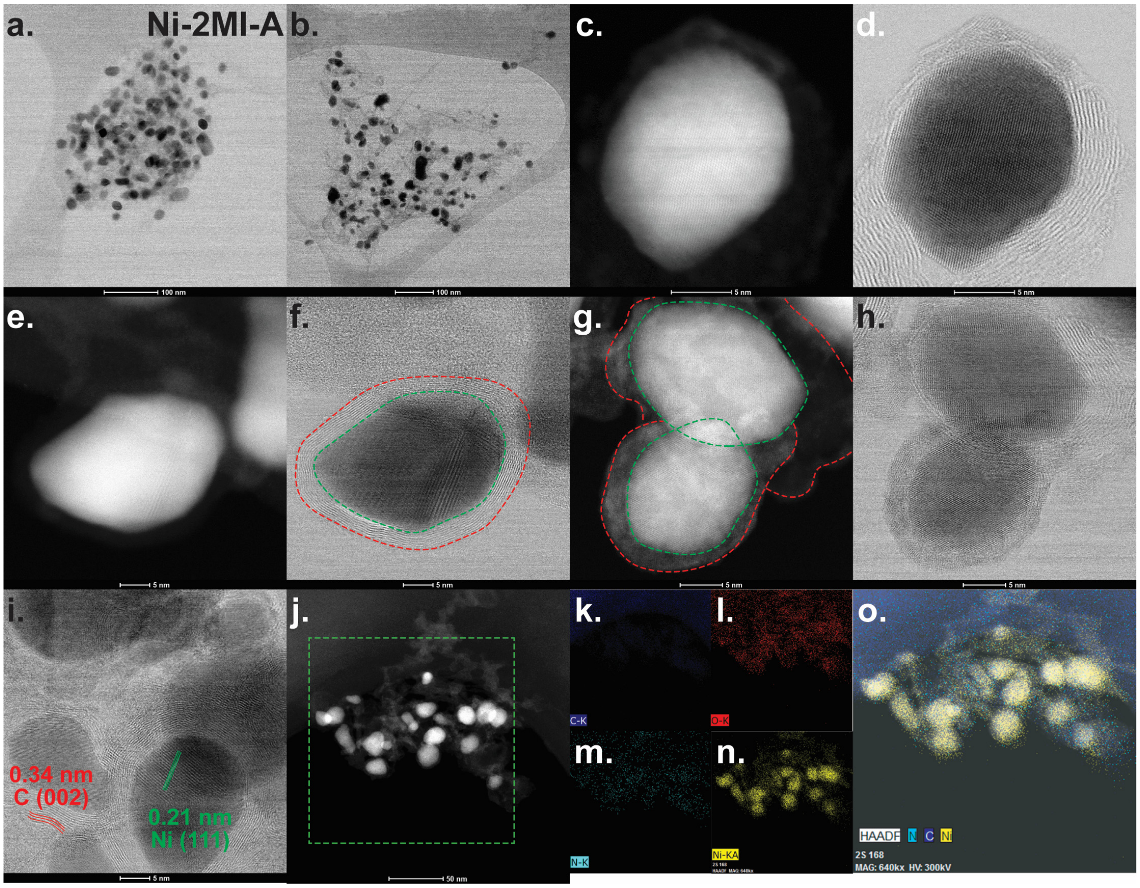

Fig. S14. TEM and STEM-HAADF images of the Ni–2MI–A catalyst.

(a–b) Low-magnification TEM images showing uniform Ni nanoparticles distributed within the carbon matrix. (c–h) HRTEM and HAADF micrographs reveal a core–shell architecture comprising a Ni nanoparticle (green dashed line) encapsulated by a carbon shell (red dashed line). (I) High-resolution TEM images reveal lattice spacings of 0.21 nm for Ni (111) and 0.34 nm for C (002), confirming graphitic carbon encapsulation of crystalline Ni. (j–o) DF image and corresponding elemental mappings of the selected region. The overlapped EDS maps (o) confirm the uniform distribution of C (blue), O (red), N (green), and Ni (yellow), indicating the formation of Ni nanoparticles embedded in a nitrogen-doped carbon matrix.

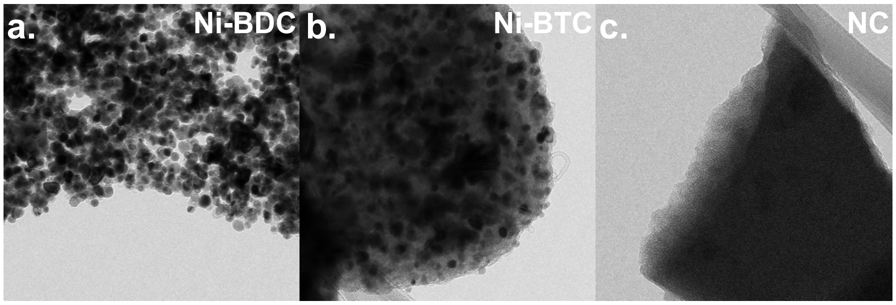

Fig. S15. TEM images of Ni-based precursors and derived carbon materials.

(a) Ni–BDC and (c) Ni–BTC exhibit nanoparticulate morphologies composed of aggregated primary particles, while (c) the corresponding nitrogen-doped carbon (NC) shows a uniform, porous carbon framework after pyrolysis.

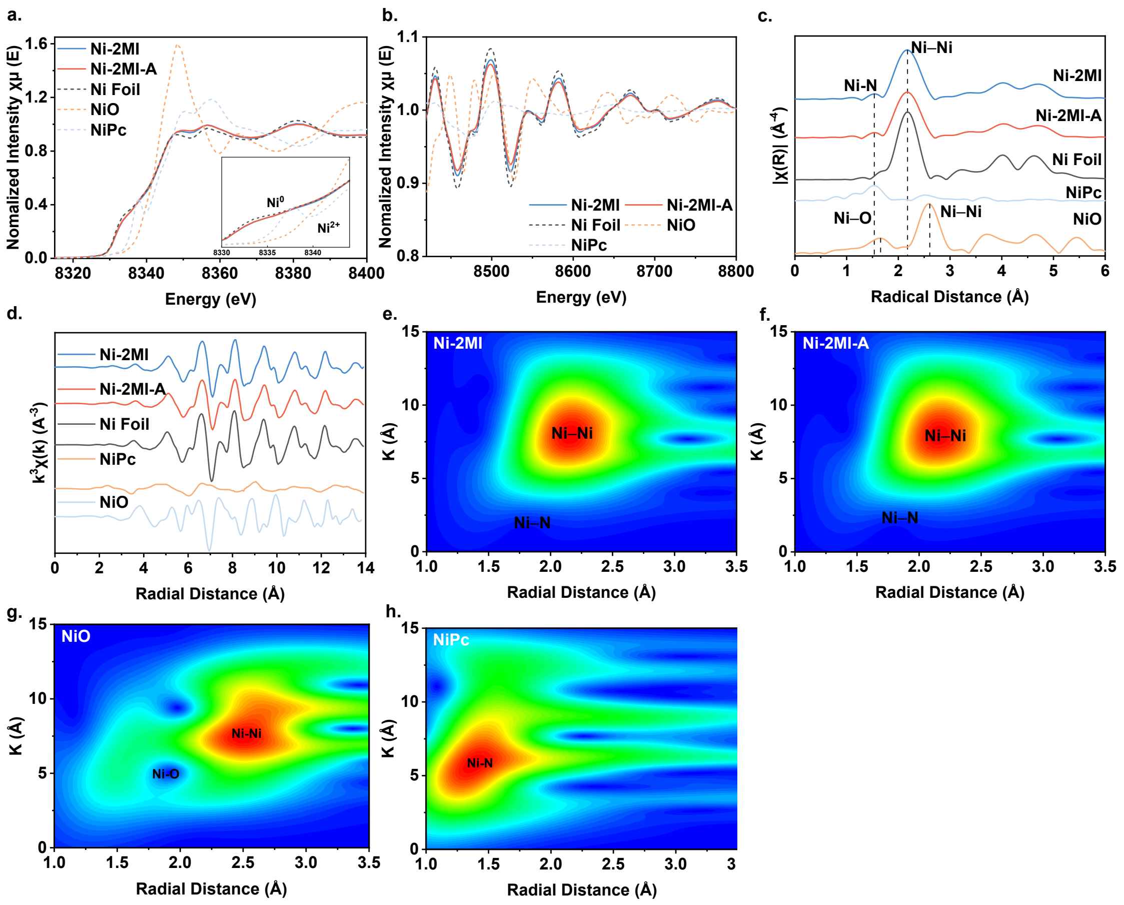

Fig. S16. Synchrotron X-ray absorption spectroscopic analysis of local Ni coordination

(a–b) Ni K-edge XANES (a) and EXAFS (b) spectra of Ni–2MI, Ni–2MI–A, Ni Foil, NiO, and NiPc. (c) Fourier transformation of the EXAFS spectra. (d) Ni K-edge EXAFS oscillations in K-space. (e–h) Wavelet Transform for EXAFS of (e) Ni–2MI, (f) Ni–2MI–A, (g) Ni Foil, and (h) NiPc.

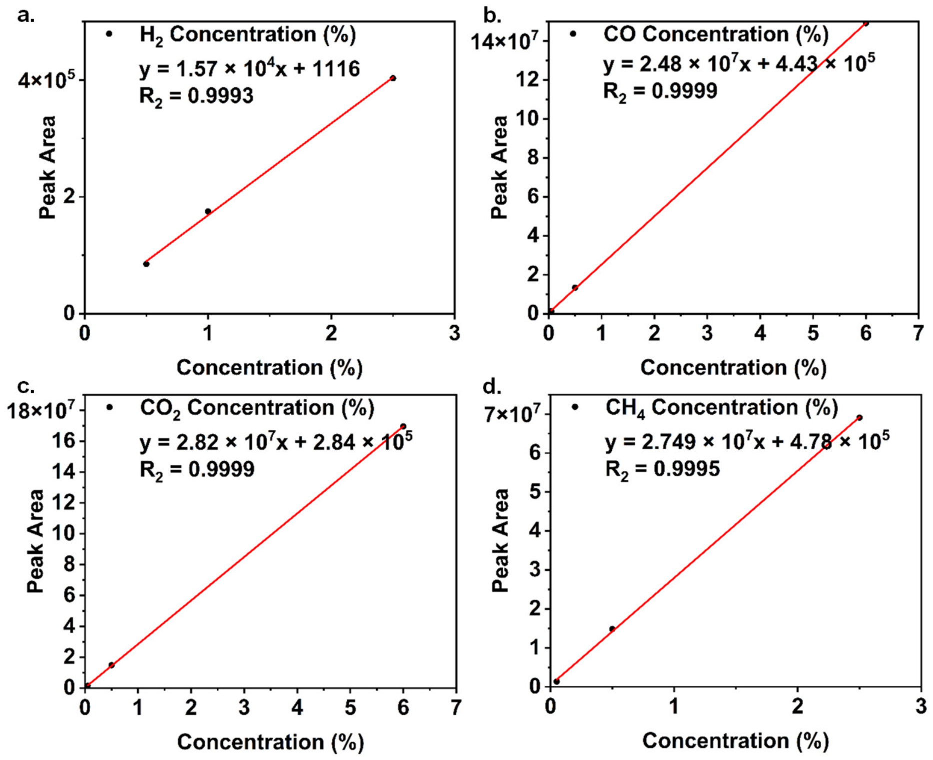

Fig. S17. GC calibration curves for gaseous products.

(a) H_2, (b) CO, (c) CO_2, and (d) CH_4. Each panel shows the linear relationship between gas concentration (%) and GC peak area.

Gas chromatographic calibration was performed using standard mixtures with known compositions: Gas A: 0.50% H_2, 0.05% CO, 0.05% CH_4 0.05% CO_2, 0.25% O_2, 0.50% N_2 (balanced with Ar). Gas B: 1.00% H_2, 0.50% CO, 0.50% CH_4, 0.50% CO_2, 0.50% O_2, 1.00% N_2 (balanced with Ar). Gas C: 2.50% H_2, 6.00% CO, 2.50% CH_4, 6.00% CO_2, 1.00% O_2, 6.00% N_2 (balanced with Ar). Calibration curves were constructed by linear fitting between the integrated GC peak areas and the corresponding gas volume percentages according to Eq. S26:

$$y = a\,x + b \qquad \text{(S26)}$$

where y is the gas volume percentage, x is the integrated peak area, and a and b are the slope and intercept, respectively. All fitting coefficients (R^2) exceeded 0.999.

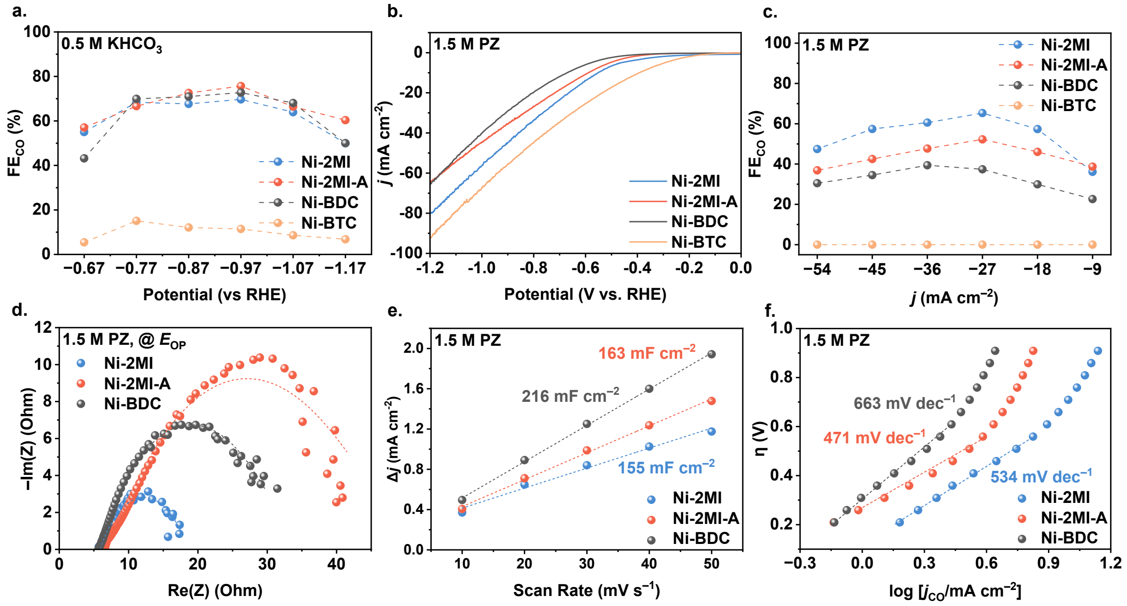

Fig. S18. Additional CO_2 electroreduction in 0.5 M $KHCO_3$.

(a) FE_{CO} of Ni–2MI, Ni–2MI–A, Ni–BDC, and Ni–BTC catalysts at different applied potentials in a 0.5M $KHCO_3$ solution. (b) LSV curves of four catalysts in 1.5 M PZ with full CO_2 loading. (c) FE_{CO} of four catalysts under different current density in 1.5 M PZ. (d) EIS and fit curves for three catalysts collected at E_{OP}. The data points represent the experimental data, while the solid lines indicate the fitted data. (e) The capacitive currents as a function of scan rates for three catalysts. (f) Tafel curve of three catalysts.

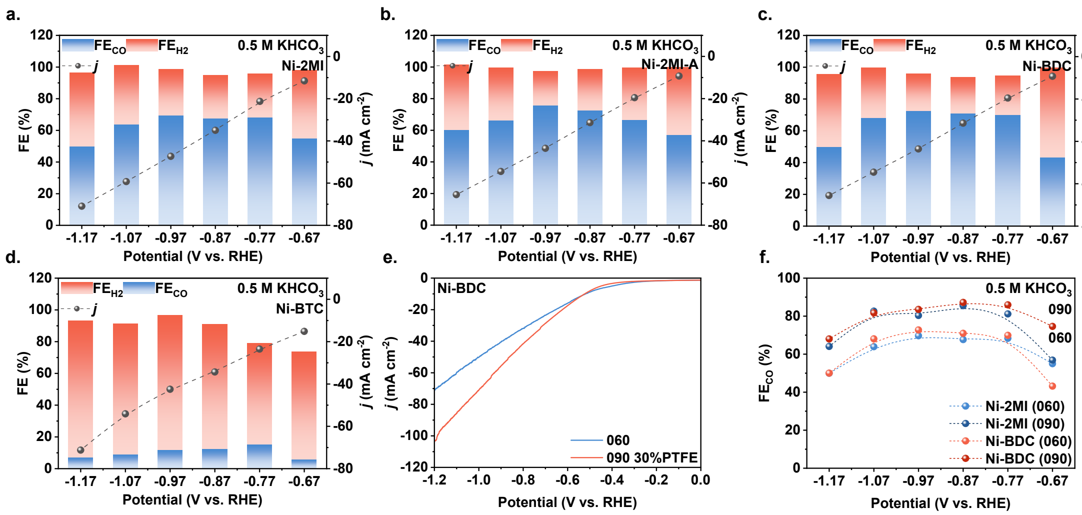

Fig. S19. Additional CO_2 electroreduction in 0.5 M $KHCO_3$.

(a–d) Potential-dependent current density and FE of (a) Ni–2MI, (b) Ni–2MI–A, (c) Ni–BDC, (d) Ni–BTC. (e) LSV curves and (f) Comparison of FE_{CO} for Ni–2MI and Ni–BDC using two different carbon papers.

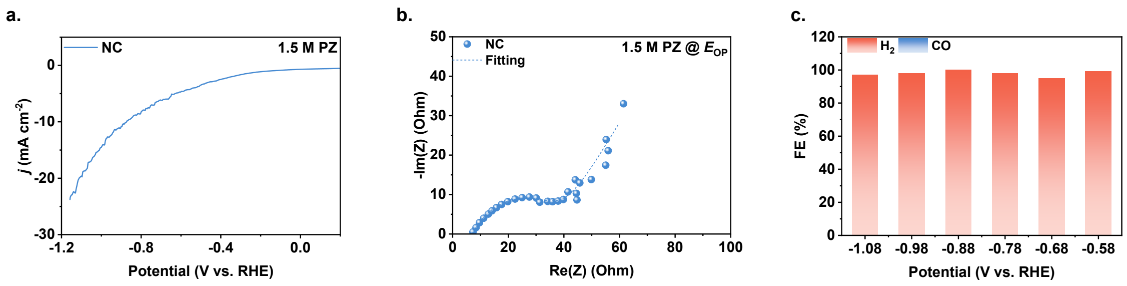

Fig. S20. Additional CO_2 electroreduction in 1.5 M PZ.

(a) LSV curves of NC in 1.5 M PZ with full CO_2 loading. (b) EIS and fit curves for three catalysts collected at E_{OP}. (c) FE_{H2} and FE_{CO} of NC.

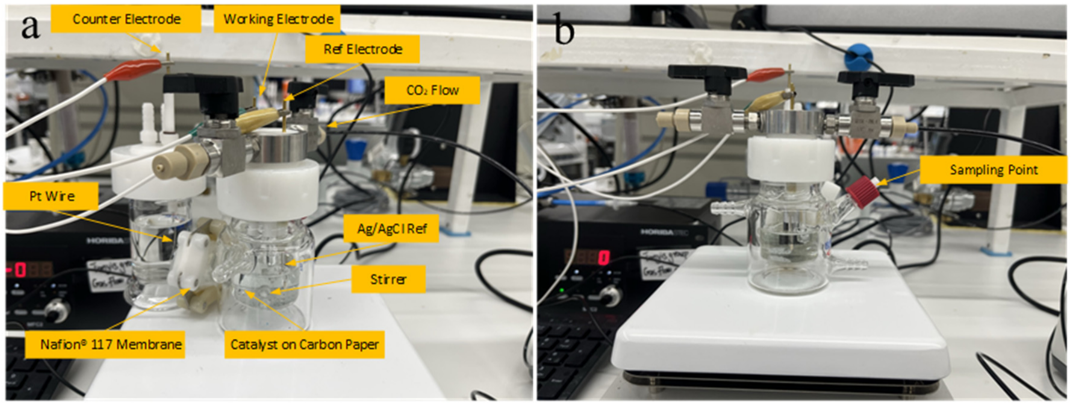

Fig. S21. H-cell used in electrochemical test.

(a) Front view of H-cell. (b) Side view of H-cell.

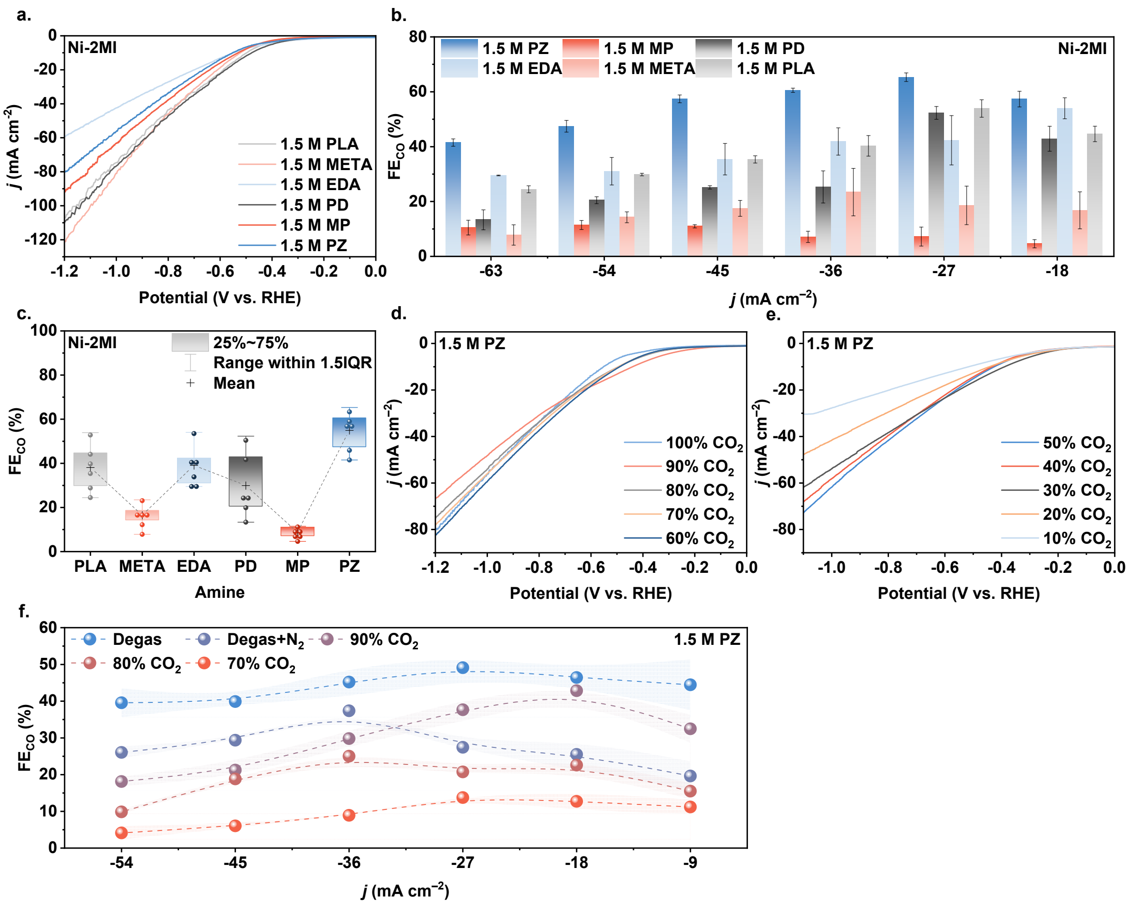

Fig. S22. Additional CO_2 electroreduction in six 1.5 M amine solutions at full and varying CO_2 loadings

(a) LSV curves of Ni–2MI in 1.5 M amines with full CO_2 loading. (b) FE_{CO} in six 1.5 M amine solutions. (c) Box plot of FE_{CO} in six 1.5 M amine solutions. The boxes represent the interquartile range (IQR), with the cross inside each box marking the median. Whiskers extend to the minimum and maximum values, and individual data points are shown as circles. (d–e) LSV curves in 1.5 M PZ with CO_2 loading from 60% to 100% (d) and from 10% to 50% (e). (f) FE_{CO} at different CO_2 loadings in 1.5 M PZ.

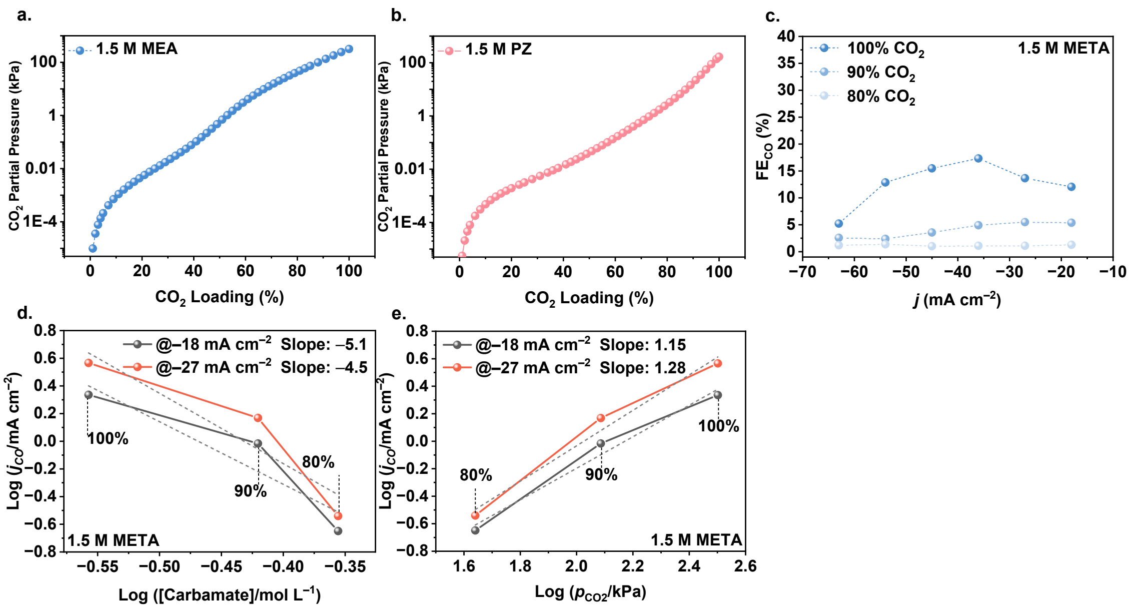

Fig. S23. Additional CO_2 electroreduction in 1.5 M META solution.

(a–b) Comparison of CO_2 partial pressure as a function of CO_2 loading in 1.5 M META (a) and 1.5 M PZ (b) solutions. The VLE for the CO_2-META/PZ system was calculated using the NRTL model (*14*). (c) FE_{CO} at different CO_2 loadings in 1.5 M META. (d–e) Logarithmic correlation between carbamate concentration and CO current density, where (d) represents varying carbamate concentrations by adjusting CO_2 loading, and (e) represents CO_2 partial pressure at different loading.

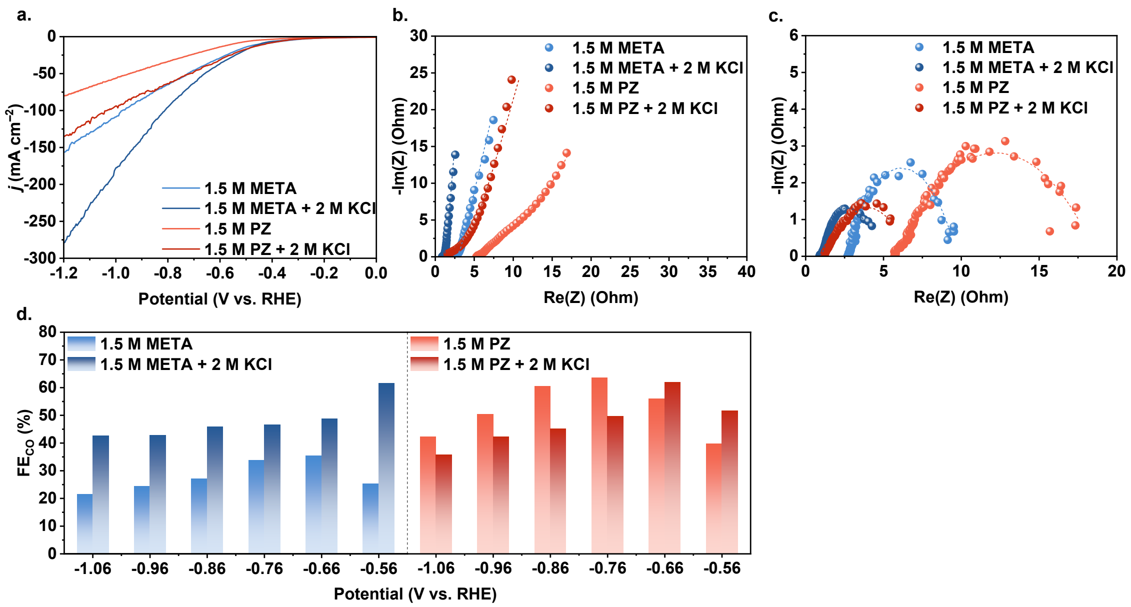

Fig. S24. Additional CO_2 Electroreduction in 1.5 M Amine Solutions with 2 M KCl.

(a) LSV curves of the Ni–2MI catalyst in 1.5 M PZ and META under fully CO_2-loaded conditions, with and without the addition of 2 M KCl. (b–c) EIS of the Ni–2MI catalyst in the four solutions, measured at both E_{op} (b) and open circuit potential (E_{ocp}) (c). (d) FE_{CO} in 1.5 M PZ and META solutions with full CO_2 loading, with and without the addition of 2 M KCl, under different applied potentials.

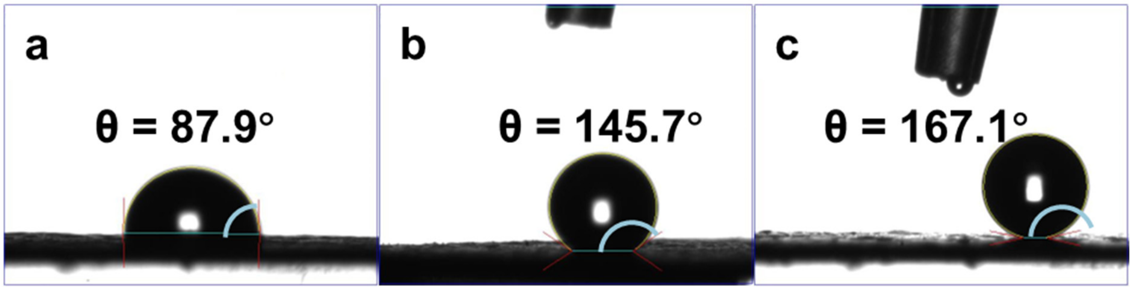

Fig. S25. Water angle measurements for substrates used in amine-CO_2 reduction microenvironment investigation.

(a) TGP-H-090 5% PTFE, (b) TGP-H-090 30% PTFE, and (c) TGP-H-090 30% PTFE with an additional 60% PTFE applied to the substrate.

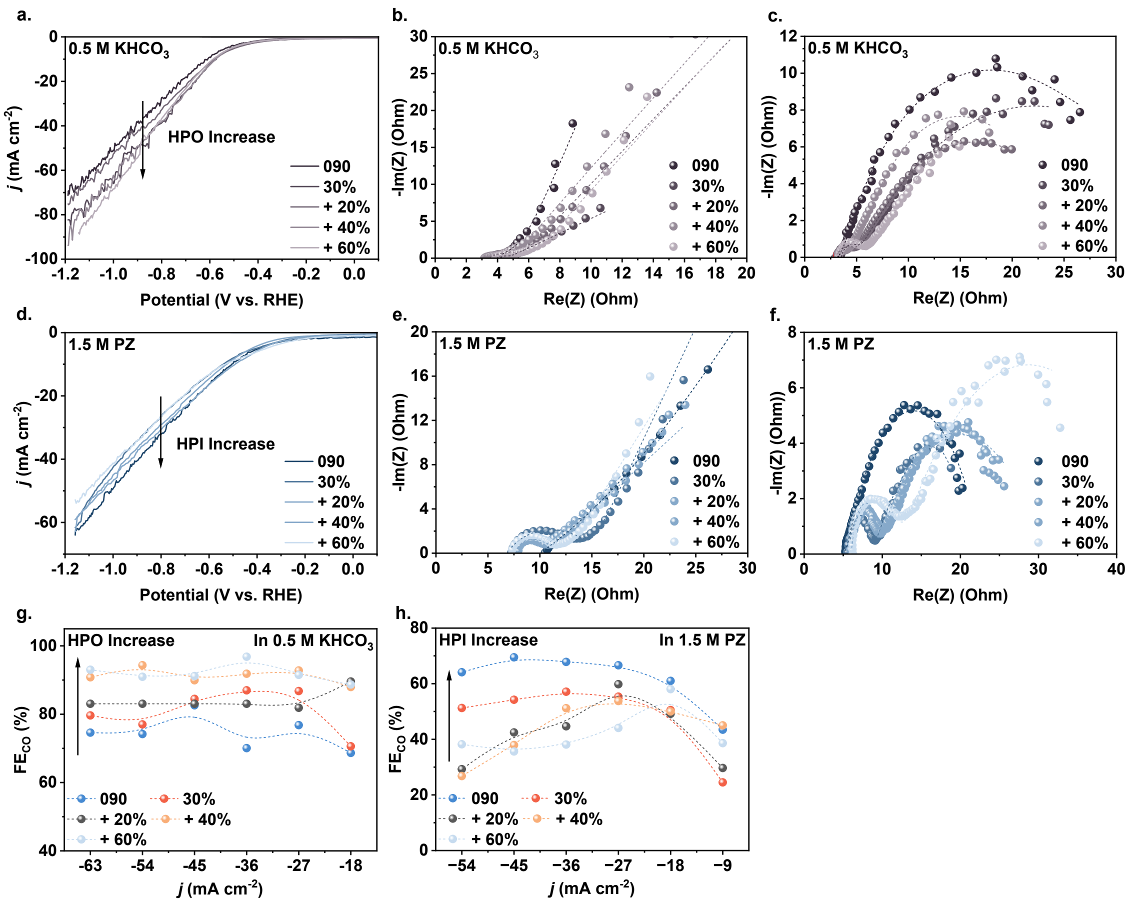

Fig. S26. Additional CO_2 electroreduction on PTFE-modified electrodes.

(a) LSV curves in 0.5 M $KHCO_3$ solutions under different PTFE content conditions. (b–c) EIS plots and their equivalent circuits in 0.5 M $KHCO_3$ under E_{ocp} (b) and E_{op} (c). (d) LSV curves in 1.5 M PZ solutions under different PTFE content conditions. (e–f) EIS plots and their equivalent circuits in 1.5 M PZ under E_{ocp} (e) and E_{op} (f). (g) FE_{CO} in the 1.5 M PZ solution. (h) FE_{CO} in the 0.5 M $KHCO_3$ solution.

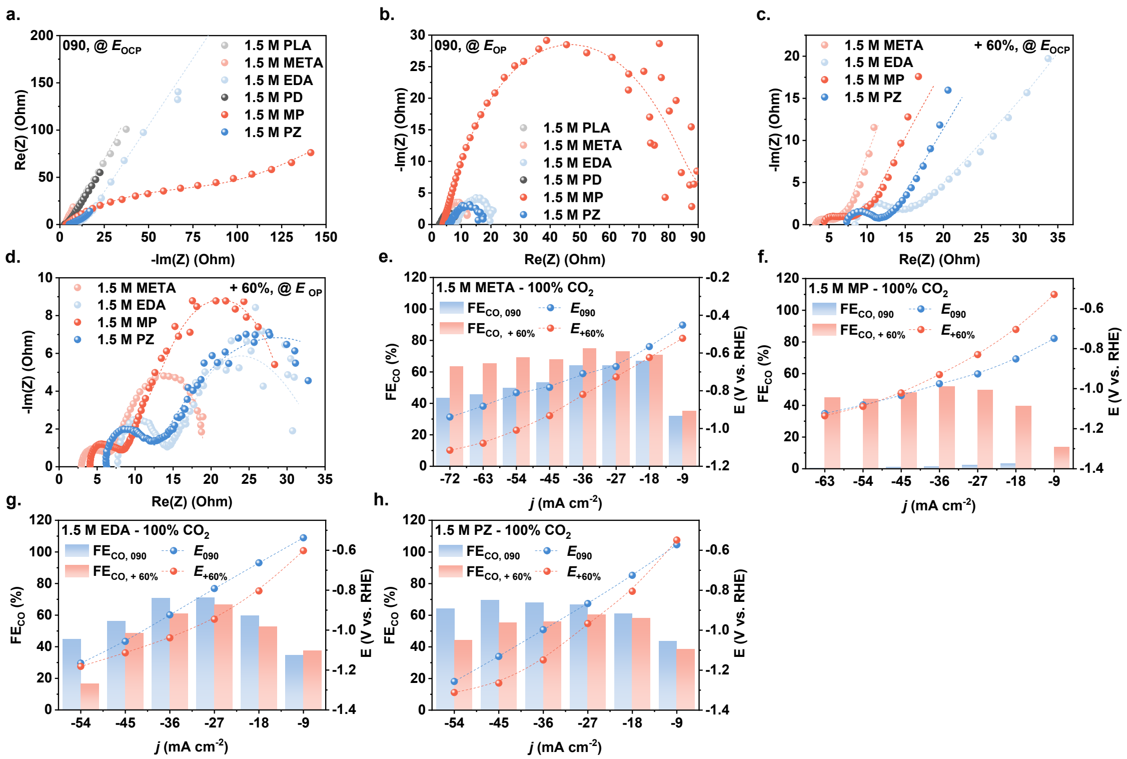

Fig. S27. Additional CO_2 electroreduction in six 1.5 M amines.

Impedance spectra for various amine solutions under different electrode configurations. (a) EIS of TGP-H-090 5% PTFE under E_{ocp} in six amines. (b) EIS of TGP-H-090 30% PTFE under E_{ocp} in four amines. (c) TGP-H-090 5% PTFE under E_{op}. (d) TGP-H-090 30% PTFE under E_{op}. (e–h) Fig. of FE_{CO} and the corresponding potentials in four amine solutions, using two different microenvironment electrode configurations. (e) 1.5 M META, (f) 1.5 M MP, (g)1.5 M EDA, and (h)1.5 M PZ with 100% CO_2 loading.

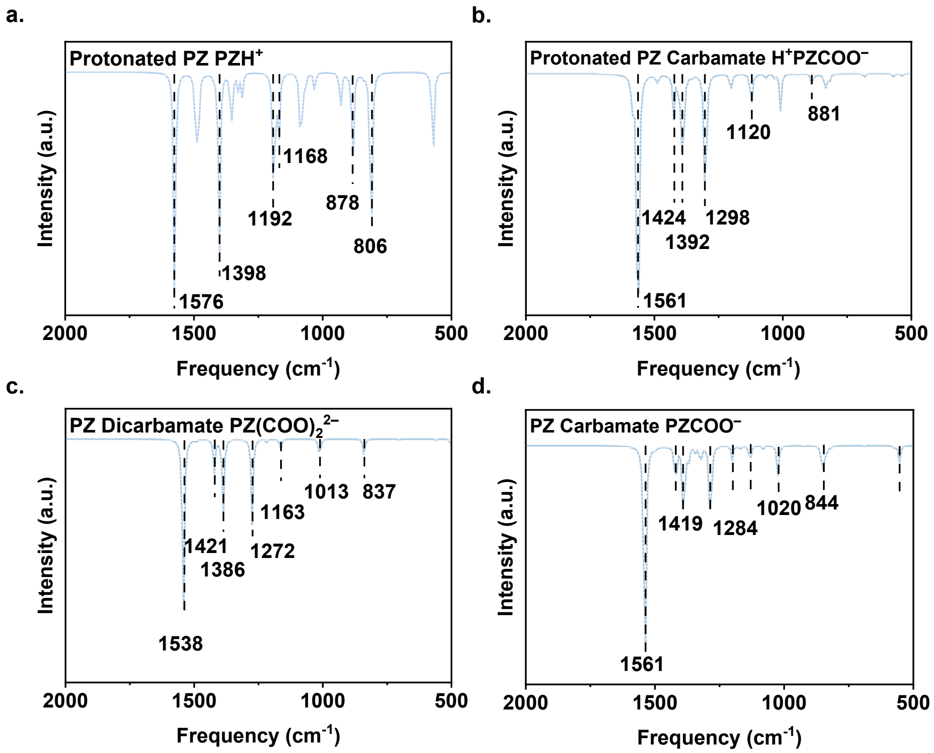

Fig. S28. Simulated IR spectra of PZ-CO_2 ion species.

(a) protonated PZ, (b) protonated PZ carbamate, (c) PZ dicarbamate, and (d) PZ carbamate, with distinct absorption peaks corresponding to their respective functional groups.

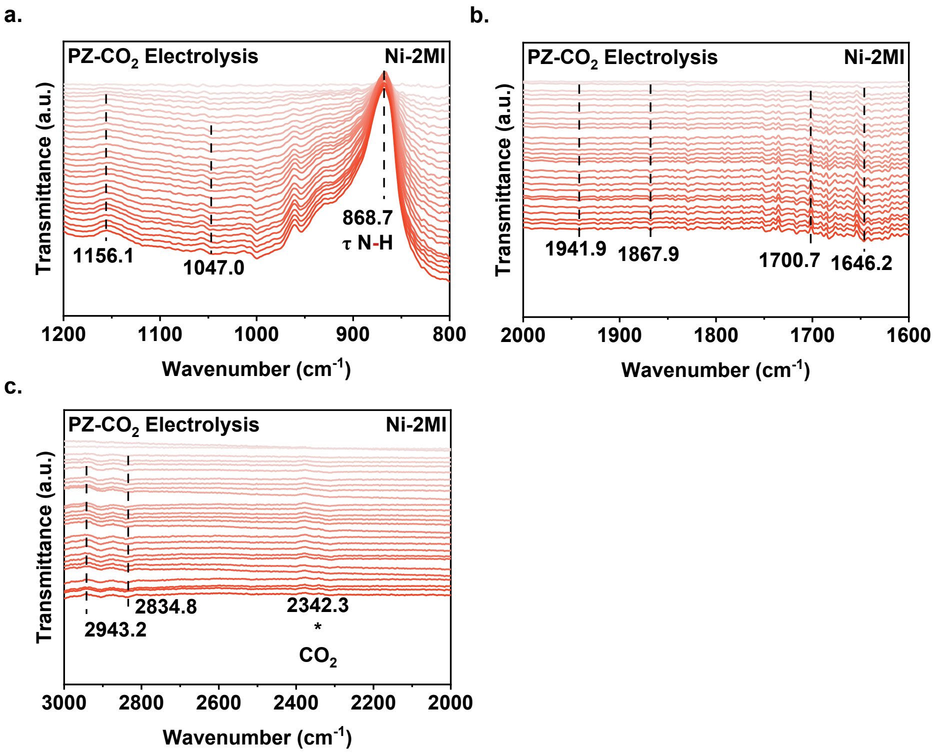

Fig. S29. In situ ATR–FTIR spectra in the electrolysis of 1.5 M PZ-captured CO_2 solution under applied potentials from 0 V to –2.5 V vs. RHE with Ni–2MI as the catalyst.

(a) 800-1200 cm^{-1}. (b) 1600-2000 cm^{-1}. (c) 2000-3000 cm^{-1}.

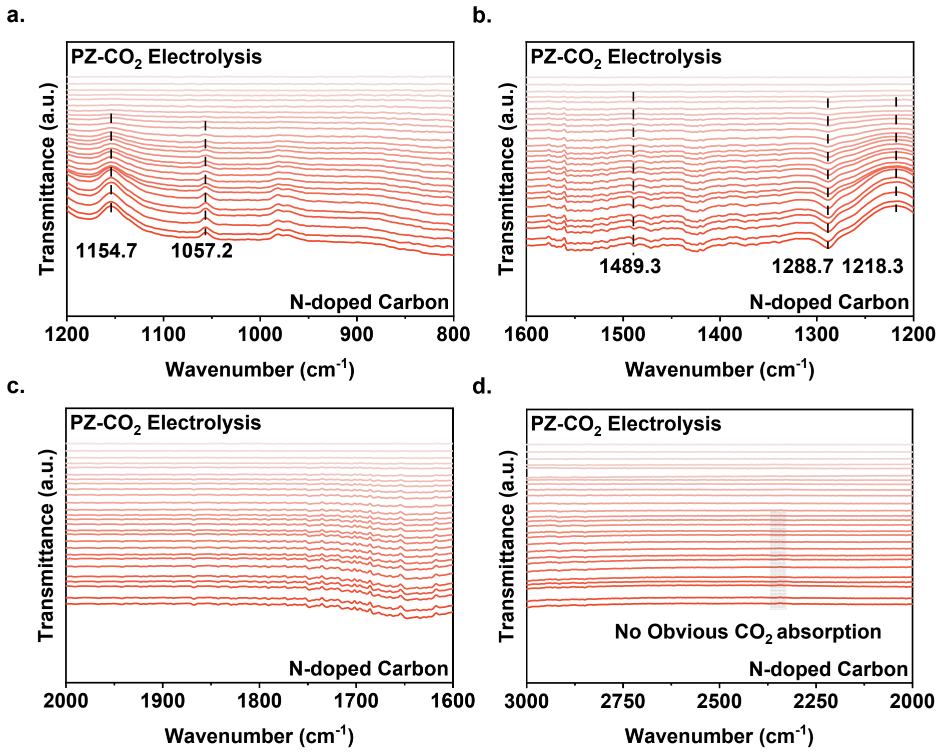

Fig. S30. In situ ATR–FTIR spectra in the electrolysis of 1.5 M PZ-captured CO_2 solution under applied potentials from 0 V to –2.5 V vs. RHE with N-doped carbon as the catalyst.

(a) 800-1200 cm^{-1}. (b) 1200-1600 cm^{-1}. (c) 1600-2000 cm^{-1}. (d) 2000-3000 cm^{-1}.

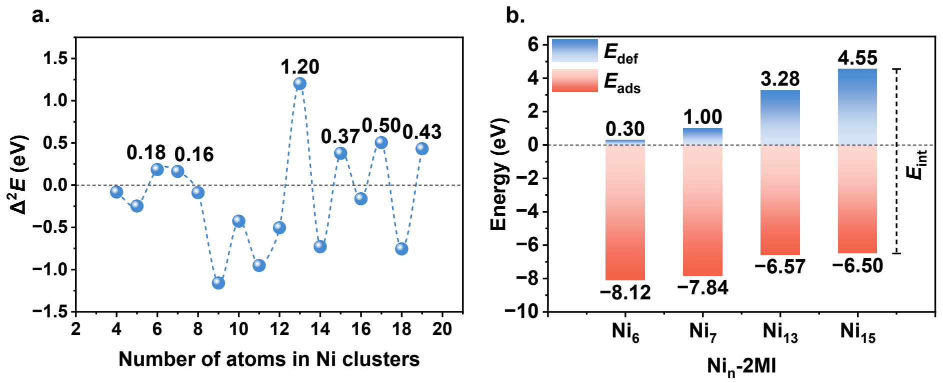

Fig. S31. Stability analysis of Ni_n clusters in the gas-phase and Ni_n clusters supported on N4-doped graphene

(a) Calculated $\Delta_2 E$ of Ni_n clusters (n = 4–19) in the gas phase. (b) Calculated E_{ads}, E_{int}, and E_{def} for Ni_6–, Ni_7–, Ni_{13}–, and Ni_{15}–2MI clusters supported on a graphene substrate.

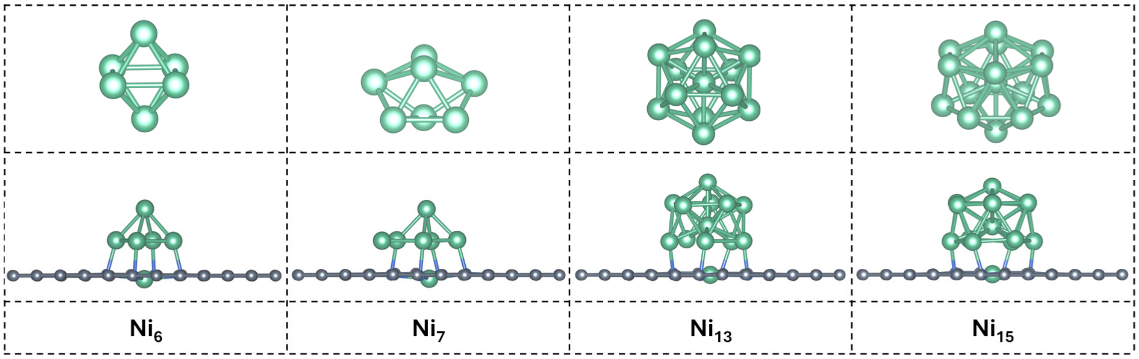

Fig. S32. Optimized structures of selected Ni_n clusters.

The optimized structures of Ni_6, Ni_7, Ni_{13}, and Ni_{15} clusters in gas-phase (top-panel) and the corresponding Ni_n cluster decorated N_4-doped graphene substrate substrates (bottom panel). These cluster sizes are considered because pronounced positive $\Delta_2 E$ values are observed for Ni_6, Ni_7, Ni_{13}, and Ni_{15}.

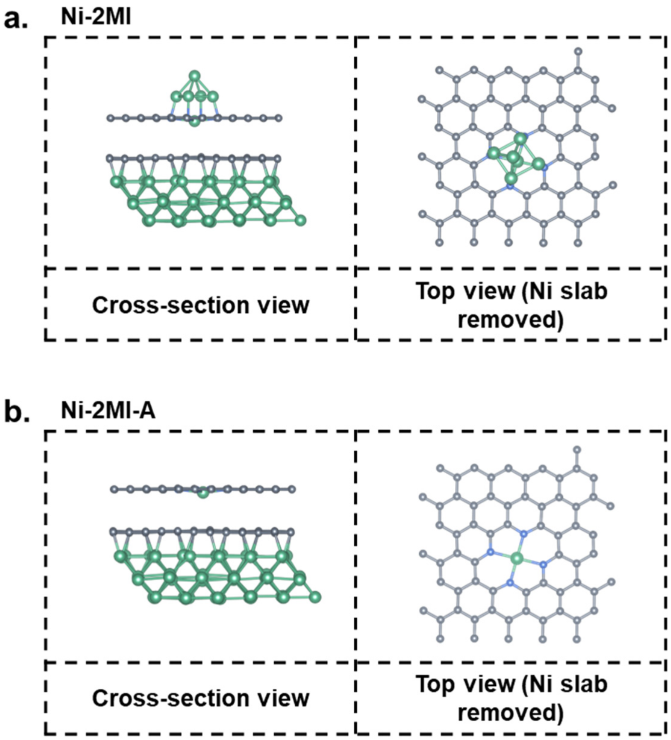

Fig. S33. Optimized structures of catalysts.

The optimized structures of (a) Ni–2MI and (b) Ni–2MI–A catalysts (cross-sectional and top views are displayed in left and right panels, respectively). Ball-and-stick model with colour code: blue, N; grey, C; green, Ni.

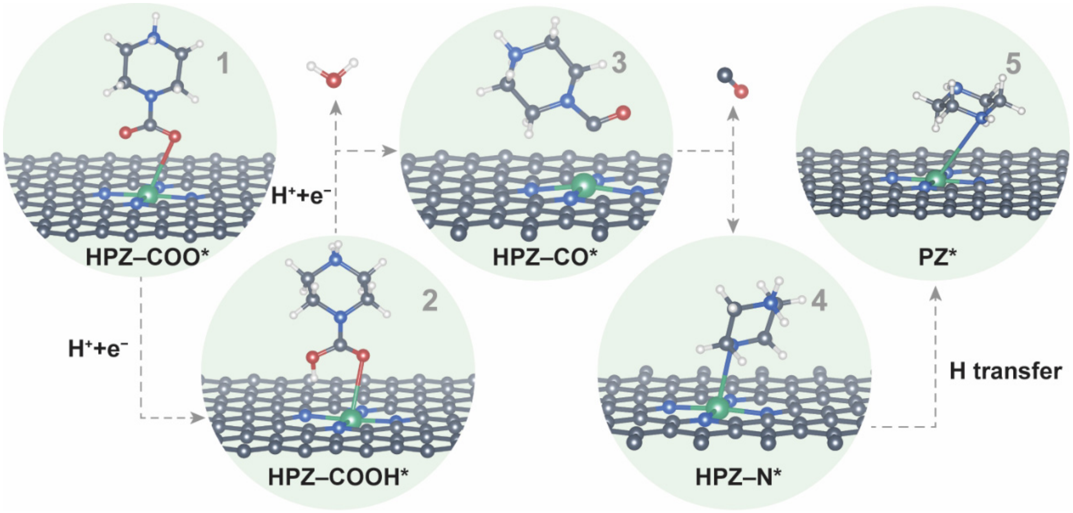

Fig. S34. Proposed reaction pathway for electrochemical CO_2 reduction of $H^+PZ–COO^-$ on Ni–2MI–A (For clarity, only the top layer is shown).

Ball-and-stick model with colour code: white, H; red, O; blue, N; grey, C; green, Ni.

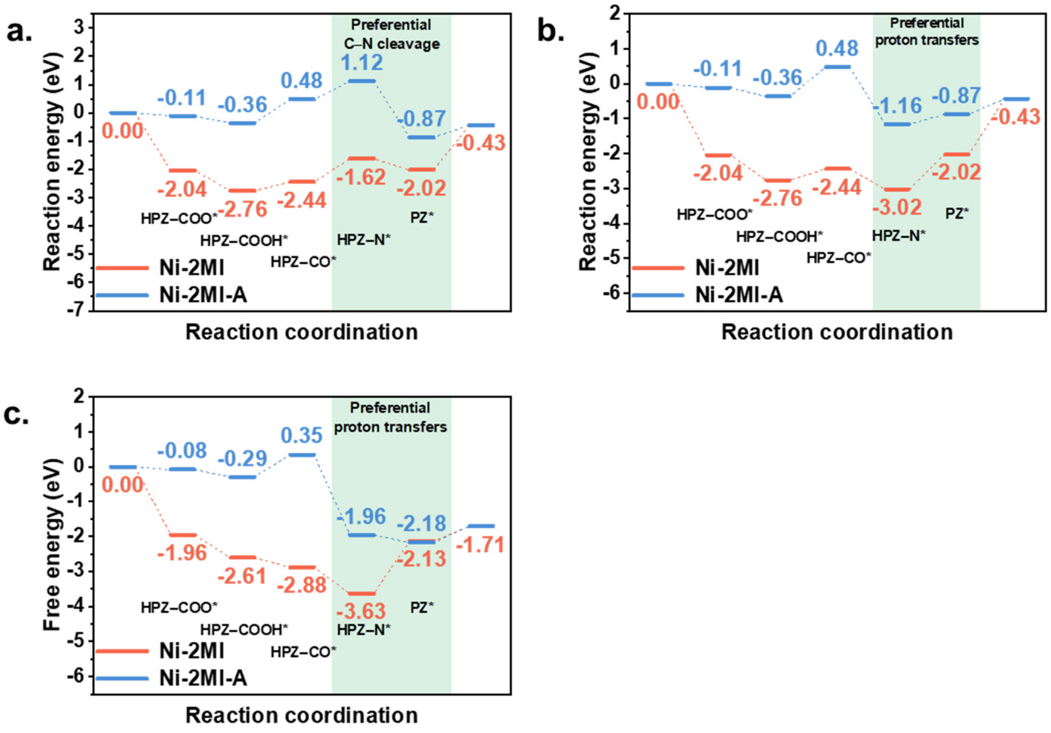

Fig. S35. Energies profiles along different reaction coordinates for reaction pathway.

(a, b) Reaction energy profile for PZ–CO_2 reduction via C–N cleavage and proton transfers pathways. (c) Free energy profile for PZ–CO_2 reduction via proton transfers pathway.

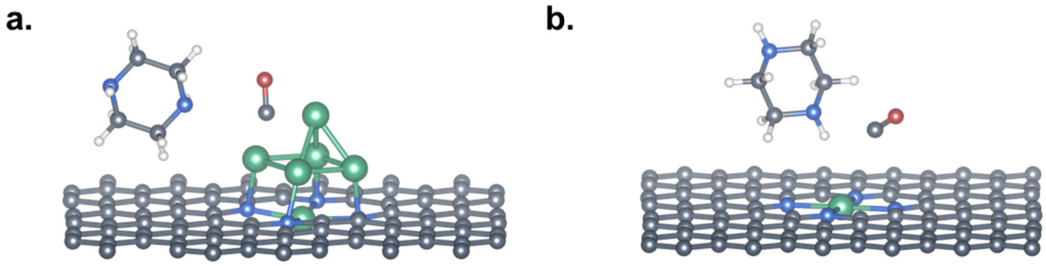

Fig. S36. Adsorption configurations of PZ–N*H + CO* (coordination 4) on two catalysts via proton transfers pathway.

The optimized structures of intermediates (PZ–N*H + CO*, coordination 4) via proton transfers pathway on (a) Ni–2MI and (b) Ni–2MI–A catalysts Ball-and-stick model with colour code: white, H; red, O; blue, N; grey, C; green, Ni.

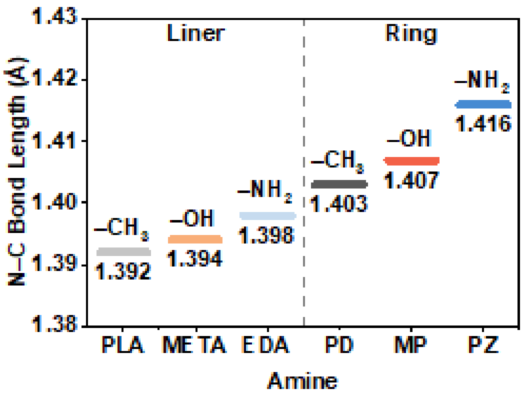

Fig. S37. N−C bond length of six amines.

The formed C−N bond lengths in both EDA and PZ are relatively longer in linear or cyclic structures, indicating that the C−N bonds are more prone to cleavage during the reduction process.

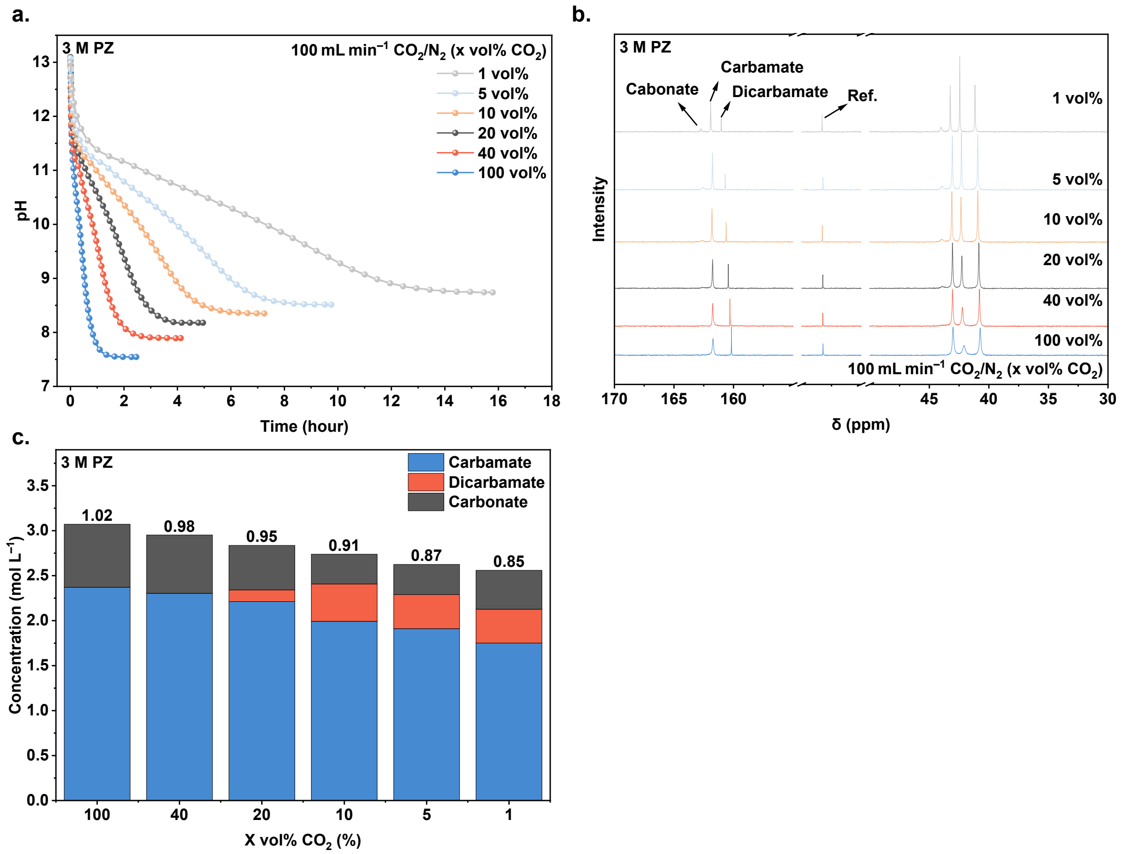

Fig. S38. Carbon capture and distribution analysis of 3 M PZ under dilute CO_2 source.

(a) pH profiles of 3 M PZ during CO_2 absorption from gas streams containing 1–100 vol% CO_2 (balanced with N_2) at a total flow rate of 100 mL min^{-1}. (b) Corresponding ^{13}C NMR spectra of PZ solutions sampled at saturation state under various CO_2 concentration. (c) Distribution of carbon species in solution, including carbamate, dicarbamate, and carbonate ions. The numerical values above each bar represent the total CO_2 loading under each condition, which are 1.02, 0.98, 0.95, 0.91, 0.87, and 0.85 mol CO_2 per mol PZ for 100, 40, 20, 10, 5, and 1 vol% CO_2 streams, respectively.

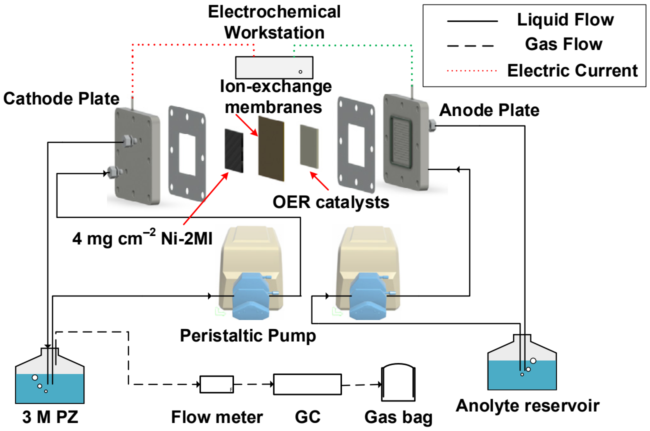

Fig. S39. Experiment set-up for PZ-CO_2 reduction in 1 cm^2 zero gap electrolyzer.

A zero gap electrolyzer with an active flow area of 1 cm^2 was employed for the electrochemical setup. A fixed volume of 3 M PZ solution was pre-saturated with CO_2 and continuously circulated at a controlled flow rate on the cathode side during operation. The cathode and anode compartments were separated by a specific ion exchange membrane. The anode side was loaded with the corresponding OER catalyst, and a dedicated anolyte was circulated throughout the process. Detailed experimental conditions and test parameters are summarized in Table S19. To determine the CO_2 loading in the PZ solution after a defined electrolysis period, the liquid samples were analysed following the ^{13}C NMR protocol. Gaseous products were monitored in real-time using online GC, and the gas stream was also collected in gas sampling bags for accumulated product quantification.

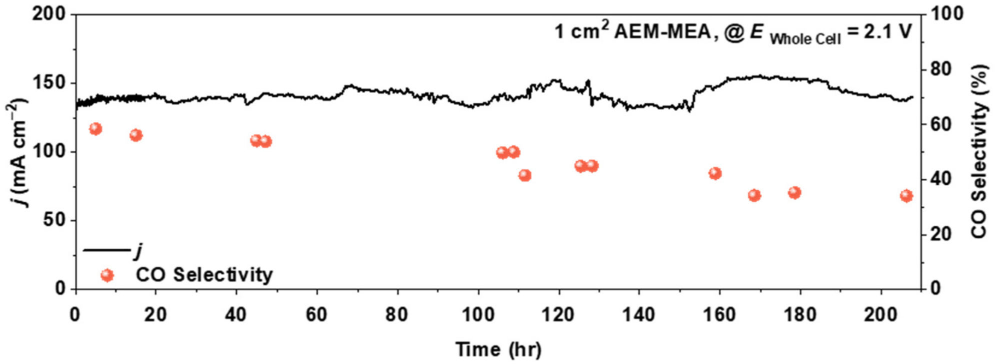

Fig. S40. Durability test (210 h) of 3 M PZ with saturated CO_2 under a cell voltage of 2.1 V in a 1 cm^2 AEM-MEA.

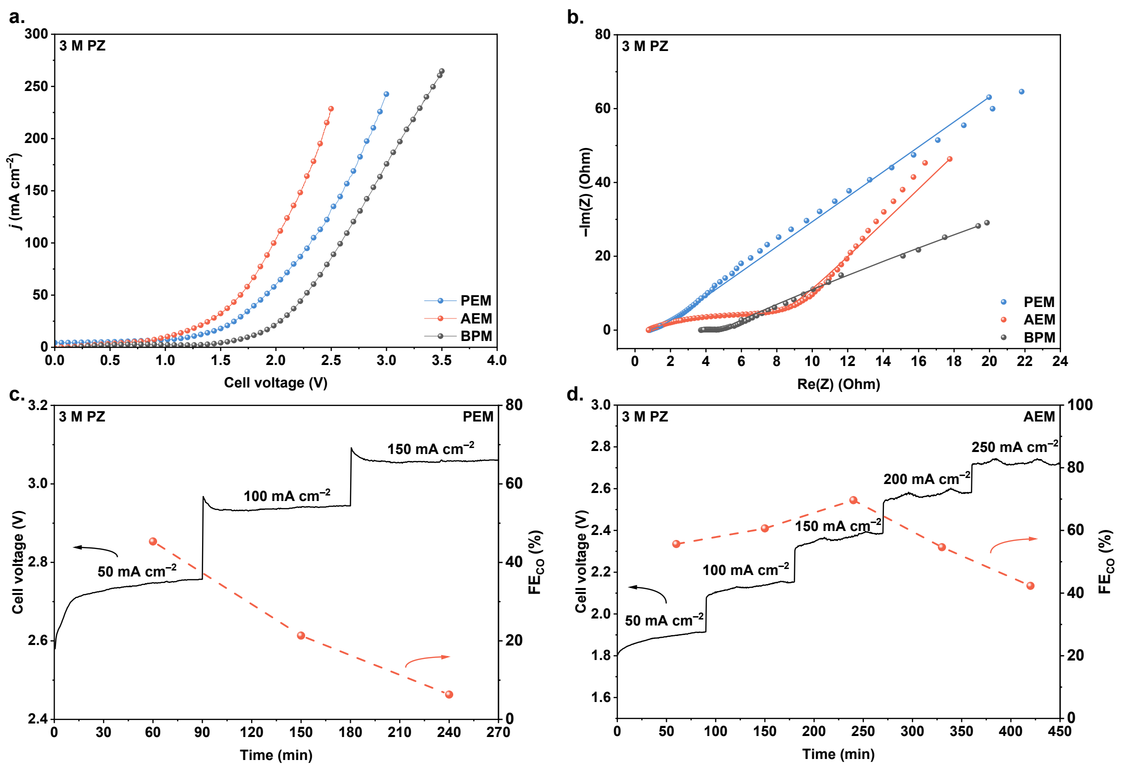

Fig. S41. Additional electrochemical data comparing the performance of PEM, AEM, and BPM in integrated CO_2 reduction in 3 M PZ a 1cm^2 MEA configuration.

(a) LSV curves of 3 M PZ using PEM, AEM, or BPM. (b) EIS in 3 M PZ under E_{ocp} using the three membrane configurations. (c, d) Cell voltage and FE_{CO} at different applied current densities using PEM (c) and AEM (d).

All measurements were conducted using 3 M PZ saturated with CO_2. The choice of membrane significantly influenced the electrochemical behaviour of the zero-gap electrolyser. From the LSV curves, it is evident that to achieve the same current density, the required cell voltage follows the order: BPM > PEM > AEM. The higher cell voltage observed with the BPM is attributed to the additional potential required to drive water dissociation at the membrane interface, resulting in a voltage gap of approximately ~0.83 V . This observation is consistent with the EIS results, where the R_s for PEM and AEM are 0.71 ohm and 0.92 ohm, respectively, while the R_s for BPM is significantly higher at 3.6 ohm. The FE_{CO}, measured at varying applied current densities, further highlights the impact of membrane selection. Under PEM operation, a relatively high FE_{CO} (~45.3%) is only observed at a low current density of 50 mA cm^{-2}. The AEM configuration achieves FE_{CO} > 60% across a wide current density range from −50 to −150 mA cm^{-2}, demonstrating superior performance for electrochemical CO_2 conversion under reactive capture conditions. It should be noted that FE_{CO} displays a volcano-type dependence on the effective operating potential, with an optimal window beyond which enhanced HER at higher cell voltages leads to a decline in CO selectivity.

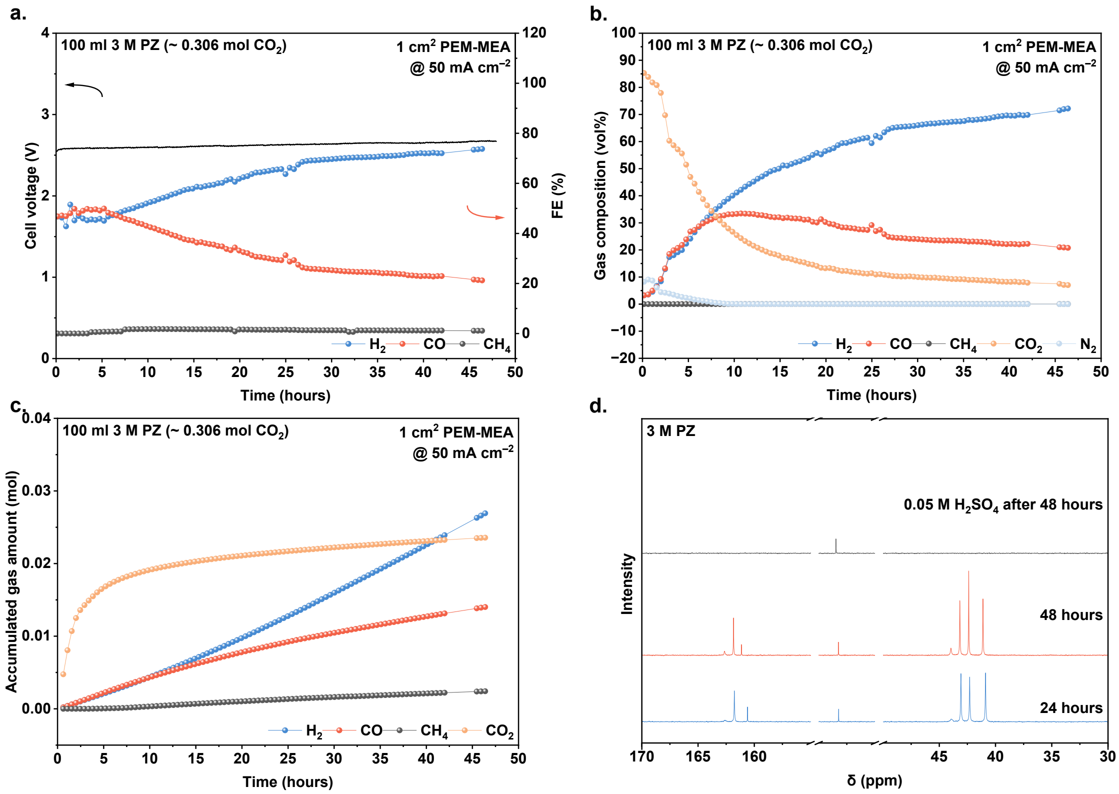

Fig. S42. Electrochemical performance of 3 M PZ using a PEM during continuous electrolysis starting from CO_2-saturated conditions at a fixed current density of 50 mA cm^{-2}.

(a) Time-dependent cell voltage, FE_{H2}, FE_{CO}, and FE_{CH4} during electrolysis. (b) Temporal evolution of gas composition in the product stream. (c) Accumulated amounts of gaseous products (CO_2, CO, CH_4) released from the 3 M PZ solution over time. (d) ^{13}C NMR spectra of 3 M PZ and 0.05 M H_2SO_4 samples collected at selected time points, showing changes in carbon species composition.

The CO_2 loading (α) values in the catholyte were determined to be 0.92, 0.88 mol CO_2 per mol PZ at 24 and 48 hours of electrolysis, respectively. These values closely matched the total amount of desorbed CO_2 measured by GC, indicating consistency between the two quantification methods. Furthermore, analysis of the anolyte (0.5 M H_2SO_4) after 48 hours confirmed the absence of detectable carbon crossover, suggesting that the use of the BPM does not result in significant carbon crossover.

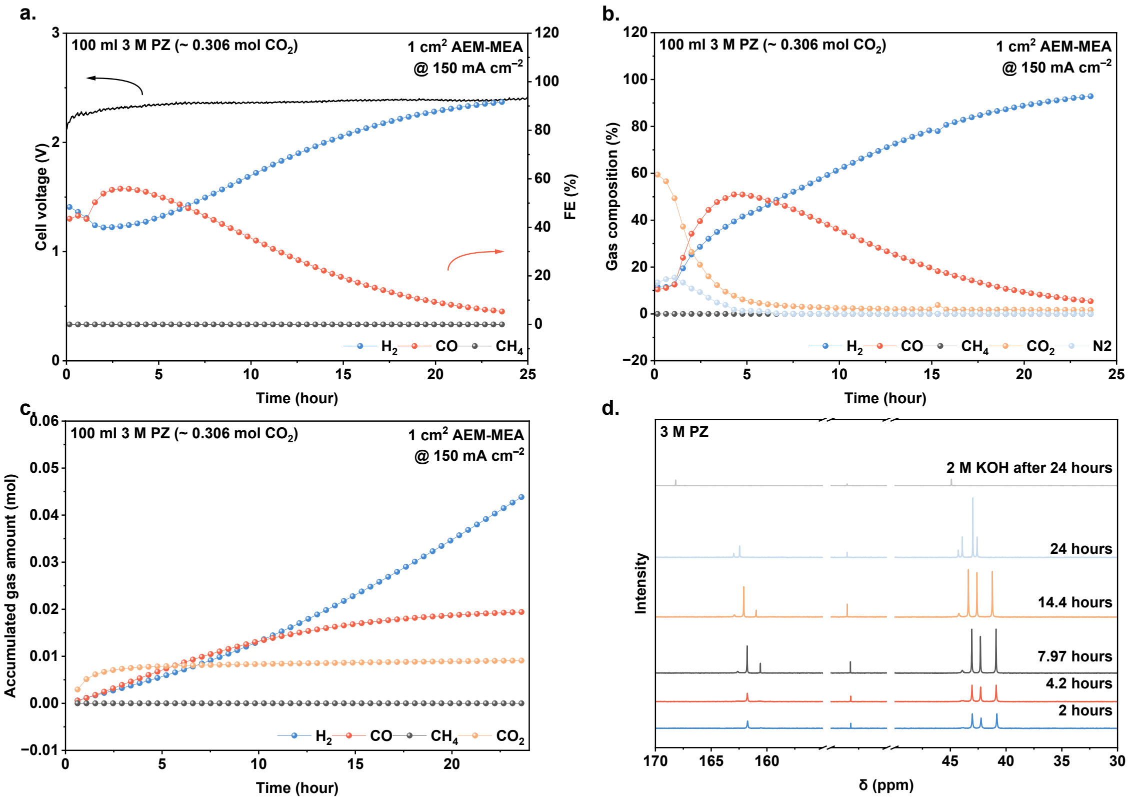

Fig. S43. Electrochemical performance of 3 M PZ using a AEM during continuous electrolysis starting from CO_2-saturated conditions (without CO_2 make-up) at a fixed current density of 150 mA cm^{-2}.

(a) Time-dependent cell voltage, FE_{H2}, FE_{CO}, and FE_{CH4} during electrolysis. (b) Temporal evolution of gas composition in the product stream. (c) Accumulated amounts of gaseous products (CO_2, CO, CH_4) released from the 3 M PZ solution over time. (d) ^{13}C NMR spectra of 3 M PZ samples collected at selected time points, showing changes in carbon species composition.

The CO_2 loading (α) values in the catholyte were determined to be 0.976, 0.925, 0.866, 0.782, and 0.687 mol CO_2 per mol PZ at 2, 4.2, 7.97, 14.4, and 24 hours of electrolysis, respectively. Analysis of the anolyte (2 M KOH) after 24 hours confirmed the presence of carbon-containing species, indicating carbon crossover. Importantly, negligible PZ transfer was detected in the anolyte, suggesting minimal amine migration across the membrane.

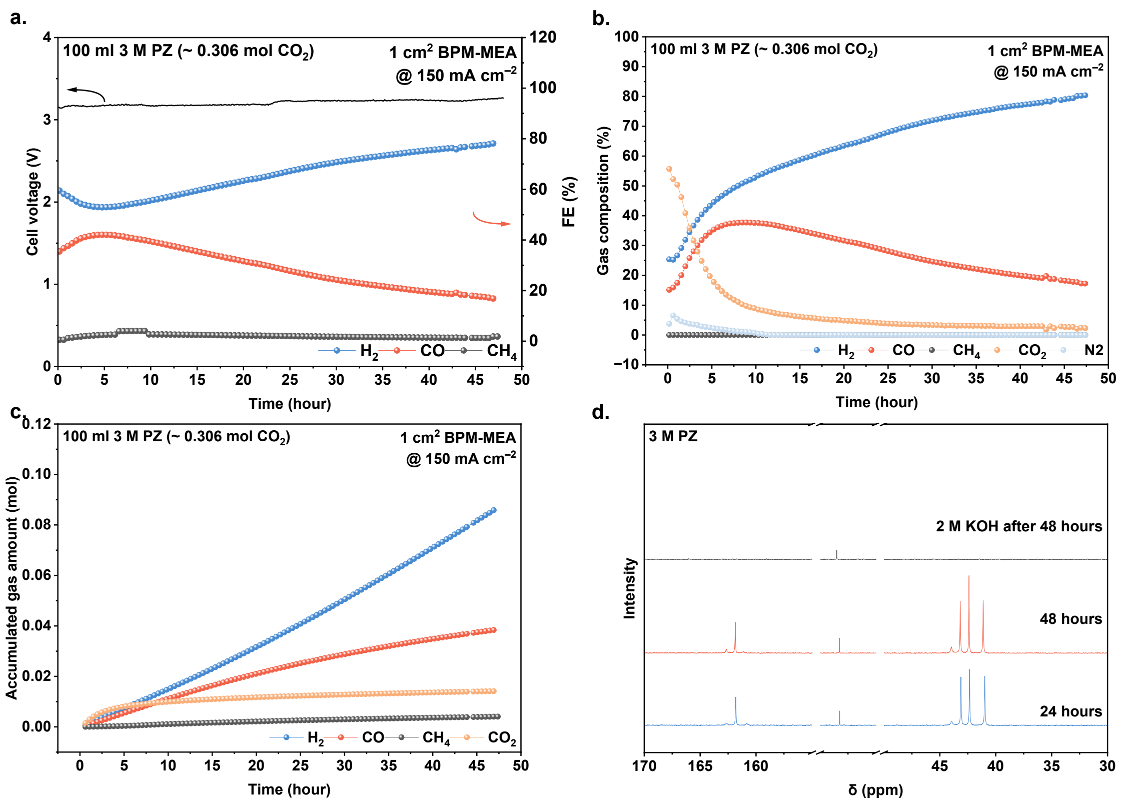

Fig. S44. Electrochemical performance of 3 M PZ using a BPM during continuous electrolysis starting from CO_2-saturated conditions (without CO_2 make-up) at a fixed current density of 150 mA cm^{-2}.

(a) Time-dependent cell voltage, FE_{H2}, FE_{CO}, and FE_{CH4} during electrolysis. (b) Temporal evolution of gas composition in the product stream. (c) Accumulated amounts of gaseous products (CO_2, CO, CH_4) released from the 3 M PZ solution over time. (d) ^{13}C NMR spectra of 3 M PZ and 2 M KOH samples collected at selected time points, showing changes in carbon species composition.

The CO_2 loading (α) values in the catholyte were determined to be 0.91, 0.83 mol CO_2 per mol PZ at 24 and 48 hours of electrolysis, respectively. These values closely matched the total amount of desorbed CO_2 measured by GC, indicating consistency between the two quantification methods. Furthermore, analysis of the anolyte (2 M KOH) after 48 hours confirmed the absence of detectable carbon crossover, suggesting that the use of the BPM does not result in significant carbon crossover.

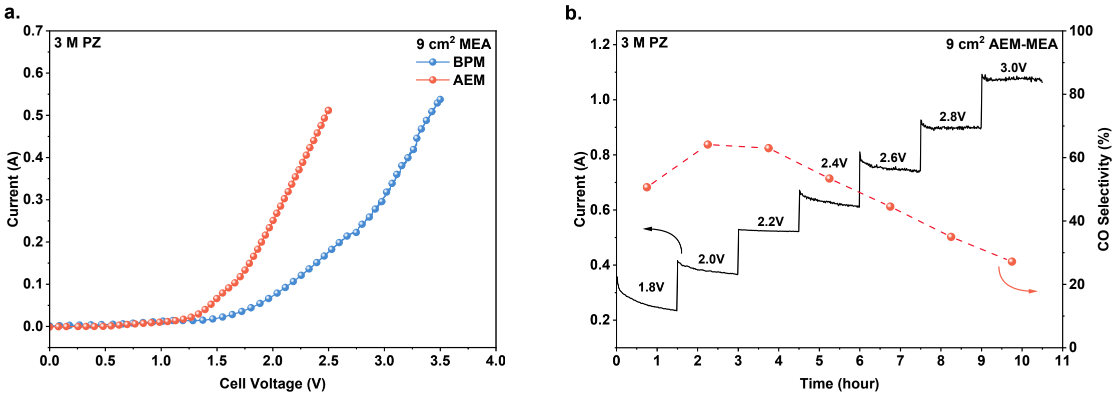

Fig. S45. Additional electrochemical data comparing the performance of AEM and BPM in integrated CO_2 reduction in 3 M PZ a 9cm^2 MEA configuration.

(a) LSV curves of 3 M PZ using AEM, or BPM in the 9 cm^2 MEA. (b) Cell voltage and FE_{CO} at different applied whole cell voltage using AEM.

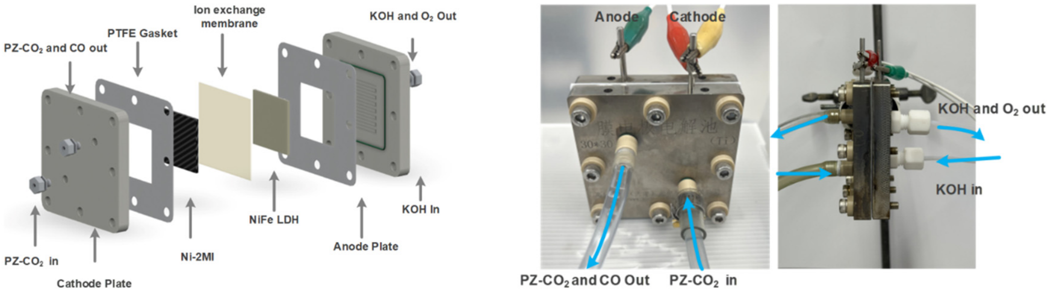

Fig. S46. Configuration of the 9 cm^2 MEA zero gap electrolyser.

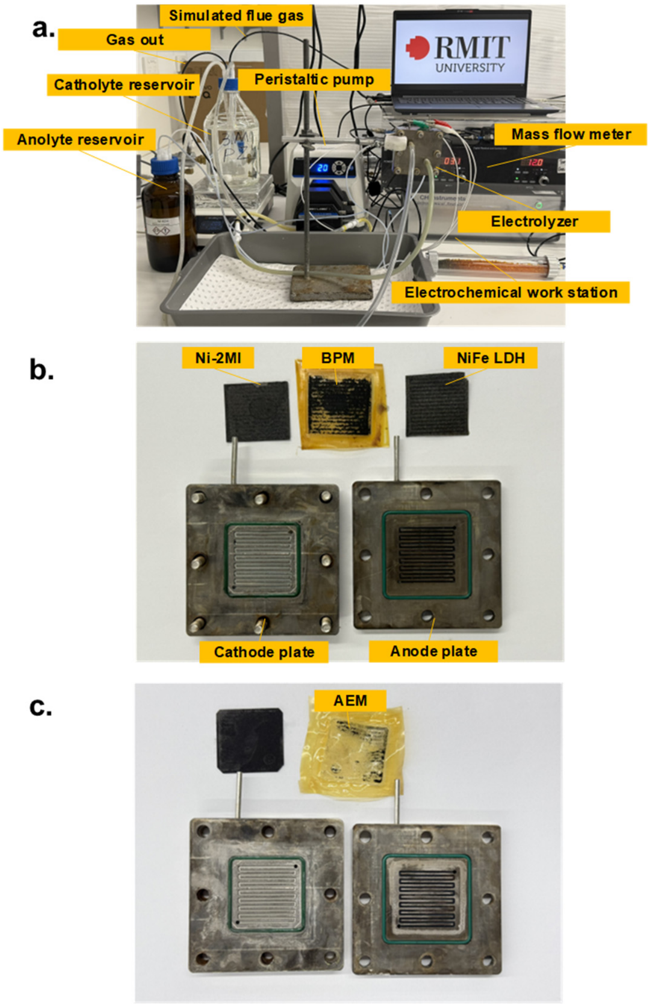

Fig. S47. Experimental Photos of the PZ-CO_2 reduction using the 9 cm^2 MEA zero-gap electrolyser.

(a) The reactive capture setup was constructed using a 9 cm^2 zero-gap electrolyser to integrate CO_2 capture and electrochemical conversion. (b, c) Representative images of the anode, cathode, membrane, and flow plates from the BPM (b) and AEM (c) setups after 150 hours of continuous operation.

Fig. (a) illustrates the setup for reactive CO_2 capture. Prior to electrolysis, approximately 1.8 L of PZ solution was saturated using a 20 vol% CO_2 simulated flue gas stream. During continuous electrolysis, a simulated flue gas stream (20 vol% CO_2 balanced with N_2) was continuously introduced into the catholyte reservoir, which also served as a bubble column for gas–liquid contact, at a flow rate of 12 mL min^{-1} for the BPM setup and 24 mL min^{-1} for the AEM setup, corresponding to CO_2 feed rates of 3 and 6 mL min^{-1}, respectively. Fig. b–c shows photographs of the cathode, anode, ion-exchange membrane, and flow plates in the BPM/AEM-based zero gap electrolyser after 150 hours of operation, with no observable signs of salt precipitation or component degradation.

Table S1. p*K*a of the Conjugate Acid of corresponding amines. All data were obtained from previous literatures (*33, 74*).

Amine	Conjugate Acid	pK_a of Conjugate Acid (pKa H)	pK_b of the amines
META	$METAH^+$	~9.5	5.5
MP	MPH^+	~8.4	5.6
PD	PDH^+	~11.2	2.8
PLA	$PLAH^+$	~10.6	3.4
PZ	PZH^+	~9.7	5.3
PZH^+	PZH_2^{2+}	~5.4	8.6
EDA	$EDAH^+$	~9.9	5.1
$EDAH^+$	PZH_2^{2+}	~6.8	7.2
OH^-*	H_2O	14	0
CO_3^{2-}*	HCO_3^-	~10.3	3.7

*Although OH^- and CO_3^{2-} are not amines, they are used here as reference Lewis bases due to their ability to donate electron pairs.

Table S2. η, σ, and pH of amine solutions before CO_2 capture and after CO_2 saturation (100% loading).

Solution	η (mPa s)	σ (mS cm^{-1})	pH
1.5 M META	1.48	1.32	12.01
1.5 M META-100% CO_2	1.71	46.12	7.65
1.5 M MP	1.60	0.23	11.59
1.5 M MP-100% CO_2	1.80	36.01	7.58
1.5 M PZ	1.60	0.86	12.33
1.5 M PZ-100% CO_2	2.24	15.44	7.42
1.5 M EDA	1.45	1.62	12.53
1.5 M EDA-100% CO_2	2.01	19.92	7.27
3 M PZ-100% CO_2	6.18	10.43	7.58

Table S3. Ionic species concentration in 1.5 M MEA and PLA at various CO_2 loadings.

	MEA				PLA			
CO_2 Loading (%)	100	90	80	70	100	90	80	70
α (mol CO_2 mol^{-1} amine)	0.78	0.74	0.68	0.66	0.94	0.86	0.71	0.67
$RNHCOO^-$ (mol L^{-1})	0.28	0.38	0.44	0.57	0.09	0.19	0.31	0.41
RNH_3^+/RNH_2 (mol L^{-1})	1.22	1.12	1.06	0.93	1.41	1.31	1.19	1.09
HCO_3^-/CO_3^{2-} (mol L^{-1})	0.89	0.72	0.58	0.42	1.30	1.22	0.98	0.65

Table S4. Ionic species concentration in 1.5 M MP and PD at various CO_2 loadings.

	MP				PD			
CO_2 Loading (%)	100	90	80	70	100	90	80	70
α (mol CO_2 mol^{-1} amine)	0.66	0.60	0.57	0.51	1.01	0.91	0.85	0.72
R_2NCOO^- (mol L^{-1})	0.33	0.37	0.48	0.52	0	0.05	0.11	0.15
$R_2NH_2^+/R_2NH$ (mol L^{-1})	1.17	1.12	1.02	0.98	1.5	1.45	1.39	1.35
HCO_3^-/CO_3^{2-} (mol L^{-1})	0.65	0.53	0.37	0.25	1.52	1.32	1.16	0.94

Table S5. Ionic species concentration in 1.5 M PZ at various CO_2 loadings.

CO_2 loading (%)	100	90	80	70	60
α (mol CO_2 mol^{-1} amine)	1.05	0.99	0.91	0.83	0.71
$PZCOO^-/H^+PZCOO^-$ (mol L^{-1})	1.09	0.99	0.84	0.76	0.65
$PZ(COO)_2^{2-}$ (mol L^{-1})	0	0.08	0.13	0.15	0.13
PZ/PZH^+ (mol L^{-1})	0.41	0.43	0.53	0.59	0.72
HCO_3^-/CO_3^{2-} (mol L^{-1})	0.47	0.33	0.28	0.18	0.17
CO_2 loading (mol L^{-1})	50	40	30	20	10
α (mol CO_2 mol^{-1} amine)	0.53	0.46	0.36	0.23	0.12
$PZCOO^-/H^+PZCOO^-$ (mol L^{-1})	0.56	0.46	0.39	0.28	0.13
$PZ(COO)_2^{2-}$ (mol L^{-1})	0.08	0.05	0.03	0	0
PZ/PZH^+ (mol L^{-1})	0.85	1.00	1.07	1.22	1.37
HCO_3^-/CO_3^{2-} (mol L^{-1})	0.16	0.13	0.07	0.06	0.05

Table S6. Ionic species concentration in 1.5 M EDA at various CO_2 loadings.

CO_2 Loading (%)	100	90	80	70
α (mol CO_2 mol^{-1} amine)	1.13	1.06	0.96	0.81
$EDACOO^{-}/H^{+}EDACOO^{-}$ (mol L^{-1})	1.35	1.28	1.16	0.97
$EDA(COO)_2^{2-}$ (mol L^{-1})	0	0.06	0.09	0.10
EDA/EDA^{+} (mol L^{-1})	0.15	0.15	0.24	0.43
HCO_3^{-}/CO_3^{2-} (mol L^{-1})	0.34	0.17	0.08	0.05

Table S7. Ionic species distributions in 1.5 M PZ solutions at different CO_2 loadings, obtained from Aspen Plus (v14.5) simulation**s** (*34-36*).

α (mol CO_2 mol^{-1} PZ)	CO_2 (%)	PZ (%)	$PZCOO^-$ (%)	PZH^+ (%)	$PZ(COO)_2^{2-}$ (%)	H^+PZCOO^- (%)	HCO_3^- (%)	CO_3^{2-} (%)
0.00	0.00	99.56	0.00	0.43	0.00	0.00	0.00	0.00
0.03	0.00	93.37	2.79	3.74	0.01	0.07	0.04	0.40
0.07	0.00	87.02	5.63	7.01	0.07	0.25	0.09	0.56
0.10	0.00	80.81	8.30	10.16	0.17	0.54	0.14	0.67
0.13	0.00	74.76	10.77	13.20	0.34	0.93	0.19	0.77
0.17	0.00	68.87	13.02	16.13	0.57	1.41	0.25	0.85
0.20	0.00	63.15	15.04	18.94	0.87	2.00	0.30	0.93
0.23	0.00	57.61	16.81	21.65	1.24	2.68	0.37	0.99
0.27	0.00	52.25	18.33	24.25	1.69	3.47	0.43	1.04
0.30	0.00	47.09	19.59	26.72	2.22	4.37	0.51	1.09
0.33	0.00	42.12	20.59	29.07	2.82	5.38	0.58	1.12
0.37	0.00	37.37	21.32	31.28	3.50	6.52	0.67	1.14
0.40	0.00	32.83	21.76	33.34	4.24	7.81	0.77	1.16
0.43	0.00	28.54	21.93	35.23	5.04	9.26	0.88	1.16
0.47	0.00	24.49	21.81	36.91	5.89	10.89	1.01	1.15
0.50	0.00	20.72	21.40	38.37	6.77	12.74	1.15	1.13
0.53	0.00	17.24	20.70	39.55	7.66	14.84	1.33	1.10
0.57	0.00	14.08	19.73	40.42	8.52	17.25	1.53	1.05
0.60	0.00	11.27	18.50	40.91	9.32	20.00	1.78	0.99
0.63	0.00	8.82	17.04	40.99	10.01	23.14	2.08	0.92
0.67	0.00	6.74	15.40	40.60	10.53	26.72	2.45	0.84
0.70	0.01	5.04	13.66	39.72	10.83	30.75	2.90	0.75
0.73	0.01	3.69	11.87	38.36	10.87	35.20	3.43	0.66
0.77	0.01	2.66	10.13	36.57	10.62	40.02	4.07	0.57
0.80	0.02	1.88	8.47	34.43	10.10	45.12	4.81	0.48
0.83	0.04	1.31	6.95	32.03	9.31	50.40	5.66	0.39
0.87	0.06	0.90	5.56	29.44	8.30	55.79	6.64	0.32
0.90	0.09	0.60	4.31	26.74	7.10	61.26	7.75	0.25
0.93	0.16	0.38	3.17	23.96	5.71	66.79	9.01	0.18
0.97	0.30	0.22	2.14	21.16	4.17	72.31	10.43	0.12
1.00	0.64	0.11	1.23	18.48	2.57	77.61	11.97	0.07

Table S8. Atomic and Mass composition of Ni–2MI, Ni–2MI–A, Ni-BDC, and Ni-BTC.

		C (%)	O (%)	N (%)	Ni (%)
Ni–2MI	Atomic (%)	72.8	16.0	9.0	2.2
	Mass (%)	63.1	18.5	9.1	9.3
Ni–2MI–A	Atomic (%)	85.6	2.7	10.3	1.4
	Mass (%)	79.1	3.3	11.1	6.5
Ni-BDC	Atomic (%)	81.9	14.6	2.7	0.8
	Mass (%)	75.7	18.0	2.9	3.4
Ni-BTC	Atomic (%)	81.3	12.5	2.4	3.9
	Mass (%)	67.9	13.9	2.3	15.9

Table S9. Table of the equivalent circuit (EC) and corresponding element diagram.

Equivalent circuit	Element diagram	No.
$R_s + \frac{C_{dl}}{R_{ct}}$	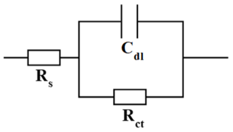	Cir-1
$R_s + \frac{C_{dl}}{R_{ct} + \frac{C_{trap}}{R_{trns} + W_d}}$	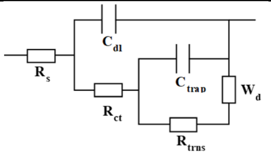	Cir-2

R represents a resistor with impedance $Z_R = R$. R_s is the internal resistance, also known as electrolyte solution resistance. R_{ct} is the charge transfer resistance at the surface of electrode, while R_{trns} is the electron transport resistance on the PTFE/carbon paper layer. C represents a capacitor with impedance $Z_C = \frac{1}{(j\omega C)^{\alpha}}$, where *j* is the imaginary unit, ω is the angular frequency, and α ($0 < \alpha \leq 1$) characterizes the constant phase element. C_{dl} is the double layer capacitance at the interface, and C_{trap} is the trap capacitance on the PTFE/ carbon layer. W_d represents Warburg diffusion element with impedance $Z_{W_d} = \sigma \frac{(1 - j)}{\sqrt{\omega}}$, where σ is the Warburg coefficient (*75*).

Table S10. EIS fitting parameters obtained from equivalent-circuit (Cir-1) analysis in fully CO_2-loaded 1.5 M PZ/META solutions, with and without the addition of 2 M KCl.

Solution	Measurement condition	R_s (ohm)	R_{ct} (ohm)
1.5 M PZ	OCP*	5.42	33.38
1.5 M PZ	OP*	5.87	12.7
1.5 M PZ + 2 M KCl	OCP	1.15	30.10
1.5 M PZ + 2 M KCl	OP	1.30	4.48
1.5 M META	OCP	2.69	58.75
1.5 M META	OP	2.64	6.03
1.5 M META + 2 M KCl	OCP	0.87	54.38
1.5 M META + 2 M KCl	OP	0.84	1.96

*OCP and OP denote open-circuit potential and operating potential, respectively.

Table S11. EIS fitting parameters obtained from equivalent-circuit analysis for electrodes with different PTFE contents in 0.5 M $KHCO_3$ under E_{op} condition.

PTFE content	R_s (ohm)	C_{trap} (F)	R_{trns} (ohm)	W_d (ohm)	C_{dl} (F)	R_{ct} (ohm)	EC
090	3.52	/	/	/	0.067	30.34	Cir-1
30%	3.18	0.00	1.24	13.52	0.13	6.73	Cir-2
+ 20%	2.84	0.01	1.46	9.01	0.06	12.51	Cir-2
+ 40%	2.93	0.01	1.83	12.38	0.09	8.00	Cir-2
+ 60%	3.95	0.07	1.43	8.82	0.03	13.18	Cir-2

Table S12. EIS fitting parameters obtained from equivalent-circuit analysis for electrodes with different PTFE contents in fully CO_2-loaded 1.5 M PZ solution under E_{op} condition.

PTFE content	R_s (ohm)	C_{trap} (F)	R_{trns} (ohm)	C_{dl} (F)	R_{ct} (ohm)	EC
090	9.50	/	/	0.02	15.83	Cir-1
30%	6.58	0.00	7.47	0.02	24.64	Cir-2
+ 20%	8.01	0.00	3.64	0.02	20.53	Cir-2
+ 40%	7.85	0.00	3.54	0.03	22.85	Cir-2
+ 60%	6.09	0.00	10.43	0.03	35.93	Cir-2

Table S13. EIS fitting parameters obtained from equivalent-circuit (Cir-1) analysis for TGP-H-090 5% PTFE carbon paper in six amine solutions.

Solution	Measurement condition	R_s (ohm)	R_{ct} (ohm)
1.5 M PLA	OCP	2.51	7624
1.5 M PLA	OP	2.25	3.88
1.5 M META	OCP	2.60	2667
1.5 M META	OP	2.79	8.02
1.5 M EDA	OCP	3.43	823
1.5 M EDA	OP	3.84	81.31
1.5 M PD	OCP	2.66	1797
1.5 M PD	OP	2.51	3.11
1.5 M MP	OCP	7.99	4982
1.5 M MP	OP	7.69	13.4

Table S14. EIS fitting parameters obtained from equivalent-circuit (Cir-2) analysis for TGP-H-090 carbon paper with 30% PTFE and an additional 60% PTFE coating in six amine solutions.

Solution	Measurement condition	R_s (ohm)	R_{ct} (ohm)
1.5 M META	OCP	2.90	1.75×10^{45}
1.5 M META	OP	3.02	2.63
1.5 M MP	OCP	3.03	1.75×10^{22}
1.5 M MP	OP	3.95	3.84
1.5 M EDA	OCP	8.14	7075
1.5 M EDA	OP	7.88	48.06
1.5 M PZ	OCP	6.80	2.62×10^{16}
1.5 M PZ	OP	7.68	6.90

Table S15. Wavenumber ranges and corresponding functional group vibrations identified in the IR spectra of CO_2 adsorption species.

Wave number range (cm^{-1})	Group
844−881	Twisting vibration of N−H
1272−1298	Stretching vibration of COO^-
1386−1424	Asymmetric stretching vibration of C−N (in the six-membered ring)
1360−1392	Symmetric stretching vibrations of the N−COO^-
1561−1571	Bending of NH^{2+}

Table S16. Reaction pathway and description of PZ–CO_2 reduction.

Step	Reaction	Description
1	HPZ–COO + * → HPZ–COO*	Adsorption of HPZ–COO onto the catalyst surface.
2	HPZ–COO* + H^+ + e^- → HPZ–COOH*	Proton-coupled electron transfer (PCET) forming the carboxyl intermediate (HPZ–COOH*)
3	HPZ–COOH* + H^+ + e^- → HPZ–CO* + H_2O	Decarboxylation (C–O bond cleavage) via PCET to form HPZ–CO*
4	HPZ–CO* → HPZ–N* + CO	C–N bond cleavage leading to CO desorption
4*	HPZ–CO* → PZ–N*H –CO*	Intermolecular proton transfer facilitates the formation of PZ
5	HPZ–N* → PZ*	Intramolecular proton transfer.

* Proton transfer pathway.

The intermediates in the reaction pathway are highlighted in bold, and the free and reaction energy is calculated based on these intermediates.

Table S17. Gibbs free energy change for PZ-CO_2 reduction.

Step	Gibbs free energy change
1	$\Delta G_1 = G_{HPZ–COO*} – G_{HPZ–COO} – G_*$
2	$\Delta G_2 = G_{HPZ–COOH*} – G_{HPZ–COO*} – ½\ G_{H2} + eU – \Delta G_{H+}(pH)$
3	$\Delta G_3 = G_{HPZ–CO*} – G_{HPZ–COOH*} + G_{H2O} – ½\ G_{H2} + eU – \Delta G_{H+}(pH)$
4	$\Delta G_4 = G_{HPZ–N*} + G_{CO} – \mathrm{G}_{HPZ–CO*}$
5	$\Delta G_5 = G_{PZ*} – G_{HPZ–N*}$
6	$\Delta G_6 = G_{PZ} + G_* – G_{PZ*}$

Here, U denotes the electrode potential referenced to the normal hydrogen electrode (NHE) under standard hydrogen electrode (SHE) conditions. The free energy correction for protons at a non-zero pH is determined using the Nernst Eq. (Eq. 27).

$$\Delta G_{H^+}(\mathrm{pH}) = –k_B T \ln(10) \times \mathrm{pH} \tag{S27}$$

In this work, a pH value of 7.4 was employed, corresponding to approximately 100% CO_2 loading.

Table S18. The amine carbamate formation energies and the molecular configurations. The values are determined via DFT calculations.

Name	Amines	Carbamates	$E_{formation}$ (kJ mol^{-1})
META	HO–CH_2–CH_2–NH_2	HO–CH_2–CH_2–NH–COO^-	–58.98
MP	O–(CH_2–CH_2)$_2$–NH	O–(CH_2–CH_2)$_2$–N–COO^-	–50.20
PLA	CH_3–CH_2–CH_2–NH_2	CH_3–CH_2–CH_2–NH–COO^-	–47.94
PD	CH_2–(CH_2–CH_2)$_2$–NH	CH_2–(CH_2–CH_2)$_2$–N–COO^-	–68.08
PZ	HN–(CH_2–CH_2)$_2$–NH	HN–(CH_2–CH_2)$_2$–N–COO^-	–63.73
EDA	H_2N–CH_2–CH_2–NH_2	H_2N–CH_2–CH_2–NH–COO^-	–66.26

Table S19. Experimental configurations for different ion exchange membranes used in the zero-gap electrolyser setup.

Parameter	PEM	AEM	BPM
Ion transport	Transfers H^+ from the anode to the cathode.	Transfers OH^-, HCO_3^-, and CO_3^{2-} from the cathode to the anode	Facilitates water dissociation, generating H^+ (to cathode) and OH^- (to anode)
Cathode	090 5% PTFE	060	090 5% PTFE
Catholyte flow rate (ml min^{-1})	60	30	30
Anode	IrO_2/Ti Flet	NiFe LDH	NiFe LDH
Anolyte	0.05 M H_2SO_4	2 M KOH	2 M KOH
Anolyte flow rate (ml min^{-1})	8	16	16
Set current (mA cm^{-2})	50	150	150

Table S20. Comparative analysis of reaction mechanisms, technological costs, and application scenarios across diverse carbon utilization pathways.

Carbon capture and utilization routes	Reaction mechanism	Technological products cost (GJ or US$ per ton of CO)	Technology readiness level (TRL) and potential retrofitting plant	Reference
Amine absorption and subsequent electrolysis. Based on PZ	One-step interface liquid carbamate reduction mechanism. $H^+PZCOO^- + H_2O + 2e^- \rightarrow CO + PZ + 2OH^-$	Best in all the reported capture and electrolysis studies. ~53 US$ per ton of CO	TRL of 9. Retrofitting Case Example: Hunter Power Plant and Boundary Dam	This work
Amine absorption and subsequent electrolysis. Based on presumable amines (MEA, TREA)	Two-steps interface gas-phase CO_2 reduction mechanism. $HCO_3^- + H^+ \rightarrow CO_2 + H_2O$ $CO_2 + H_2O + 2e^- \rightarrow CO + 2OH^-$	~78−103 GJ per ton of CO	TRL of 9. Retrofitting Case Example: Hunter Power Plant and Boundary Dam	*(5, 8, 61)*
Bi(carbonate) absorption and subsequent electrolysis. Based on KOH/K_2CO_3	Two-steps interface gas-phase CO_2 reduction mechanism. $HCO_3^- + H^+ \rightarrow CO_2 + H_2O$ $CO_2 + H_2O + 2e^- \rightarrow CO + 2OH^-$	~90 GJ per ton of CO	TRL of 9. (Limited Capture Rate) Retrofitting Case Example: Enid Fertilizer	*(76, 77) (29)*
Amino acid salt absorption and subsequent electrolysis. Based on potassium glycinate (K-GLY)	Two-steps interface gas-phase CO_2 reduction mechanism. $HCO_3^- + H^+ \rightarrow CO_2 + H_2O$ $CO_2 + H_2O + 2e^- \rightarrow CO + 2OH^-$	~105 GJ per ton of CO	TRL of 4−5. Challenging to retrofit existing carbon capture facilities due to low TRL.	*(28)*
Broad but isolated gas-phase CO_2 electrolysis route.	One-step gas-phase CO_2 reduction mechanism. $CO_2 + H_2O + 2e^- \rightarrow CO + 2OH^-$	~72 GJ per ton of CO	TRL of 3−6. Challenging to deploy further to KW/MW scale.	*(30, 62, 78, 79)*
Reverse water-gas shift (RWGS) route	One-step gas-phase CO_2 reduction mechanism. $H_2 + CO_2 \rightarrow CO + H_2O$	~72 GJ per ton of CO	TRL of 9. Well-established but challenging to decarbonization and couple with renewable energy.	*(80)*

Data S1. Experimental data for CO_2 capture and ICCE performance. Excel file containing the data used to generate the figures in the main text and Supplementary Materials.

REFERENCES

1. X. Lan, P. Tans, K. W. Thoning, Trends in globally-averaged CO_2 determined from NOAA Global Monitoring Laboratory measurements (2025); 10.15138/9N0H-ZH07.

2. Y. Zhong, Z. Zheng, D. Hao, H. Jin, X. Zheng, Y. Li, H. Yu, B. Jia, T. Ma, P. Li, Electrochemically integrated carbon capture and utilization. *ACS Mater. Lett.* **7**, 3952–3973 (2025).

3. L. Chen, G. Msigwa, M. Yang, A. I. Osman, S. Fawzy, D. W. Rooney, P.-S. Yap, Strategies to achieve a carbon neutral society: A review. *Environ. Chem. Lett.* **20**, 2277–2310 (2022).

4. F. Meng, Y. Meng, T. Ju, S. Han, L. Lin, J. Jiang, Research progress of aqueous amine solution for CO_2 capture: A review. *Renew. Sustain. Energy Rev.* **168**, 112902 (2022).

5. G. Lee, Y. C. Li, J.-Y. Kim, T. Peng, D.-H. Nam, A. Sedighian Rasouli, F. Li, M. Luo, A. H. Ip, Y.-C. Joo, E. H. Sargent, Electrochemical upgrade of CO_2 from amine capture solution. *Nat. Energy* **6**, 46–53 (2021).

6. L. Chen, F. Li, Y. Zhang, C. L. Bentley, M. Horne, A. M. Bond, J. Zhang, Electrochemical reduction of carbon dioxide in a monoethanolamine capture medium. *ChemSusChem* **10**, 4109–4118 (2017).

7. X. Y. D. Soo, J. J. C. Lee, W.-Y. Wu, L. Tao, C. Wang, Q. Zhu, J. Bu, Advancements in CO_2 capture by absorption and adsorption: A comprehensive review. *J. CO_2 Util.* **81**, 102727 (2024).

8. K. Shen, D. Cheng, E. Reyes-Lopez, J. Jang, P. Sautet, C. G. Morales-Guio, On the origin of carbon sources in the electrochemical upgrade of CO_2 from carbon capture solutions. *Joule* **7**, 1260–1276 (2023).

9. G. Leverick, E. M. Bernhardt, A. I. Ismail, J. H. Law, A. Arifutzzaman, M. K. Aroua, B. M. Gallant, Uncovering the active species in amine-mediated CO_2 reduction to CO on Ag. *ACS Catal.* **13**, 12322–12337 (2023).

10. A. Parkin, I. D. H. Oswald, S. Parsons, Structures of piperazine, piperidine and morpholine. *Acta Crystallogr. Sect. B Struct. Sci.* **60**, 219–227 (2004).

11. B. Yu, H. Yu, K. Li, Q. Yang, R. Zhang, L. Li, Z. Chen, Characterisation and kinetic study of carbon dioxide absorption by an aqueous diamine solution. *Appl. Energy* **208**, 1308–1317 (2017).

12. S. Wada, T. Kushida, H. Itagaki, T. Shibue, H. Kadowaki, J. Arakawa, Y. Furukawa, ^{13}C NMR study on carbamate hydrolysis reactions in aqueous amine/CO_2 solutions. *Int. J. Greenh. Gas Con.* **104**, 103175 (2021).

13. H. Jiang, I. Novak, Piperidine–CO_2–H_2O molecular complex. *J. Mol. Struct.* **645**, 177–183 (2003).

14. G. Rochelle, E. Chen, S. Freeman, D. Van Wagener, Q. Xu, A. Voice, Aqueous piperazine as the new standard for CO_2 capture technology. *Chem. Eng. J.* **171**, 725–733 (2011).

15. Y. E. Kim, J. H. Choi, S. C. Nam, Y. I. Yoon, CO_2 absorption characteristics in aqueous K_2CO_3/piperazine solution by NMR spectroscopy. *Ind. Eng. Chem. Res.* **50**, 9306–9313 (2011).

16. S. A. Freeman, J. Davis, G. T. Rochelle, Degradation of aqueous piperazine in carbon dioxide capture. *Int. J. Greenh. Gas Con.* **4**, 756–761 (2010).

17. G. Puxty, R. Rowland, A. Allport, Q. Yang, M. Bown, R. Burns, M. Maeder, M. Attalla, Carbon dioxide postcombustion capture: A novel screening study of the carbon dioxide absorption performance of 76 amines. *Environ. Sci. Technol.* **43**, 6427–6433 (2009).

18. M. Liang, Y. Liu, J. Zhang, F. Wang, Z. Miao, L. Diao, J. Mu, J. Zhou, S. Zhuo, Understanding the role of metal and N species in M@NC catalysts for electrochemical CO_2 reduction reaction. *Appl. Catal. Environ.* **306**, 121115 (2022).

19. Z. Xing, L. Hu, D. S. Ripatti, X. Hu, X. Feng, Enhancing carbon dioxide gas-diffusion electrolysis by creating a hydrophobic catalyst microenvironment. *Nat. Commun.* **12**, 136 (2021).

20. X. Sheng, W. Ge, H. Jiang, C. Li, Engineering Ni─N─C catalyst microenvironment enabling CO_2 electroreduction with nearly 100% CO selectivity in acid. *Adv. Mater.* **34**, e2201295 (2022).

21. M. Mortazavi, K. Tajiri, Effect of the PTFE content in the gas diffusion layer on water transport in polymer electrolyte fuel cells (PEFCs). *J. Power Sources* **245**, 236–244 (2014).

22. W. Zhang, J. Ma, P. Wang, Z. Wang, F. Shi, H. Liu, Investigations on the interfacial capacitance and the diffusion boundary layer thickness of ion exchange membrane using electrochemical impedance spectroscopy. *J. Membr. Sci.* **502**, 37–47 (2016).

23. A. Zanone, D. T. Tavares, J. L. d. Paiva, An FTIR spectroscopic study and quantification of 2-amino-2-methyl-1-propanol, piperazine and absorbed carbon dioxide in concentrated aqueous solutions. *Vib. Spectrosc.* **99**, 156–161 (2018).

24. K. Robinson, A. McCluskey, M. I. Attalla, An FTIR spectroscopic study on the effect of molecular structural variations on the CO_2 absorption characteristics of heterocyclic amines. *ChemPhysChem* **12**, 1088–1099 (2011).

25. J. Thompson, H. Richburg, K. Liu, Thermal degradation pathways of aqueous diamine CO_2 capture solvents. *Energy Procedia* **114**, 2030–2038 (2017).

26. T. Nguyen, M. Hilliard, G. Rochelle, Volatility of aqueous amines in CO_2 capture. *Energy Procedia* **4**, 1624–1630 (2011).

27. L. Ge, H. Rabiee, M. Li, S. Subramanian, Y. Zheng, J. H. Lee, T. Burdyny, H. Wang, Electrochemical CO_2 reduction in membrane-electrode assemblies. *Chem* **8**, 663–692 (2022).

28. Y. C. Xiao, S. S. Sun, Y. Zhao, R. K. Miao, M. Fan, G. Lee, Y. Chen, C. M. Gabardo, Y. Yu, C. Qiu, Z. Guo, X. Wang, P. Papangelakis, J. E. Huang, F. Li, C. P. O'Brien, J. Kim, K. Han, P. J. Corbett, J. Y. Howe, E. H. Sargent, D. Sinton, Reactive capture of CO_2 via amino acid. *Nat. Commun.* **15**, 7849 (2024).

29. Y. Kim, E. W. Lees, C. Donde, A. M. L. Jewlal, C. E. B. Waizenegger, B. M. W. de Hepcée, G. L. Simpson, A. Valji, C. P. Berlinguette, Integrated CO_2 capture and conversion to form syngas. *Joule* **8**, 3106–3125 (2024).

30. H. Shin, K. U. Hansen, F. Jiao, Techno-economic assessment of low-temperature carbon dioxide electrolysis. *Nat. Sustainability* **4**, 911–919 (2021).

31. A. F. Ciftja, A. Hartono, H. F. Svendsen, ^{13}C NMR as a method species determination in CO_2 absorbent systems. *Int. J. Greenh. Gas Con.* **16**, 224–232 (2013).

32. M. Nitta, M. Hirose, T. Abe, Y. Furukawa, H. Sato, Y. Yamanaka, ^{13}C-NMR spectroscopic study on chemical species in piperazine−amine−CO_2−H_2O system before and after heating. *Energy Procedia* **37**, 869–876 (2013).

33. M. Xiao, D. Cui, Q. Yang, Z. Liang, G. Puxty, H. Yu, L. Li, W. Conway, P. Feron, Role of mono- and diamines as kinetic promoters in mixed aqueous amine solution for CO_2 capture. *Chem. Eng. Sci.* **229**, 116009 (2021).

34. H. Renon, J. M. Prausnitz, Local compositions in thermodynamic excess functions for liquid mixtures. *AIChE J.* **14**, 135–144 (1968).

35. P. Frailie, J. Plaza, D. Van Wagener, G. T. Rochelle, Modeling piperazine thermodynamics. *Energy Procedia* **4**, 35–42 (2011).

36. Y. Du, G. T. Rochelle, Thermodynamic modeling of aqueous piperazine/*N*-(2-aminoethyl) piperazine for CO_2 capture. *Energy Procedia* **63**, 997–1017 (2014).

37. Q. Lu, C. Chen, Q. Di, W. Liu, X. Sun, Y. Tuo, Y. Zhou, Y. Pan, X. Feng, L. Li, D. Chen, J. Zhang, Dual role of pyridinic-N doping in carbon-coated Ni nanoparticles for highly efficient electrochemical CO_2 reduction to CO over a wide potential range. *ACS Catalysis* **12**, 1364–1374 (2022).

38. W. Luc, J. Rosen, F. Jiao, An Ir-based anode for a practical CO_2 electrolyzer. *Catal. Today* **288**, 79–84 (2017).

39. X. Lu, C. Zhao, Electrodeposition of hierarchically structured three-dimensional nickel–iron electrodes for efficient oxygen evolution at high current densities. *Nat. Commun.* **6**, 6616 (2015).

40. B. Ravel, M. Newville, ATHENA and ARTEMIS: Interactive graphical data analysis using IFEFFIT. *Phys. Scr.* **2005**, 1007 (2005).

41. M. J. Frisch, G. W. Trucks, H. B. Schlegel, G. E. Scuseria, M. A. Robb, J. R. Cheeseman, G. Scalmani, V. Barone, G. A. Petersson, H. Nakatsuji, X. Li, M. Caricato, A. V. Marenich, J. Bloino, B. G. Janesko, R. Gomperts, B. Mennucci, H. P. Hratchian, J. V. Ortiz, A. F. Izmaylov, J. L. Sonnenberg, Williams, F. Ding, F. Lipparini, F. Egidi, J. Goings, B. Peng, A. Petrone, T. Henderson, D. Ranasinghe, V. G. Zakrzewski, J. Gao, N. Rega, G. Zheng, W. Liang, M. Hada, M. Ehara, K. Toyota, R. Fukuda, J. Hasegawa, M. Ishida, T. Nakajima, Y. Honda, O. Kitao, H. Nakai, T. Vreven, K. Throssell, J. A. Montgomery Jr., J. E. Peralta, F. Ogliaro, M. J. Bearpark, J. J. Heyd, E. N. Brothers, K. N. Kudin, V. N. Staroverov, T. A. Keith, R. Kobayashi, J. Normand, K. Raghavachari, A. P. Rendell, J. C. Burant, S. S. Iyengar, J. Tomasi, M. Cossi, J. M. Millam, M. Klene, C. Adamo, R. Cammi, J. W. Ochterski, R. L. Martin, K. Morokuma, O. Farkas, J. B. Foresman, D. J. Fox, GaussView 5.0 (Gaussian, Inc., 2016).

42. A. V. Marenich, C. J. Cramer, D. G. Truhlar, Universal solvation model based on solute electron density and on a continuum model of the solvent defined by the bulk dielectric constant and atomic surface tensions. *J. Phys. Chem. B.* **113**, 6378–6396 (2009).

43. G. Kresse, J. Furthmüller, Efficient iterative schemes for ab initio total-energy calculations using a plane-wave basis set. *Phys. Rev. E* **54**, 11169–11186 (1996).

44. P. E. Blöchl, Projector augmented-wave method. *Phys. Rev. E* **50**, 17953–17979 (1994).

45. J. P. Perdew, K. Burke, M. Ernzerhof, Generalized gradient approximation made simple. *Phys. Rev. Lett.* **77**, 3865–3868 (1996).

46. C. Shang, Z.-P. Liu, Stochastic surface walking method for structure prediction and pathway searching. *J. Chem. Theor. Comput.* **9**, 1838–1845 (2013).

47. X.-T. Xie, Z.-X. Yang, D. Chen, Y.-F. Shi, P.-L. Kang, S. Ma, Y.-F. Li, C. Shang, Z.-P. Liu, LASP to the future of atomic simulation: Intelligence and automation. *Precis. Chem.* **2**, 612–627 (2024).

48. S.-D. Huang, C. Shang, P.-L. Kang, X.-J. Zhang, Z.-P. Liu, LASP: Fast global potential energy surface exploration. *WIREs Comput. Mol. Sci.* **9**, e1415 (2019).

49. V. G. Grigoryan, M. Springborg, A theoretical study of the structure of Ni clusters (NiN). *Phys. Chem. Chem. Phys.* **3**, 5135–5139 (2001).

50. A. Granja-DelRío, H. A. Abdulhussein, R. L. Johnston, DFT-based global optimization of sub-nanometer Ni–Pd clusters. *J. Phys. Chem. C* **123**, 26583–26596 (2019).

51. H. Zhu, X. Li, N. Shi, X. Ding, Z. Yu, W. Zhao, H. Ren, Y. Pan, Y. Liu, W. Guo, Density functional theory study of thiophene desulfurization and conversion of desulfurization products on the Ni(111) surface and Ni55 cluster: Implication for the mechanism of reactive adsorption desulfurization over Ni/ZnO catalysts. *Cat. Sci. Technol.* **11**, 1615–1625 (2021).

52. K. Momma, F. Izumi, VESTA: A three-dimensional visualization system for electronic and structural analysis. *J. Appl. Cryst.* **41**, 653–658 (2008).

53. Y. Mao, Z. Wang, H.-F. Wang, P. Hu, Understanding catalytic reactions over zeolites: A density functional theory study of selective catalytic reduction of NO_x by NH_3 over Cu-SAPO-34. *ACS Catal.* **6**, 7882–7891 (2016).

54. C. Lee, W. Yang, R. G. Parr, Development of the Colle-Salvetti correlation-energy formula into a functional of the electron density. *Phys. Rev. E* **37**, 785–789 (1988).

55. F. Weigend, R. Ahlrichs, Balanced basis sets of split valence, triple zeta valence and quadruple zeta valence quality for H to Rn: Design and assessment of accuracy. *Phys. Chem. Chem. Phys.* **7**, 3297–3305 (2005).

56. F. Weigend, Accurate Coulomb-fitting basis sets for H to Rn. *Phys. Chem. Chem. Phys.* **8**, 1057–1065 (2006).

57. S. Grimme, J. Antony, S. Ehrlich, H. Krieg, A consistent and accurate ab initio parametrization of density functional dispersion correction (DFT-D) for the 94 elements H-Pu. *J. Chem. Phys.* **132**, 154104 (2010).

58. J. Klimeš, D. R. Bowler, A. Michaelides, Van der Waals density functionals applied to solids. *Phys. Rev. E* **83**, 195131 (2011).

59. H.-J. Song, S. Park, H. Kim, A. Gaur, J.-W. Park, S.-J. Lee, Carbon dioxide absorption characteristics of aqueous amino acid salt solutions. *Int. J. Greenh. Gas Con.* **11**, 64–72 (2012).

60. H. Karlsson, H. Svensson, Rate of absorption for CO_2 absorption systems using a wetted wall column. *Energy Procedia* **114**, 2009–2023 (2017).

61. J. H. Kim, H. Jang, G. Bak, W. Choi, H. Yun, E. Lee, D. Kim, J. Kim, S. Y. Lee, Y. J. Hwang, The insensitive cation effect on a single atom Ni catalyst allows selective electrochemical conversion of captured CO_2 in universal media. *Energ. Environ. Sci.* **15**, 4301–4312 (2022).

62. H. B. Yang, S.-F. Hung, S. Liu, K. Yuan, S. Miao, L. Zhang, X. Huang, H.-Y. Wang, W. Cai, R. Chen, J. Gao, X. Yang, W. Chen, Y. Huang, H. M. Chen, C. M. Li, T. Zhang, B. Liu, Atomically dispersed Ni(i) as the active site for electrochemical CO_2 reduction. *Nat. Energy* **3**, 140–147 (2018).

63. E. N. Fuller, J. C. Giddings, A comparison of methods for predicting gaseous diffusion coefficients. *J. Chromatogr. Sci.* **3**, 222–227 (1965).

64. E. D. Snijder, M. J. M. Te Riele, G. F. Versteeg, W. P. M. Van Swaaij, Diffusion coefficients of several aqueous alkanolamine solutions. *J. Chem. Eng. Data* **38**, 475–480 (1993).

65. G. F. Versteeg, W. P. M. Van Swaaij, Solubility and diffusivity of acid gases (carbon dioxide, nitrous oxide) in aqueous alkanolamine solutions. *J. Chem. Eng. Data* **33**, 29–34 (1988).

66. L.-C. Chang, T.-I. Lin, M.-H. Li, Mutual diffusion coefficients of some aqueous alkanolamines solutions. *J. Chem. Eng. Data* **50**, 77–84 (2005).

67. C. G. Zoski, J. Leddy, A. J. Bard, L. R. Faulkner, H. S. White, *Electrochemical Methods: Fundamentals and Applications, 3e Student Solutions Manual* (Wiley, 2024).

68. F. Chaves, Newman and balsara on electrochemical systems fourth edition. *Electrochem. Soc. Interface* **30**, 11–12 (2021).

69. G. Richner, G. Puxty, Assessing the chemical speciation during CO_2 absorption by aqueous amines using in situ FTIR. *Ind. Eng. Chem. Res.* **51**, 14317–14324 (2012).

70. G. T. Rochelle, Amine scrubbing for CO_2 capture. *Science* **325**, 1652–1654 (2009).

71. A. Cousins, S. Huang, A. Cottrell, P. H. M. Feron, E. Chen, G. T. Rochelle, Pilot-scale parametric evaluation of concentrated piperazine for CO_2 capture at an Australian coal-fired power station. *Greenh. Gaes. Sci. Technol.* **5**, 7–16 (2015).

72. M. Bui, C. S. Adjiman, A. Bardow, E. J. Anthony, A. Boston, S. Brown, P. S. Fennell, S. Fuss, A. Galindo, L. A. Hackett, J. P. Hallett, H. J. Herzog, G. Jackson, J. Kemper, S. Krevor, G. C. Maitland, M. Matuszewski, I. S. Metcalfe, C. Petit, G. Puxty, J. Reimer, D. M. Reiner, E. S. Rubin, S. A. Scott, N. Shah, B. Smit, J. P. M. Trusler, P. Webley, J. Wilcox, N. M. Dowell, Carbon capture and storage (CCS): The way forward. *Energ. Environ. Sci.* **11**, 1062–1176 (2018).

73. J. C. Bui, E. W. Lees, D. H. Marin, T. N. Stovall, L. Chen, A. Kusoglu, A. C. Nielander, T. F. Jaramillo, S. W. Boettcher, A. T. Bell, A. Z. Weber, Multi-scale physics of bipolar membranes in electrochemical processes. *Nat. Chem. Eng.* **1**, 45–60 (2024).

74. A. V. Rayer, K. Z. Sumon, L. Jaffari, A. Henni, Dissociation constants (pKa) of tertiary and cyclic amines: structural and temperature dependences. *J. Chem. Eng. Data* **59**, 3805–3813 (2014).

75. N. Bonanos, B. C. H. Steele, E. P. Butler, J. R. Macdonald, W. B. Johnson, W. L. Worrell, G. A. Niklasson, S. Malmgren, M. Strømme, S. K. Sundaram, M. C. H. McKubre, D. D. Macdonald, G. R. Engelhardt, E. Barsoukov, B. E. Conway, W. G. Pell, N. Wagner, C. M.

Roland, R. S. Eisenberg, “Applications of Impedance Spectroscopy,” in *Impedance Spectroscopy* (John Wiley & Sons, Ltd, 2018), pp. 175–478.

76. Y. Kim, M. Namdari, A. M. L. Jewlal, Y. Chen, D. J. D. Pimlott, M. Stolar, C. P. Berlinguette, Economic viability of integrated CO_2 capture and conversion. *ACS Energy Lett.* **10**, 403–409 (2025).

77. H. M. Almajed, R. Kas, P. Brimley, A. M. Crow, A. Somoza-Tornos, B.-M. Hodge, T. E. Burdyny, W. A. Smith, Closing the loop: Unexamined performance trade-offs of integrating direct air capture with (Bi)carbonate electrolysis. *ACS Energy Lett.* **9**, 2472–2483 (2024).

78. B. Belsa, L. Xia, V. Golovanova, B. Polesso, A. Pinilla-Sánchez, L. San Martín, J. Ye, C.-T. Dinh, F. P. García de Arquer, Materials challenges on the path to gigatonne CO_2 electrolysis. *Nat. Rev. Mater.* **9**, 535–549 (2024).

79. S. Ren, D. Joulié, D. Salvatore, K. Torbensen, M. Wang, M. Robert, C. P. Berlinguette, Molecular electrocatalysts can mediate fast, selective CO_2 reduction in a flow cell. *Science* **365**, 367–369 (2019).

80. M. D. Porosoff, B. Yan, J. G. Chen, Catalytic reduction of CO_2 by H_2 for synthesis of CO, methanol and hydrocarbons: Challenges and opportunities. *Energ. Environ. Sci.* **9**, 62–73 (2016).
